# Achieving Exceptionally Enhanced Thermal Conductivity and Bulk Modulus in Polar Insulators Via Modification of Chemical Bonding

**DOI:** 10.1021/acs.jpclett.5c01476

**Published:** 2025-08-21

**Authors:** Niraj Bhatt, Sandip Thakur, Pravin Karna, Ashutosh Giri

**Affiliations:** Department of Mechanical, Industrial, and Systems Engineering, 4260 University of Rhode Island, Kingston, Rhode Island 02881, United States

## Abstract

Materials capable of altering their physical properties in response to external stimuli are highly desirable for a wide range of applications. In particular, materials that exhibit substantial changes in thermal conductivity hold promise for advanced thermal management systems including thermal diodes, rectifiers, and switches. Despite significant interest, achieving substantial tunability in thermal transport has remained a challenge, with current approaches, primarily based on phase change materials, typically limited to ∼ 4× changes in thermal conductivity. Here, by employing first-principles-based atomistic simulations, we demonstrate that pressure can be used to modulate the thermal conductivity of lithium halides by up to 2 orders of magnitude, enabling a transition from a thermal insulator to an efficient heat conductor within the same material system. This exceptional tunability arises from pressure-induced changes in their chemical bonding, inducing a transition from predominantly ionic to more covalent character, and their polar nature, characterized by a significant reduction in the longitudinal-optical and transverse-optical phonon mode splitting at the Brillouin zone center. These changes lead to unique anharmonic phonon scattering behavior, which underlies the extraordinary thermal transport modulation. Moreover, our results also show exceptional enhancements in the bulk modulus by as much as 15-fold at pressures near 90 GPa for the lithium halides. Our findings highlight the strong coupling between chemical bonding and vibrational dynamics in polar insulators and open new pathways for designing stimuli-responsive materials with highly tunable macroscopic properties. These insights hold potential for a wide array of applications, including next-generation biomedical devices, sensors, and thermal circuits.

## Graphical abstract

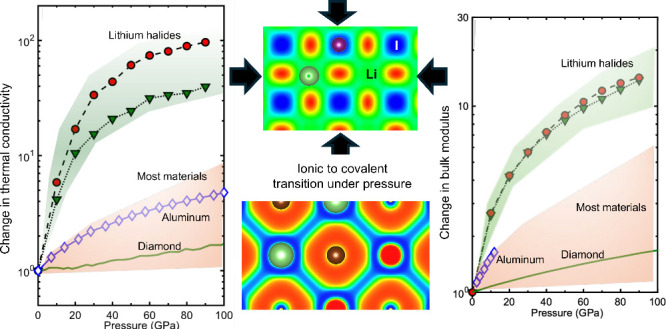

The macroscopic properties of materials are closely tied to the nature of chemical bonding between the constituent elements. For example, the extent of orbital hybridization in covalent materials and the strength of electrostatic forces in ionic solids have profound influences on their mechanical and thermal properties. In many applications requiring control over such macroscopic properties of materials, drastic changes in the chemical makeup (that can be triggered through exposure to external stimuli) are required to induce large changes in the physical properties. This is crucial none more so than for applications in thermal switches, diodes, and regulators, requiring sizable changes in the thermal conductivity of materials;^1–3^ for practical applications, one would ideally seek for materials where thermal transport characteristics can be toggled between that of a heat insulator (where the atoms are usually held together with weaker interactions) and an efficient thermal conductor (where relatively stiff covalent bonds exist between the atoms). However, drastic modifications to the chemical makeup to achieve such large changes in thermal transport properties have remained elusive thus far, and current strategies to achieve changes in thermal transport are mostly reliant on phase change materials that are mostly limited to ∼ 4× changes in thermal conductivity.^1,4^

In general, thermal transport in crystalline insulators and semiconductors, where heat is primarily carried by phonons, has been a topic of considerable interest not just from a materials science perspective but also from an applicative standpoint.^5–7^ For instance, recent insights from first-principles calculations of thermal conductivity have led to the identification of new high thermal conductivity materials such as BAs and cubic BN, where low-wavevector acoustic phonons have long lifetimes and mean free paths.^8–12^ These materials with high thermal conductivities (even rivaling diamond) are promising candidates for applications in heat dissipation of microelectronics.^13^ At the other extreme, ultralow thermal conductivity materials have been engineered with enhanced phonon scattering mechanisms and extreme vibrational localizations.^14–18^ These thermally insulative materials are useful for various purposes involving reduced heat transfer such as in thermal barrier coatings and thermoelectric applications. However, toggling between a thermal insulator and an efficient heat conductor in the same material system (through exposure to an external stimulus such as light illumination or strain engineering) has remained a difficult task, particularly because of the challenge of modifying the broadband phonon spectrum that controls the thermal conductivity of materials.^1,2,4^ Realizing this could have major impacts in novel strategies to control thermal transport, finding applications in the next generation of nonlinear and active thermal circuits.^1^

In this regard, some of the largest changes in thermal conductivity have been demonstrated in various material systems including: azobenzene polymers, where thermal transport has been shown to vary by 3× through light-induced phase change;^19^ reversible delithiation in lithium cobalt oxide has also been shown to lead to similar thermal conductivity changes;^20^ wetting of tandem repeat proteins in squid ring teeth-based biopolymers has been associated with a rapid 4× change in thermal conductivity;^2^ pressure-driven changes in thermal conductivity for semiconductors are also on par with such changes,^21–23^ while for metals, thermal conductivity changes of 8× under extreme pressures (of ∼ 200 GPa) have also been reported.^24^ Here, we show an unprecedented ∼ 100× change in thermal conductivity for polar insulators (namely, lithium halides) through the application of hydrostatic pressures (as high as 90 GPa). We attribute this exceptional transition from a thermal insulator to an efficient heat conductor in lithium halides to variations in the intrinsic broadband phonon properties (including their anharmonic scattering mechanisms) and the unique modification of their chemical bonding realized through strain engineering. We note that while such pressures are not directly applicable for thermal switching applications, our findings provide insight into the underlying mechanisms, particularly bonding environment changes, that could be leveraged in future studies to develop dynamic thermal materials using more practical stimuli (e.g., light, electric fields, or strain).^1,25,26^

Under ambient conditions, lithium halides are polar materials where long-range Coulomb interactions between the ions can drastically influence the optical phonon modes and their thermal transport properties. More specifically, the Born effective charges and high dielectric constants result in a nonanalytical term in the dynamical matrix,^27^ which dictates the splitting of the longitudinal optical (LO) and transverse optical (TO) modes at the center of the Brillouin zone (BZ), distinguishing polar solids from their nonpolar counterparts.^28^ Typically, optical phonons have small group velocities and do not carry a significant amount of heat. However, in polar materials with strong Coulomb interactions, the LO branch is pushed to higher frequencies, resulting in the splitting of the LO-TO modes (and thus providing a broader frequency spectrum for the optical phonons). Although optical phonons typically do not facilitate heat conduction, their frequency spectrum (which can control the phase space for phonon scattering) influences the overall thermal resistance by increasing the probability for the acoustic modes to scatter with optical phonons. In fact, the gap between the acoustic and optical modes in binary compounds has been shown to crucially dictate their thermal transport properties.^8,10–12^ For instance, in BAs, the high room-temperature thermal conductivity has been shown to result from the large frequency gap between acoustic and optical phonons, which is large enough to freeze out the resistive scattering processes involving two acoustic phonons and an optical phonon.^8^ In this analogy, ways to engineer the LO-TO splitting and the broadband optical phonon energies in polar compounds can potentially be an efficient strategy to control their thermal conductivity by modifying the large phase space for scattering of the heat carrying acoustic phonons.

In this work, we demonstrate that the frequency spectrum of the LO-TO splitting in polar insulators can be efficiently manipulated via the application of hydrostatic pressures, leading to significant changes in the broad-band phonon scattering mechanisms. This ultimately results in the transition of the lithium halides from heat insulators (at ambient) to heat conductors (under extreme pressure conditions), a phenomenon that has never been observed experimentally or predicted theoretically for any known material system. To this end, we utilize a combination of density functional theory (DFT) calculations, machine learning-assisted molecular dynamics (MD) simulations, and anharmonic lattice dynamics calculations to study the unique pressure-driven thermal transport properties of lithium bromide and lithium iodide (the prototypical polar insulators). We show that the application of pressure can drastically reduce the LO-TO splitting and lead to massive reductions in the values of the dielectric constants for these solids. As a consequence, the chemical makeup of the compounds is uniquely altered from ionic bonding-dominated to a more covalent nature of the bonds with the application of pressure. This change in the bonding environment results in an unprecedented ∼ 100× change in the thermal conductivity and an exceptional ∼ 15× increase in the bulk modulus. We ascribe the enhanced thermal transport in the lithium halides to the unusual response of the microscopic phonon scattering mechanisms to pressure, which is crucially dictated by the gradual narrowing of the optical phonon spectrum and the systematic increase in the gap between the optical and acoustic phonons with pressure. Our results shed light on the mechanistic processes controlling the massive changes in the thermal and mechanical properties of lithium halides with pressure, setting them up as stimuli-responsive materials for use in the next-generation of thermal logic circuits and thermal management strategies.

We employ first-principles-based machine learning interatomic potentials in our MD simulations to accurately and fully capture the higher-order phonon scattering mechanisms, which can become increasingly important in dictating the thermal conductivity, especially for extreme conditions.^29,30^ We calculate the thermal conductivity of our lithium halide domains via the Green–Kubo formalism under the equilibrium MD framework;^31^ details of the development for the different machine learning-based interatomic potentials, MD simulation procedures, DFT calculations, and spectral analyses are given in the Methods section and the Supporting Information.

Our main results, summarized in Figure 1, show our calculations of thermal conductivity (Figure 1a,b) and bulk modulus (Figure 1c,d) as a function of applied hydrostatic pressures for LiBr and LiI. We also compare our results to other materials where large changes in these properties have been achieved through high pressure conditions. While it is expected that both the thermal conductivity and bulk modulus of solids generally increase with applied hydrostatic pressures, the enhancement in these properties for the lithium halides studied in this work are exceptionally larger in comparison to other materials; although these physical properties of isotopically pure diamond represent some of the highest attainable values for the entire pressure range shown in Figure 1, there is a modest increase (by only ∼ 2× for both thermal conductivity and bulk modulus) at high pressure conditions (of ∼ 100 GPa) as compared to the ambient value for diamond.^22^ Similar monotonic increases in these properties are also reported for cubic BN.^30^ These modest enhancements in these physical properties have mainly been attributed to stiffening of the bonds along with the hardening of the phonon modes; for thermal conductivity, it is also attributed to increments in the acoustic phonon group velocities and reduction in anharmonic phonon–phonon scattering processes for these high thermal conductivity solids. In comparison, pressurized lithium halides can achieve unprecedented enhancements of up to ∼ 100× in thermal conductivity and ∼ 15× in bulk modulus for pressures of ∼ 90 GPa. Note, since lithium bromide has a structural phase transition at 94 GPa,^33^ we limit our calculations to 90 GPa pressure conditions in this work.

**Figure 1:**
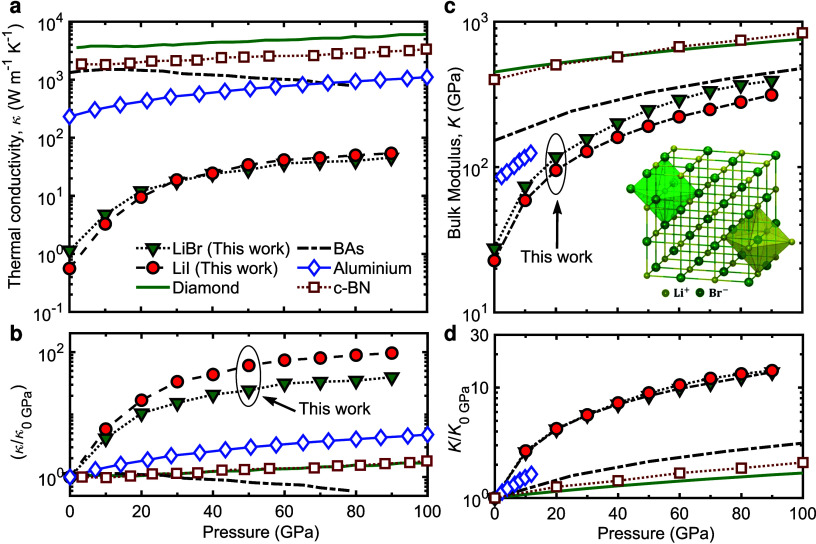
**Changes in thermal conductivity and bulk modulus under pressure.** (a) The application of hydrostatic pressure can convert lithium iodide and lithium bromide from thermal insulators (with κ < 1 W m^–1^ K^–1^) at ambient to heat conductors (with κ ∼ 50 W m^–1^ K^–1^) under extreme pressure conditions. (b) Comparison of normalized thermal conductivities (relative to the thermal conductivity at 0 GPa) as a function of pressure. The enhancement in thermal conductivity can reach as high as ∼ 100× for pressures of ∼ 90 GPa. These results show that the thermal conductivity response to pressure is drastically higher in these lithium halides as compared to diamond,^22^ BAs,^32^ aluminum,^24^ and cubic BN.^30^ (c) Similar exceptional enhancement in the bulk modulus (by as much as ∼ 15×) is also realized in these lithium halides (for pressures reaching ∼ 90 GPa). (d) While some materials can demonstrate as much as ∼ 3× enhancement in bulk modulus, the ∼ 15× increments that we observe for our lithium halides are the largest changes in the mechanical properties for any known crystal.

The massive enhancements in the physical properties of lithium halides with the application of hydrostatic pressure are mainly related to the unique changes in the chemical bonding characteristics. As shown in the electron localization functions (ELFs) in Figure 2a–d, pressure induces variations in the intrinsic nature of the bonding where the degree of covalent character is gradually increased with increasing pressure. Note, the ELF values range from 0 to 1, where 0 is related to no electrons, 0.5 represents states that are free electron-gas-like, and 1 represents the highest localization of electrons.^34^ Although both halides exhibit strong ionic bonding characteristics at lower pressures, with comparatively reduced electron densities around the lithium ion and strong localization around the halide ions, there is a gradual enhancement in electron density delocalization with pressure, indicating an increasing proportion of covalent bonding characteristics in these halides. Comparing the changes in the ELFs between the two halides, the stronger polarizability of the comparatively bigger halogen ions in lithium iodide leads to a larger change in their chemical binding nature, which ultimately drives the relatively larger increase in the thermal conductivity of lithium iodide compared to lithium bromide.

**Figure 2:**
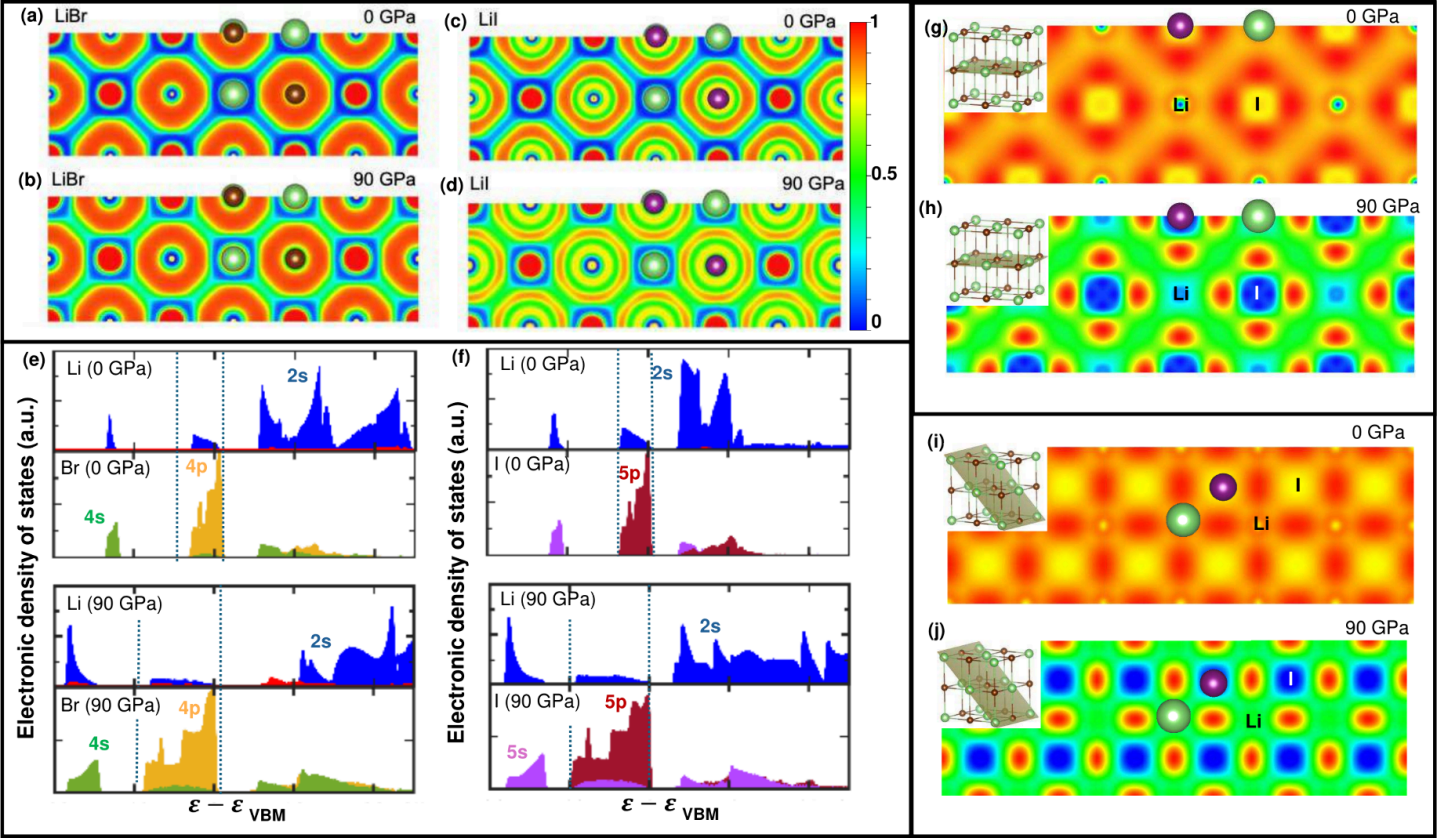
**Changes in the chemical bonding with pressure.** For (a-b) LiBr and (c-d) LiI, while the electron densities are mainly surrounded around the halide ion at ambient, they are gradually delocalized as the pressure is increased. This suggests that the halides transition from an ionic solid to one with a more covalent nature to the bonding characteristics. This is supported through electronic density of states calculations showing an increased level of hybridization between the 2*s* orbitals of lithium and the *p* orbitals of the halides in (e) LiBr and (f) LiI. (g-j) The charge density difference between a hypothetical crystal for lithium iodide that is put together from superposing the charge densities of free atoms and the charge density calculated for LiI unit cells. The visualization planes are shown in the insets. A clear directionality in the bonding is observed at higher pressures, indicating a more covalent nature of the bonds.

As exemplified by the changes in the ELFs, the delocalization of electron densities with increasing pressure is a consequence of increased hybridization (and overlap in the orbitals) between the different electronic states in the lithium and the halide ions (Figure 2e,f). For example, the 2*s* orbital of Li shows an increased overlap in energy with the 4*p* (5*p*) orbitals of bromine (iodine) ions at 90 GPa as compared to that of the ambient case (Figure 2e). Thus, with increasing pressure, the greater orbital hybridization leads to a stronger covalent character of the bonds in these lithium halides.

Although the comparison between the changes in the ELFs and the electronic density of states can provide some fundamental insights into the nature of bonding, the specific distinguishing difference between ionic and covalent bonds (in general) is the bond directionality; in other words, charges tend to accumulate more-so along stronger bonding directions distinguishing covalent bonds from ionic interactions.^34^ However, such a small charge accumulation is not clearly visible in the ELFs. Therefore, instead, we inspect the density difference between the charge density of a hypothetical crystal that is put together by superposing the charge densities of free atoms and the charge density calculated for our unit cells. This charge density difference will show how the electrons are redistributed when the initially free atoms interact to form the lithium halide crystals and how the pressure can influence the spatial redistribution of electrons in these materials.

Figure 2g–j shows the charge density differences along two different planes (shown in the insets) for lithium iodide at 0 and 90 GPa (similar changes are observed for lithium bromide shown in Figure S23). The application of pressure leads to greater bond directionality in the pressurized cases, where the charge density differences are more located in the vicinity of the neighboring atoms. For the unpressurized case, the charge density that binds the nuclei is more localized around the halogen ions. In contrast, the charge density that binds the nuclei is shared between them for the pressurized case, which further emphasizes the role of pressure in dictating the significant changes in the chemical binding nature (and the concomitant changes in the macroscopic physical properties) of these solids.

Covalent bonding strength can be increased either by enhancing spatial overlap or by narrowing the energy difference between the parent atomic orbitals.^35,36^ Consequently, a greater orbital overlap is positively linked to a higher bulk modulus. Similar increase in bulk modulus due to increase in covalent bonding characteristic has been previously shown in various other studies.^37–39^

It should be noted that along with changes in the bonding character, a recent work has shown that geometry effects such as bond angle and length changes can also significantly change thermal transport properties.^40^ Future studies could explore the effects of uniaxial or shear strain, which—unlike the hydrostatic compression examined in our work—would alter bond lengths differently and offer insight into how such distortions influence the thermal properties of lithium halides.

To better understand the influence of chemical bonding changes on the intrinsic processes dictating the macroscopic physical properties of these metal halides, we first show the changes in the phonon dispersion relations and vibrational energies resulting from the application of pressure in Figure 3a–f. The polar nature of the metal halides at ambient pressure manifests in the splitting of the LO-TO branches at the center of the BZ. As noted above, this is the distinguishing characteristic of polar compounds originating from the lattice dynamics of the LO phonons that generate extra long-range electric fields. This in turn leads to the long-range Coulomb forces on the polar lattices, raising the energies of the LO phonons at the center of the BZ and resulting in the splitting with the TO modes. Experimental evidence of this phenomena has been reported for several other polar compounds such as BN,^41^ GaP,^42^ and SiC,^43^ which have relatively higher thermal conductivities (at ambient) as compared to the lithium halides studied in this work.

**Figure 3:**
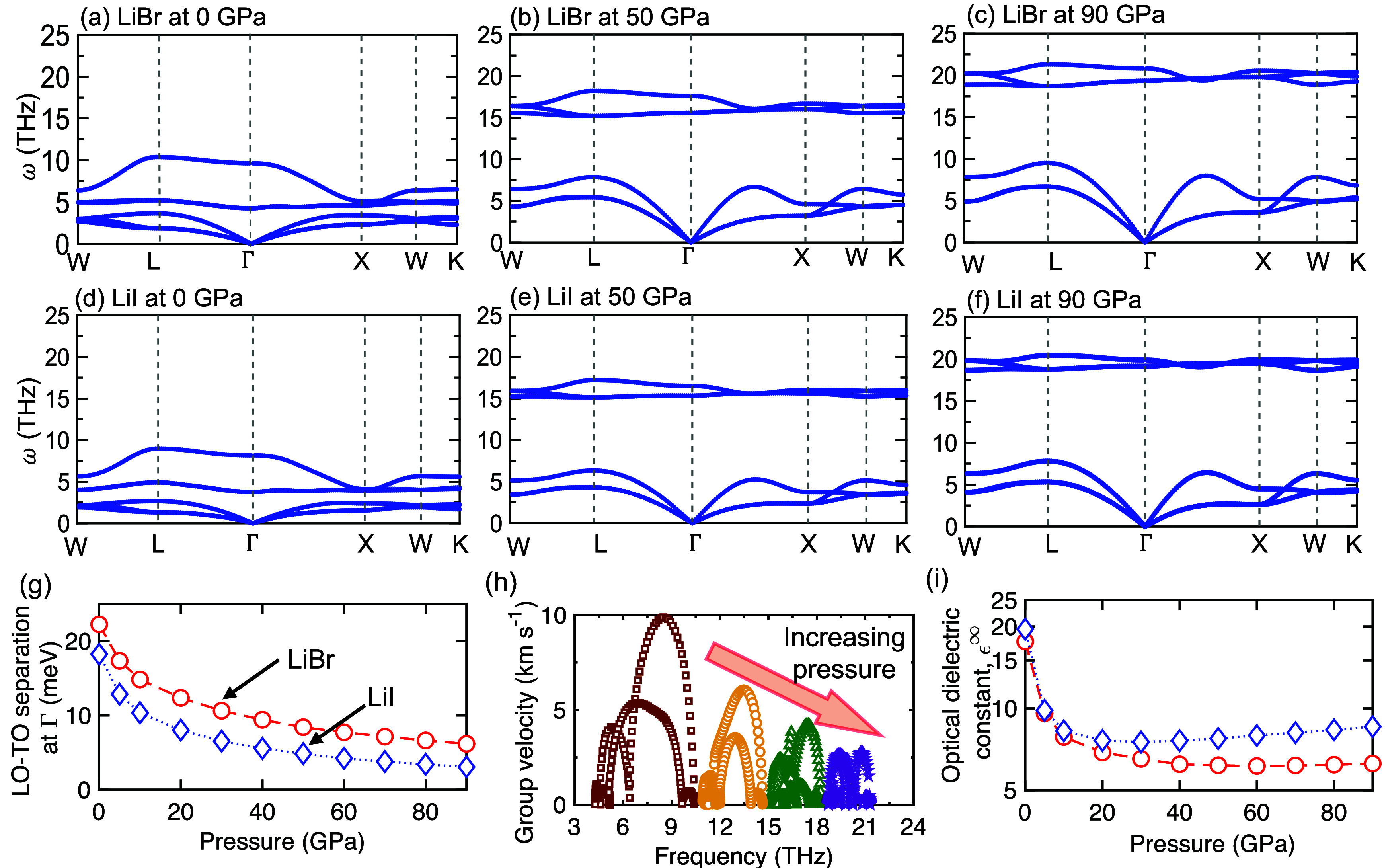
**Changes in the vibrational landscape and optical properties with increasing pressure.** The major influence on the phonon dispersions of (a-c) LiBr and (d-f) LiI with increasing pressure is the increase in the frequency gap between the acoustic and optical phonon modes and the gradual decrease in the splitting energies between the longitudinal optical and transverse optical (LO-TO) phonons (g). Combined with the fact that the optical phonons are gradually flattened with pressure, these changes in the optical energies lead to gradual reduction of the group velocities with increasing pressure as shown for lithium iodide at 0, 20, 50, and 90 GPa pressures (h). Consequently, the dielectric constants also gradually decrease with pressure for lithium halides (i).

From Figure 3a–f, it is clear that the application of pressure on our lithium halides leads to a gradual decrease in the difference between the splitting energies of the LO-TO phonons at the BZ center, which we show in Figure 3g. This suggests that the polar nature of the compounds is strongly affected by pressure; the long-range Coulomb forces are essentially nonexistent if the covalent nature of the bonds is drastically enhanced. It is interesting to note that the monotonic decrease in the splitting of the LO-TO phonons also leads to the gradual flattening of the entire optical modes with the application of pressure. This in turn results in the gradual decrease of the optical phonon group velocities with increasing pressure (Figure 3h). These changes in the optical phonon energies are manifested in a gradual reduction of the dielectric constants of these solids (Figure 3i). Moreover, the largest reductions in the dielectric constants (and the splitting of LO-TO phonons) occur in the pressure range of ≤30 GPa. At this pressure range, we also observe the largest changes in thermal conductivity for our lithium halides (Figure 1a), suggesting a strong correlation between the characteristic polar nature of the solids and thermal transport processes. More specifically, these changes in the polar nature of the solid (driven by the changes in optical phonon energies) play a pivotal role in dictating the mechanistic scattering processes of phonons (as we show in more detail below), which ultimately drives the massive changes in pressure-driven thermal transport properties in these materials.

It is also noteworthy that the decreasing polar nature of the solid with increasing pressure is consistent with the changes in the nature of the bonding where the ionic solids are transitioned to possess a more covalent nature (Figure 2). In this regard, other polar solids that also possess significant LO-TO splitting at ambient conditions do not show such drastic changes in the bonding environment (and the thermal conductivity) with the application of pressure (as confirmed for GaN, SiC, and c-BN in Figure S22).

Now we discuss the influence of chemical binding changes in the intrinsic mechanistic processes driving exceptional enhancement in the physical properties of pressurized lithium halides. While it is expected that the bulk modulus of solids (in general) increases with increasing pressures, since it is a measure of how resistant a material is to changes in volume due to the application of pressure, the increase in thermal conductivity with pressure is not a similar universal phenomena. Specifically, several binary compounds with large mass mismatches between the constituent atomic species can demonstrate decreasing trends in their thermal conductivity as pressure increases.^32^ For example, mass-mismatch solids (with mass ratios of >4), such as BAs, have been shown to possess a nonmonotonic decrease in thermal conductivity at higher pressures.^30,44^ The reason behind this decrease has been attributed to the significant gap between the acoustic and optical modes, which leads to three phonon scattering processes being dominated by acoustic modes, since the phase space for scattering increases as a result of phonon hardening with increasing pressure.^32^ In other words, processes involving three acoustic phonons (as opposed to two acoustic phonons and one optical phonon) dominate the anharmonic processes, since the phase space for scattering of the heat carrying acoustic modes is significantly increased from phonon hardening due to pressure. This leads to the resistive processes that gradually increase with pressure and cause the thermal conductivity to decrease. In contrast, even though the lithium halides have much larger mass ratios compared to BAs, the exceptionally enhanced thermal conductivities at higher pressures suggest that drastically different phonon scattering mechanisms are prevalent in these solids, which is what we will attempt to spectrally analyze in the following calculations.

In the simple kinetic theory picture, thermal conductivity is given as κ = 1/3*Cv*_g_^2^τ, where *C* is the volumetric heat capacity, *v*_g_ is the group velocity of the wave-packets of phonons, and τ is the phonon lifetime. For our lithium halides, the application of pressure hardens the phonons and leads to an increase in the heat capacity with rising pressures, which is typical of crystalline solids. The changes in the group velocities with pressure, however, show very interesting trends that are not typical of pressure-induced changes in solids; while the group velocities of the acoustic modes are increased (Figure S19), as is expected with the stiffening of the bonds in solids, it unconventionally decreases for the optical modes (Figure 3h). This unique behavior is a direct consequence of the diminishing LO-TO splitting levels and the flattening of the optical modes with increasing pressure (Figure 3). As such, the decrease in optical phonon group velocities should result in a reduced contribution from these optical modes to the thermal conductivity at high pressures, whereas the acoustic mode contributions should increase at higher pressures. Although interesting, so far we have only discussed the changes to the harmonic vibrational properties of the crystal, and so, they do not provide insights into the anharmonic processes (such as those that determine τ of the phonon modes) that crucially dictate the thermal conductivity of solids (in general).

To provide insights into the changes in the anharmonic nature of the phonon modes with pressure, we turned to our spectral energy density (SED) calculations (Figure 4a-d). Briefly, the higher contrasts in the shading of the plots are related to the higher magnitudes of the SEDs.^45^ In the absence of anharmonic interactions between vibrational modes, assuming a purely harmonic system, the evaluation of the SED would precisely reproduce the harmonic phonon dispersion.^46^ However, anharmonic effects become evident through broadening of the SED profiles and an increase in the contrast in shading. Thus, the higher contrast in the shading (or the higher magnitude of the SEDs) represents increased lifetimes of those modes. In other words, the modes that appear brighter have higher kinetic energies. Also, the broadening of SEDs suggests larger anharmonicities, stronger scattering, and reduced lifetimes of the phonon modes. More specifically, the lifetime of phonon modes can be approximated by considering the line widths obtained from our SEDs (Figure 4b,d). We find that at ambient conditions, the broadening of the optical modes of the lithium halides demonstrates their much reduced lifetimes as compared to the acoustic modes. However, for high pressure conditions, the optical phonon lifetimes are an order of magnitude higher compared to the ambient case (and on par with the lifetimes of the acoustic modes). We have performed additional SED calculations at elevated temperatures under both ambient and high-pressure conditions. As shown in Figure S26, increasing the temperature results in further broadening of the SEDs, indicating enhanced anharmonicity in the system. This increased anharmonicity leads to stronger phonon scattering and, consequently, reduced thermal conductivity. The effect is even more significant under pressure, as highlighted in Figure S27, showing that the well-defined phonon branches at the low temperature (100 K) are considerably broadened at higher temperatures.

**Figure 4:**
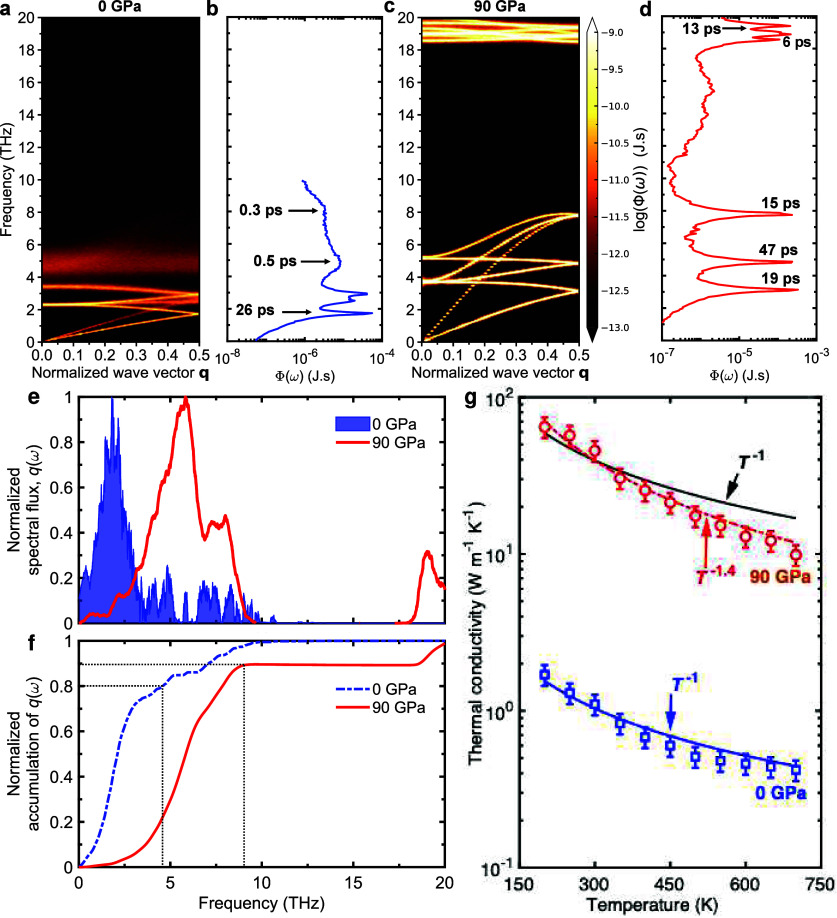
**Unique changes to the intrinsic phonon scattering mechanisms dictating the pressure response of thermal conductivity.** (a-d) Spectral energy density (SED) calculations for LiBr at (a-b) 0 and (c-d) 90 GPa pressures. The broadening in the SEDs indicate higher anharmonicities and reduced lifetimes of the phonon modes. While optical phonons have reduced lifetimes as compared to acoustic modes at ambient pressure, the lifetimes of the optical modes are comparatively longer at higher pressures. However, complementary spectral heat flux calculations (e-f) show that the majority of the contribution to the heat current comes from acoustic phonons at both high and low pressures. For higher pressures, the spectrum of acoustic phonons contributing to thermal conductivity is shifted to higher frequencies. (g) Arising from the broadened phononic spectrum with increased pressure, the higher temperature dependence for the 90 GPa case as compared to the ambient suggests that higher-order anharmonic processes become increasingly important at higher-pressure conditions.

While the scattering rates from our SEDs provide some insights into the lifetimes of the different phonon modes, a detailed calculation of first-principles-based three- and four- phonon scattering may help shed more light on the effects of acoustic phonon bunching and the changes in the acoustic-optic gap on the phonon scattering rates, which is beyond the scope of the current work but deserves further consideration.

Even though the optical modes have higher lifetimes in the pressurized cases, for the heat conduction, the decrease in their group velocities (resulting from the flattening of the modes) outweighs the influence from the enhancements in their lifetimes (see Figures S19 and S29). As such, the thermal conductivity is dominated by the acoustic modes, as evident from our spectral analysis of the heat current in our MD simulations (Figure 4e,f). As shown in Figure 4e for LiBr, we find that the majority of the heat is carried by acoustic modes, with as much as 80% and 90% of the contributions to the heat currents at ambient and 90 GPa pressure conditions, respectively. This shows that the optical modes do not carry any significant amount of heat, more-so in the pressurized cases. The significant role of acoustic modes is further evidenced by the linear correlation observed between pressure and bulk modulus, consistent with the analytical strain-dependent thermal conductivity model proposed by Leibfried and Schlömann,^47^ which predicts κ ∝ *K* ^3/2^, where *K* is the bulk modulus (see Figure S34). This framework assumes that heat transport is primarily governed by acoustic phonons. Therefore, the close agreement between theoretical predictions and molecular dynamics simulations supports the conclusion that acoustic phonons are the primary heat carriers in lithium halides. This is also further supported by our calculations of the three phonon anharmonic scattering rates showing drastically reduced phase spaces for the acoustic modes (see Figure S21), which is a direct consequence of the phonon band gap opening with pressure. This increasing gap between the acoustic and optical modes with increasing pressure leads to the freeze out of anharmonic processes involving two acoustic phonons and one optical phonon due to energy and momentum conservation, thus leading to the monotonic increase in thermal conductivity with pressure. This is in contrast to the monotonic reduction in thermal conductivity predicted for highly mass-mismatched solids with pressure due to the broadening of the phase space for acoustic phonon scattering.^32^ More specifically, Lindsay et al.^32^ predict that when the gap between the acoustic and optical phonons exceeds the maximum acoustic phonon frequency, the phase space for scattering of the acoustic phonons is dominated by the broadening in the acoustic phonon spectrum resulting from higher pressure. However, for our lithium halides, we find that the gradual increment in the gap between the acoustic and optical phonons, which drives the enhanced freeze out condition for acoustic and optical phonon mode scattering, dictates the pressure dependence of thermal conductivity for the pressure range studied in this work.

These intrinsic scattering processes have been shown to dictate the temperature dependence of the thermal conductivity in crystalline semiconductors and insulators. In Figure 4g, we plot the temperature-dependent thermal conductivities of LiBr at ambient and 90 GPa pressure conditions. The κ ∝ *T* ^–1^ dependence for the ambient case suggests the dominance of three phonon processes.^48^ However, for the 90 GPa case, we obtain a higher (κ ∝ *T* ^–1.4^) dependence on temperature, indicating that higher-order phonon scattering mechanisms beyond the three phonon processes dictate heat conduction in the pressurized halides. In this regard, Ravichandran et al.^30^ have shown that the interplay between the reduction in the four-phonon scattering phase space relative to the increase in the three-phonon scattering channels leads to a nonmonotonic dependence on pressure for BAs. For our lithium halides, we find that the spectral dependence shifts to higher acoustic phonon frequencies in the pressurized cases, likely facilitating higher-order processes at higher temperatures. However, unlike for BAs, the monotonic increase in the thermal conductivity with pressure for our lithium halides suggests that the freeze out of acoustic and optical phonon coupling along with the considerable acoustic phonon hardening dominate the heat conduction in these materials for the pressure range studied in this work. Taken together, these intrinsically different anharmonic processes dictating the pressure response of lithium halides help explain the exceptional enhancements in thermal conductivity under extreme pressure conditions.

Finally, we also computed the bulk modulus of the lithium halides across a range of temperatures to further support the observation of a transition from ionic to covalent bonding. At ambient pressure, the bulk modulus shows a linear decrease with increasing temperature, as seen in Figure S17, consistent with typical ionic solids. This trend arises because rising temperatures cause atoms to vibrate more intensely and move further apart on average, leading to thermal expansion and increased compressibility. As a result, the material softens, and the bulk modulus decreases. In contrast, for strongly covalent materials such as diamond, the bulk modulus remains largely unchanged with temperature due to minimal thermal expansion, a result of the robustness of covalent bonds. This behavior is also observed in our high-pressure lithium halide calculations, where the temperature dependence of the bulk modulus diminishes, indicating a shift toward covalent bonding at elevated pressures.

The findings presented in this study highlight the need for experimental validation of thermal and mechanical property changes under high pressure, for instance, using diamond anvil cell (DAC) techniques. However, measurements in diamond anvil cells (DACs) are limited by several factors: the extremely small sample volumes (∼10–100 μm scale) make it difficult to obtain accurate thermal data due to dominant boundary scattering effects;^5^ thermal gradients can be poorly defined due to pressure-induced heterogeneities and nonhydrostatic stresses;^49^ and the constrained geometry introduces additional uncertainties from heat loss through the gasket and diamond anvils. While techniques such as Time-Domain Thermoreflectance (TDTR) and Frequency-Domain Thermoreflectance (FDTR) show promise, they rely on smooth, uniform sample surfaces and stable transducer layers, conditions that lead to diminished signal quality in pump–probe experiments under high-pressure environments.^50^ These challenges highlight the value of computational approaches, such as ours, which allow thermal transport to be investigated under controlled, idealized conditions that are difficult to replicate experimentally.

In summary, we employ a combination of first-principles calculations and molecular dynamics simulations to demonstrate exceptional changes in the bulk modulus (∼15×) and thermal conductivity (∼100×) in lithium halides with hydrostatic pressures of up to 90 GPa. We mainly ascribe these massive enhancements in the physical properties to changes in the chemical bonding characteristics and phonon dispersion relations in these solids. The large splitting of the LO-TO branches at the center of the Brillouin zone and the high dielectric constants, which are characteristics of highly polar solids, are shown to gradually decrease with increasing pressures. Conducting a spectral analysis of the changes in the phonon properties shows that the response of the intrinsic phonon scattering processes is distinctly different in these polar solids as compared to their nonpolar counterparts; uniquely, this response is triggered by the gradual opening of the gap between the acoustic and optical phonon modes along with the shrinking of the optical phonon bandwidth with increasing pressure, which ultimately has a drastic influence on the overall thermal conductivity of the lithium halides. Our results show that with control over the unique splitting of the LO-TO phonons and the optical phonon energies, one can tune the thermal conductivity of lithium halides across a wide range, whereby these materials can even transition from a thermal insulator to an efficient heat conductor.

## Methods

### Machine Learning-Assisted Development of Interatomic Potentials for Molecular Dynamics Simulations

We develop machine learning-assisted interatomic potentials for our lithium halides for a range of temperature (200 to 700 K) and pressure (0 to 90 GPa) conditions. More specifically, we construct five interatomic potentials for LiBr and LiCl that span the temperature and pressure ranges separately. For example, two of these potentials are specifically developed to describe the temperature dependences of the lithium halides at ambient pressure. Similarly, two different interatomic potentials are created to examine the pressure dependences of each lithium halide up to 90 GPa at room temperature. The fifth interatomic potential is focused on studying the temperature dependence at 90 GPa specifically for LiBr.

To sample the atomic configurational space of lithium halides under the diverse range of temperatures and pressures, we perform *ab initio* MD simulations using the Quantum Espresso package.^51^ The configurational parameters like coordinates and cell parameters and their corresponding *ab initio* energies and *ab initio* forces are sampled to construct the training data set. Diverse snapshots of *ab initio* trajectories across the range of temperatures and pressures are generated. An energy cutoff of 60 Ry is taken for plane wave expansion in all *ab initio* MD simulations. The convergence of SCF energies for the cutoffs in the range of 60 to 100 Ry ensures the choice of our cutoff (60 Ry) for plane wave energy expansion (see Figure S1). Similarly, a convergence threshold of 1 × 10^–6^ Ry is used to sample the *ab initio* energies accurately. We use a 4 × 4 × 4 Monkhorst–Pack mesh to sample the Brillouin zone in all of our *ab initio* MD simulations. The Gaussian smearing is used to account for the temperatures ranging from 200 to 700 K. A supercell size of 2 × 2 × 2 is used in all *ab initio* simulations. Each MD simulation is preceded by a cell-relaxation using an isobaric–isothermal ensemble. Born–Oppenheimer dynamics based *ab initio* MD simulations provide the forces by minimizing Kohn–Sham energy functional with respect to the atomic configuration at the specific time step. A time step of 2 fs is used in all of our *ab initio* simulations. The pressure is held constant using using Parrinello–Rahman barostat,^52^ and thermostatting is achieved using rescaling technique. For the temperature-based potentials, the temperature is increased gradually from 200 to 700 K with a step of 100 K. A total of 6,000 dataframes are sampled for constructing the temperature-based potentials, which we split into training and validation data sets. The validation data set comprising 600 dataframes is not included in the training data set and is used to infer the accuracy of the temperature-based potentials. Similarly, for the pressure-based potentials, a total of 10,000 dataframes are sampled, out of which 1,000 dataframes are used for validation data set. The validation data set consists of sample data sets across the temperature and pressure ranges to ensure that the validation accuracy is uniform over the entire sampled data set.

We have used a deep learning framework (DeepMD)^53^ to map the configurational space of atoms with their corresponding energy space. A Deep potential smooth edition (or DeepPot-SE) neural model is constructed by training the sampled data set wherein both radial and angular components are used to produce the descriptors for training. The DeepMD framework constituting of TENSORFLOW is used for training, saving, and testing the machine-learned potential (MLP). The MLP is used subsequently for MD simulations performed with the Large-scale Atomic/Molecular Massively Parallel Simulator (LAMMPS) package.^54^

The cutoff radius is chosen to be 6 Å, and the descriptors decay from 0.5 to 6 Å to eradicate any discontinuity introduced by the cutoff. Our neural architecture is composed of two different neural networks known as the embedding network and the fitting network. The embedding network transforms the atomic coordinates to descriptors, and the fitting network maps the descriptors to their corresponding atomic energies. The embedding network constitutes three hidden layers with sizes of 25, 50, and 100 neurons. It follows Res-Net-like architecture^55^ while the fitting network consists of three hidden layers with 240 neurons in each of the layers. The cost function is optimized using Adam-stochastic gradient descent approach. The learning rate is varied from 10^–3^ to 10^–8^ with an exponential decay in 2,000,000 steps for both the MLPs. The prefactors of energies and forces are taken as *P*_*e*_^start^ = 0.01, *P*_*f*_^start^ = 1000, *P*_*e*_^limit^ = 1, and *P*_*f*_^limit^ = 1, respectively. The validation of our MLPs is given in the Supporting Information.

In MD simulations, long-range interactions are typically handled using the K-space command in LAMMPS, which invokes a long-range electrostatics solver. In addition to this, we have developed a machine learning potential based on the Deep Potential Long-Range (DPLR) model^56^ for the low-pressure regime where the materials are predominantly polar. As shown in Figure S11, the strong agreement between the results from DPLR and DeepMD potentials (along with the agreement with the experimentally measured bulk modulus and thermal conductivity at ambient conditions for LiBr) supports the validity of our approach using the DeepMD framework.

### Green–Kubo Calculations of Thermal Conductivity

To calculate the lattice thermal conductivity, we employ the Green–Kubo (GK) formalism under the equilibrium MD framework, which uses our MLPs to describe the interatomic interactions. For the MD simulations, we used a time step of 1 fs and prescribed periodic boundary conditions in all three directions. We equilibrate our computational domains with a Nosé-Hoover thermostat and barostat to prescribe the temperature and pressure, respectively.^57^ The microcanonical ensemble is evoked to collect the heat current data to calculate the lattice thermal conductivity according to,

1 $$\kappa_{\alpha ,\beta ,\gamma }=\frac{1}{VK_{B}T^{2}}\int_{0}^{\infty }\left\langle J_{\alpha ,\beta ,\gamma }\left(0\right)J_{\alpha ,\beta ,\gamma }\left(t\right)\right\rangle \mathrm{dt}$$

where *K*_B_, *T*, *V*, and *t* are the Boltzmann constant, temperature, volume, and time, respectively, and ⟨*J*_α,β,γ_(0)*J*_α,β,γ_(*t*)⟩ is the heat current autocorrelation function (HCACF) along the three directions. Heat current data are sampled every 10 fs during the simulations and integrated for a total simulation time of 5 ns. More details regarding the convergence, sampling, and averaging of the thermal conductivity calculations are given in the Supporting Information.

### Spectral Energy Density (SED) Calculations

We perform MD simulations utilizing the SED formalism to asses the phonon mode-specific characteristics for our LiI and LiBr structures. In SED formalism, we Fourier transform the atomic velocities to get the average kinetic energy per unit cell which is expressed as a function of wave vector (**q**) and frequency (ω), which is given as,^45,58^

2 $$\Phi \left(q,\omega \right)=\frac{1}{4\pi \tau N_{T}}\overset{3}{\underset{\alpha }{\sum }}\overset{B}{\underset{b}{\sum }}m_{b}|\int_{0}^{\tau }\overset{N_{T}}{\underset{n_{x,y,z}}{\sum }}{\mathrm{u̇}}_{\alpha }\left(\begin{array}{c} n_{x,y,z}\\ b \end{array};t\right)\times {\exp\left[iq\cdot r\left(\begin{array}{c} n_{x,y,z}\\ 0 \end{array}\right)-i\omega t\right]dt|}^{2}$$

Here τ represents the total simulation time, α corresponds to the Cartesian direction, *n*_*x*,*y*,*z*_ represents a unit cell, *N*_*T*_ is the total number of unit cells in the crystal, *b* represents the atom label in a given unit cell, *B* is the atomic number in the unit cell, and *m*_*b*_ is the mass of atom *b* within the unit cell. The term *u̇*_α_ represents the velocity along the α direction at time *t*, and **r** corresponds to the equilibrium position of each unit cell.

Furthermore, the phonon lifetime is determined by manually identifying Lorentzian-shaped peaks and fitting each peak using the Lorentzian function, which is given as,^45^

3 $$\Phi \left(q,\omega \right)=\frac{I}{1+{\left[\left(\omega -\omega_{c}\right)/\gamma \right]}^{2}}$$

Here *I*, ω_*c*_, and γ represent the peak intensity, frequency at the peak center, and half-width at half-maximum, respectively.

### Spectral Heat Current Calculation

In order to understand the contribution of each vibrational mode to the total heat current we calculate the interfacial thermal current defined as,^59^

4 $$Q_{j\to i}=\int_{0}^{\infty }\frac{d\omega }{2\pi }q_{j\to i}\left(\omega \right)$$

where, ω is the angular frequency and *q*_*j*→*i*_(ω) is the interparticle spectral heat current which is given as,

5 $$q_{j\to i}\left(\omega \right)=2Re\left[{\mathrm{K̃}}_{ij}\left(\omega \right)\right]$$

Here, the spectral heat current between atoms *i* and *j* is related to the correlation time between the force, **F**_*i*,*j*_ and velocities, **v**_*i*_, **v**_*j*_ terms. *K̃*_*ij*_(ω) is the Fourier transform of the auxiliary correlation function defined as,

6 $$K_{ij}\left(t_{1}-t_{2}\right)=\frac{1}{2}\left\langle F_{i,j}\left(t_{1}\right)\cdot \left[v_{i}\left(t_{2}\right)+v_{j}\left(t_{2}\right)\right]\right\rangle$$

## Supplementary Material





## Notes

Validation of our machine learning based interatomic potentials; vibrational spectra and scattering phase space at elevated pressures; first-principles calculations of electronic and phononic structures; spectral energy density and spectral heat current calculations; equilibrium molecular dynamics simulations; pressure dependence in metal halides using Liebfried and Schlömann model (PDF)
Transparent Peer Review report available (PDF)
