# Supplementary material for: Achieving Exceptionally Enhanced Thermal Conductivity and Bulk Modulus in Polar Insulators Via Modification of Chemical Bonding
Source: J Phys Chem Lett. 2025 Aug 21;16(34):8850–60. doi: 10.1021/acs.jpclett.5c01476 (PMC12422541; doi:10.1021/acs.jpclett.5c01476)
Supplement: Supplementary file 1 [file jz5c01476_si_001.pdf]

Supporting Information : Achieving exceptionally enhanced thermal conductivity and bulk modulus in polar insulators via modification of chemical bonding

Niraj Bhatt, Sandip Thakur, Pravin Karna, and Ashutosh Giri*

Department of Mechanical Industrial and Systems Engineering, University of Rhode Island, Kingston, RI 02881, USA

E-mail: ashgiri@uri.edu

Table of Contents

S1. Validations of the machine learning-based interatomic potentials S2–S20

S2. Vibrational spectrum and scattering phase space at elevated pressures S20–S24

S3. First-principles calculations of electronic and phononic structures S24–S26

S4. Spectral energy density calculations and spectral heat current calculations S26–S31

S5. Equilibrium MD (EMD) Simulations S31–S23

S6. Pressure dependence in metal halides using Liebfried and Schlömann model . . . S24–S25

References S25–S27

S1. Validations of the machine learning-based interatomic potentials

The validation of the five distinct interatomic potentials designed to analyze the temperature as well as pressure dependence for LiBr and LiI structures at ambient and pressurized conditions are detailed below.

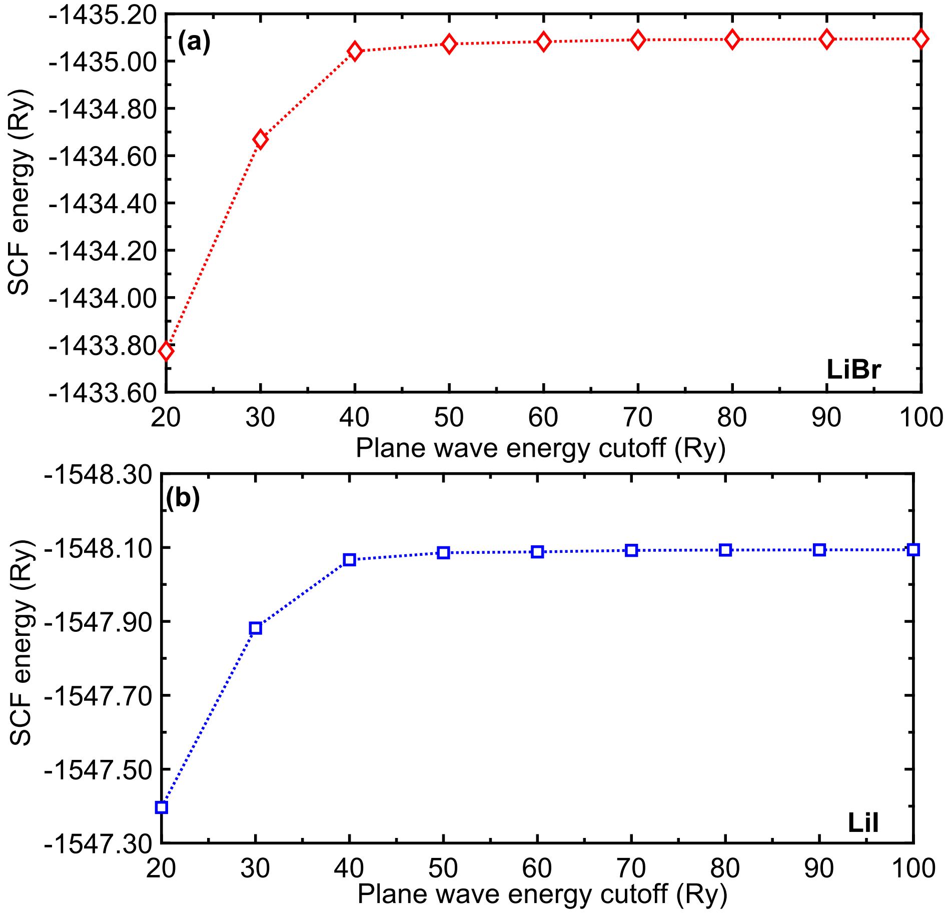

Figure S1: Variation of SCF energy as a function of plane wave energy cutoff for LiBr and LiI both.

First, we examine the choice of our energy cutoff for plane wave expansion used in all *ab initio* MD simulations for acquiring our training data. Figure S1 shows the results for the convergence of the self-consistent field (SCF) energy across the range of the plane wave energy cutoff from 20

Ry to 100 Ry for both the lithium halides. Using 60 Ry as plane wave energy cutoff as a suitable choice in all our *ab initio* MD simulations is ensured by the convergence of the SCF energies in the range of 60 to 100 Ry as shown in Fig. S1.

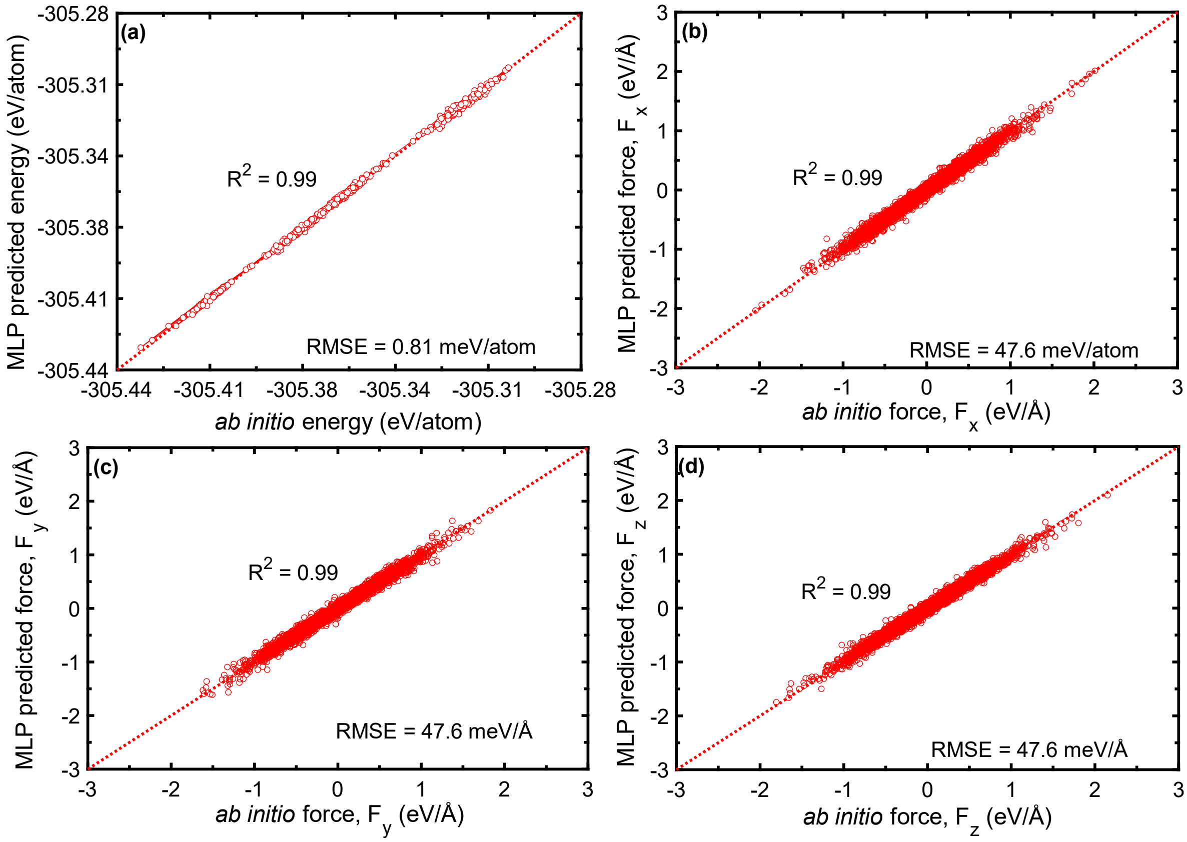

Figure S2: Comparison of (a) energies and (b-d) forces predicted with MLP and *ab initio* MD counterparts. The perfect x=y agreement of MLP-predicted versus *ab initio* energies and forces reflected in R^2 values close to unity and low RMSE ascertains the accuracy of our MLPs.

To evaluate the accuracy of the trained MLPs, two different evaluation metrics namely root-mean-square error (RMSE) and R-squared value (R^2) are calculated. To assess the accuracy of our MLPs, we compare the energies and forces obtained from our MLPs and our *ab initio* MD simulations. In fact, very low RMSE and R^2 values close to 1 validates our interatomic potentials and our training procedures for all of our trained MLPs. Figure S2 shows the validation plot for temperature-based potential for LiBr comparing the *ab initio* energies and forces along the three principal directions with our MLP-predicted energies and forces along the corresponding directions. Similarly, Fig. S3 shows the comparison between the *ab initio* energies and forces

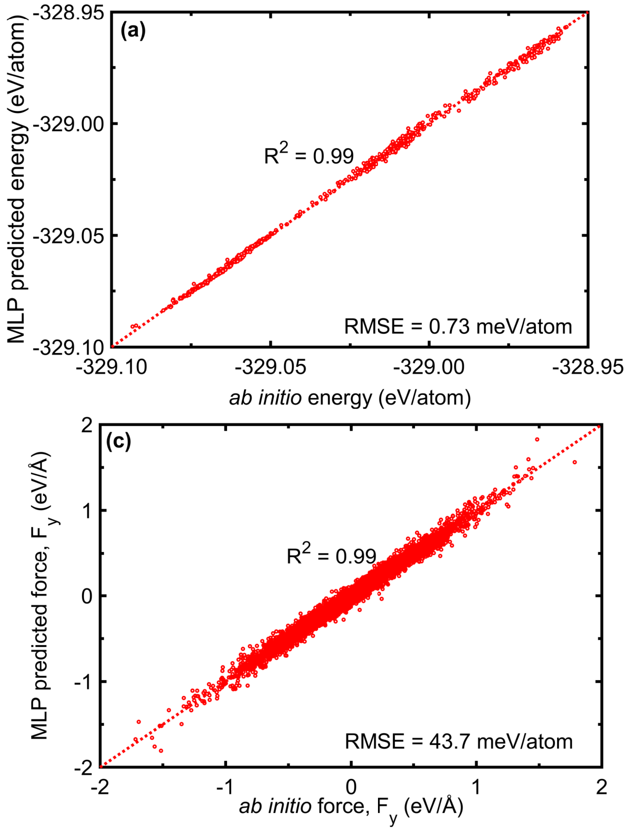

Figure S3: Comparison of (a) energies and (b-d) forces predicted with MLP and *ab initio* MD counterparts. The perfect x=y agreement of MLP-predicted versus *ab initio* energies and forces reflected in R^2 values close to unity and low RMSE ascertains the accuracy of our MLPs.

for LiI along the three principal directions with our MLP-predicted energies and forces along the corresponding directions for the temperature-based potential for LiI.

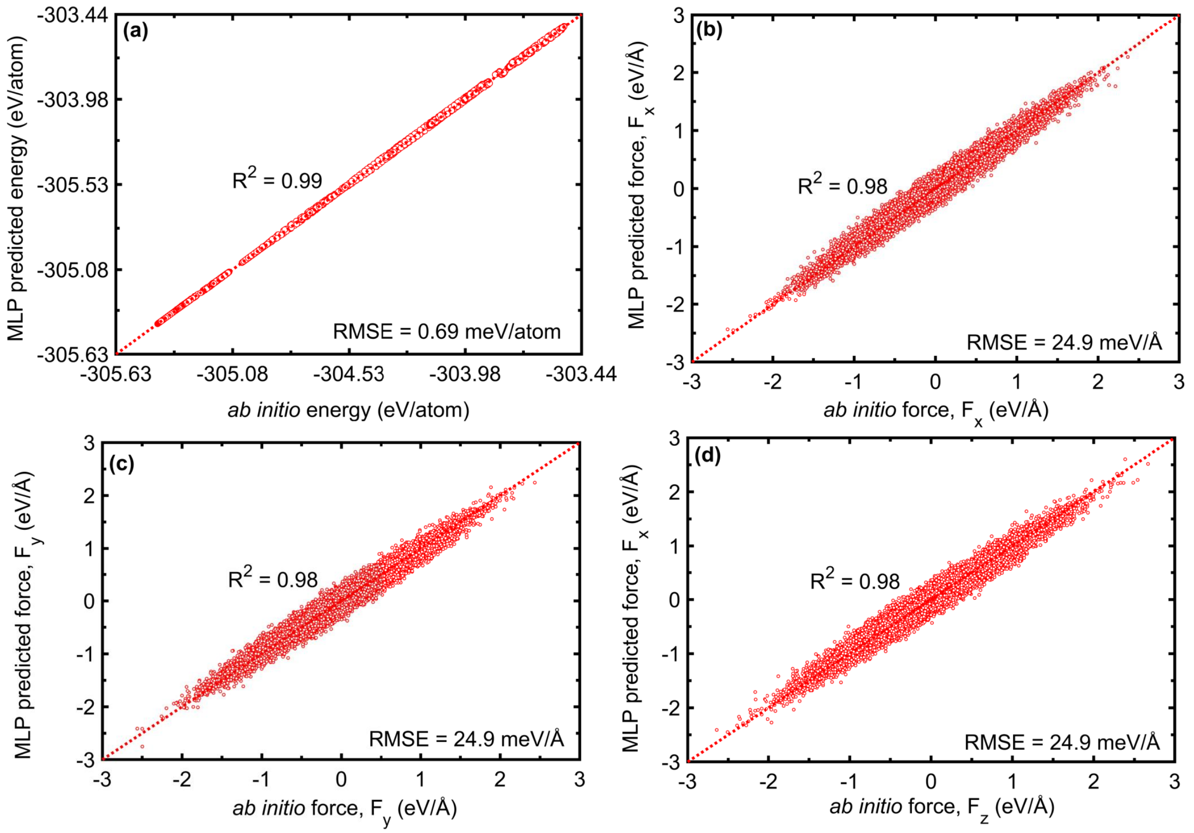

Figure S4: Validation plots comparing (a) energies and (b) forces given by our MLP with *ab initio* MD counterparts for LiBr as a function of pressure at ambient temperature. The perfect x=y agreement of MLP-predicted vs *ab initio* energies and forces reflected in R^2 value close to unity and low RMSE ascertains the accuracy of our MLP across the pressure range.

Similarly, the validation of interatomic potentials for LiBr and LiI for pressure dependence can be ascertained by the perfect agreement between our MLP predictions with the exact *ab initio* counterparts as evident from Fig. S4 and Fig. S5 respectively. Finally, Fig. S6 shows the parity plots for the energies and forces comparing our MLP predictions with the exact *ab initio* counterparts for temperature-based potential at elevated pressures (90 GPa) for LiI. The perfect $x = y$ agreement between the *ab initio* and MLP-predicted values indicate that the machine-learned potential is uniformly accurate over the entire temperature and pressure range considered in our work. The R^2 value close to unity as well as the low RMSE value in the parity plots reinforces the excellent

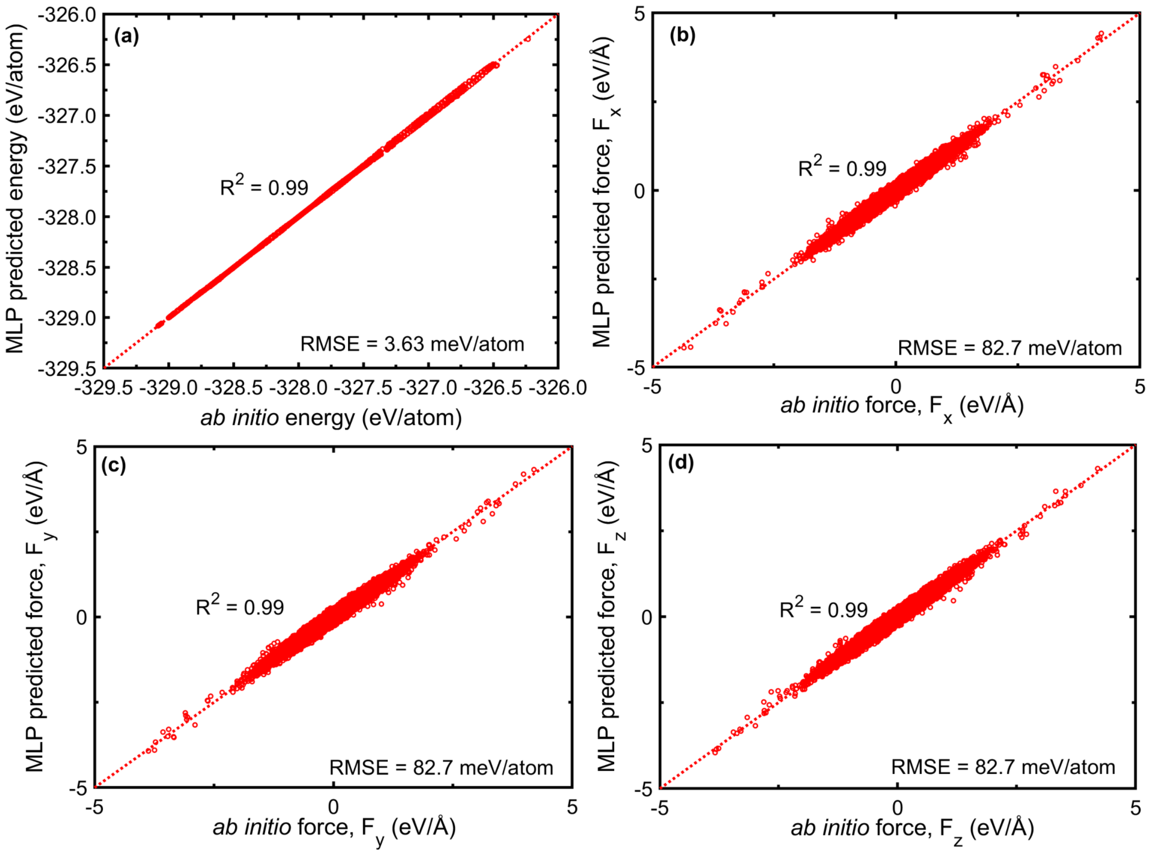

Figure S5: Validation plots comparing (a) energies and (b) forces provided by our machine learning potential (MLP) with *ab initio* molecular dynamics (MD) counterparts for LiI as a function of pressure at ambient temperature. The perfect agreement represented by the line $x = y$ in the MLP-predicted energies and forces compared to the *ab initio* results, indicated by an R^2 value close to one and a low RMSE, confirms the accuracy of our MLP across the pressure range.

quality of fits between our MLP-predicted as well as *ab initio* MD values.

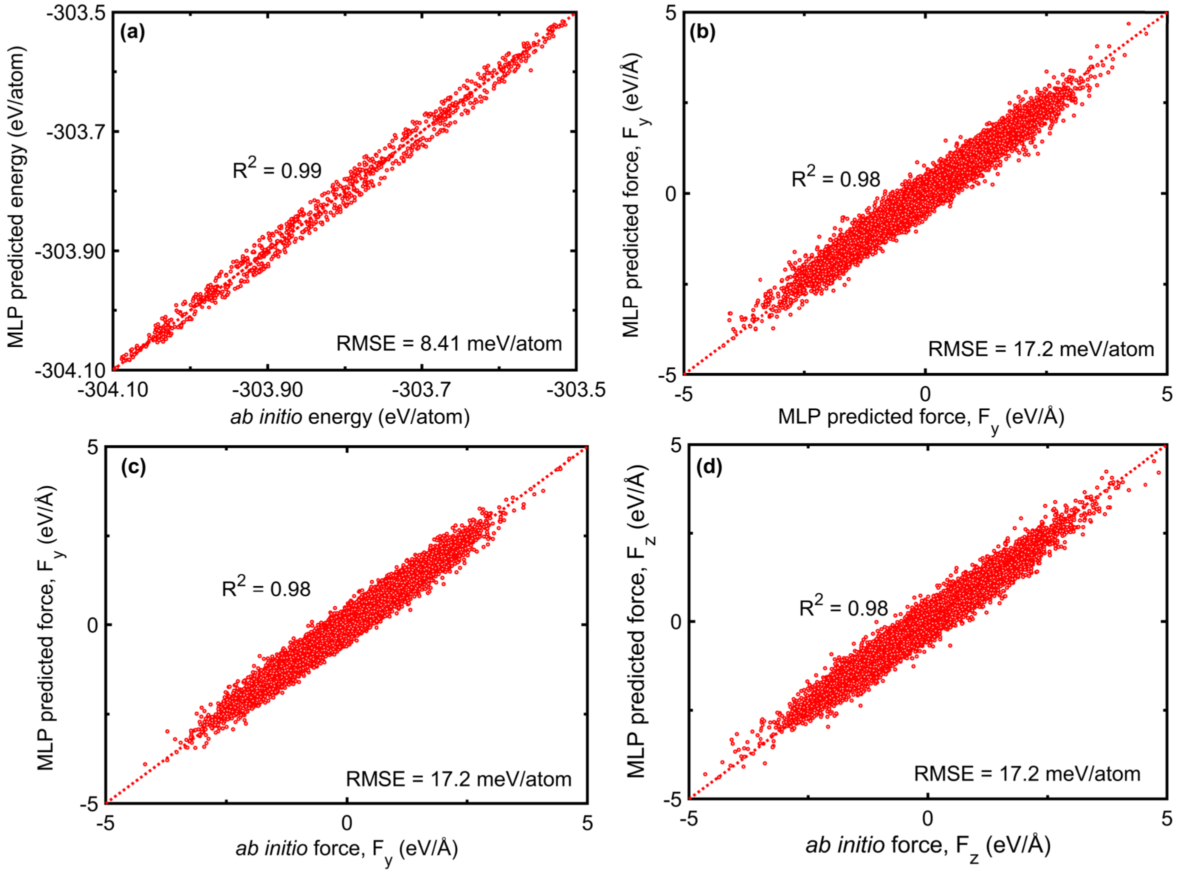

Figure S6: Validation plots comparing (a) energies and (b-d) forces given by our MLP with *ab initio* MD counterparts for LiBr as a function of temperature at 90 Gpa. The perfect x=y agreement of MLP-predicted vs *ab initio* energies and forces reflected in R^2 value close to unity and low RMSE ascertains the accuracy of our MLP over the entire temperature range at elevated pressures.

Additionally, our results as shown in Fig. S7 show that our MLP potential can very well replicate the mechanical response of our LiBr structures as a function of pressure as indicated by the perfect agreement between DFT-calculated and MLP-predicted densities and volumetric contractions.

We developed a new machine learning potential (MLP) for LiBr that incorporates long-range electrostatic interactions using the Deep Potential Long-Range (DPLR) model. This model combines the short-range accuracy of DeepMD with explicit long-range interactions calculated via a Gaussian charge model known as the Deep Wannier (DW) framework. The system was constructed using a 2×2×2 supercell of LiBr (64 atoms) at 300 K and 0 GPa. The DPLR framework utilizes two

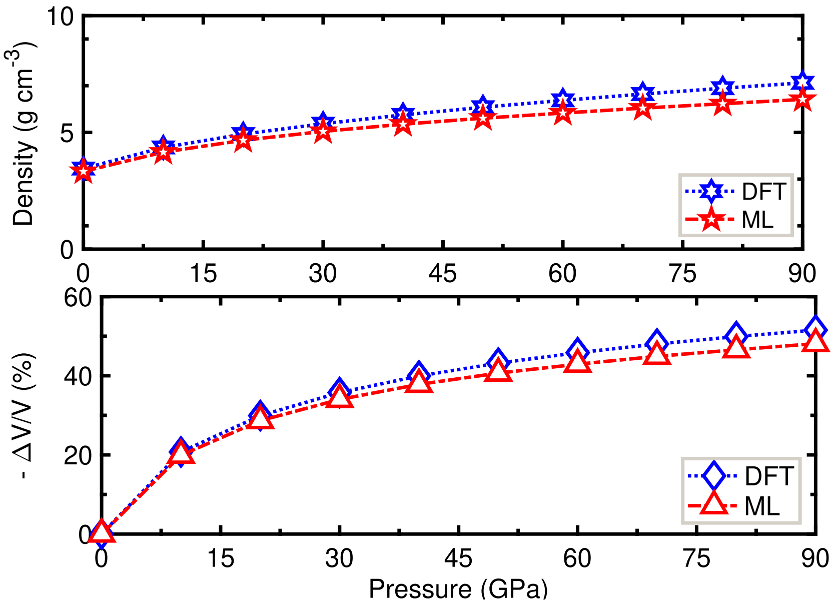

Figure S7: Comparison of our MLP-predicted density and relative volume changes with DFT-calculated ones as a function of pressure for LiBr. Our MLP can replicate its' relative volume and density changes as compared to DFT-calculated changes as a function of pressure.

neural networks: the DeepMD network for short-range forces and a pretrained DW network to predict long-range electrostatics using Ewald summation. Wannier centers, representing charge centroids, are predicted from atomic environments using the DW model, trained to reproduce centers derived from DFT-calculated maximally localized Wannier functions (MLWFs). The DPLR model was then jointly optimized to fit DFT energies, forces, and stresses. The total potential energy surface (PES) thus captures both short- and long-range interactions.[S1] The long-range energy is modeled as the interaction of spherical Gaussian charges, where the charges are split between fixed ion cores and the dynamically computed valence electron centers (i.e., Wannier centroids).[S2,S3] The Wannier centers were calculated using wannier90[S4] interfaced with Quantum ESPRESSO,[S5] employing SCF and NSCF DFT calculations with PBE functionals. These MLWFs transform delocalized Kohn-Sham orbitals into localized charge densities. A total of 192 Wannier centers were obtained for LiBr, forming the dataset for training. A final DPLR model was trained over 5 million steps with an exponentially decaying learning rate (10^{-3} to 10^{-8}), and a dynamic loss function initializing the pre-factors of energies and forces as $P_e^{\rm start} = 0.01,\ P_f^{\rm start} = 1000,\ P_e^{\rm limit} = 1$, and $P_f^{\rm limit} = 1$, respectively. Ewald summation (β =0.1) was used to compute electrostatic contribu-

tions, and automatic batching handled the training dataset efficiently. The resulting model achieves high fidelity in reproducing both short- and long-range interactions critical for accurately describing ionic dynamics in LiBr. Training and validation results confirm excellent agreement between predicted and reference DFT forces and energies as evidenced from Fig. S8.

We calculated the thermal conductivity of LiBr at 300 K and low pressures of 0, 5, and 10 GPa through non-equilibrium molecular dynamics (NEMD) simulations (see Fig. S10) utilizing a DPLR-trained potential to assess the effect of long-range electrostatics in thermal conductivity predictions in lithium halides. This method creates a steady heat flow by applying thermal reservoirs at opposing ends of the simulation box, producing a linear temperature gradient across the transport axis (x-axis in our case). Thermal conductivity values are determined from the temperature profile slope in the middle region via Fourier's law. Our NEMD simulations yield system-size-converged thermal conductivity values across all pressure conditions. For example, our NEMD simulations yield a system-size-converged thermal conductivity of roughly 1.2 W m^{-1} K^{-1} showing excellent agreement with our DeepMD-prediction (1.14 W m^{-1} K^{-1}) as well as prior theoretical as well as experimental works.[S6,S7] We selected NEMD over equilibrium molecular dynamics (EMD) due to current incompatibilities in implementing DPLR model in LAMMPS for Green-Kubo equilibrium methods. Notably, our DPLR-based NEMD results demonstrate strong agreement with DeepMD model predictions at all three pressure points, validating the reliability of our MLPs in thermal conductivity predictions across the pressure range.

To further reinforce the validity of our interatomic potentials, we evaluated the mechanical response of our LiBr and LiI structures through bulk moduli calculations and compared it with experimental and DFT results. The bulk modulus (K) of a material quantifies its resistance to compression under pressure. It is defined as the proportion of a small increase in pressure to the corresponding relative reduction in volume and is given by,[S11]

$$K = -V\left(\frac{\partial P}{\partial V}\right)_T \tag{S1}$$

where V is the volume, P is pressure and T is temperature (held constant at 300 K for all our

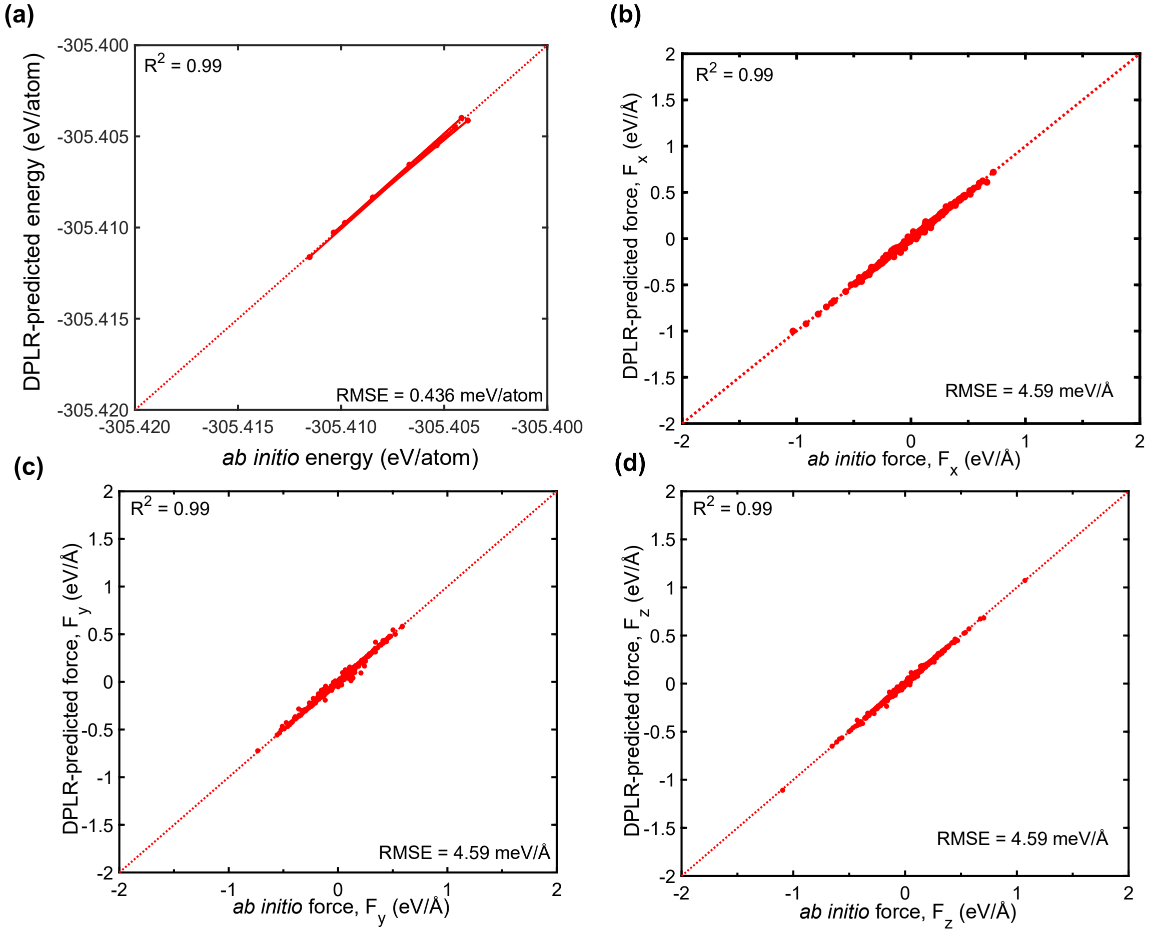

Figure S8: Validation plots comparing (a) energies and (b-d) forces given by our DPLR model with *ab initio* MD counterparts for LiBr at 0 Gpa. The perfect x=y agreement of DPLR-predicted vs *ab initio* energies and forces reflected in R^2 value close to unity and low RMSE ascertains the accuracy of our DPLR model at ambient conditions.

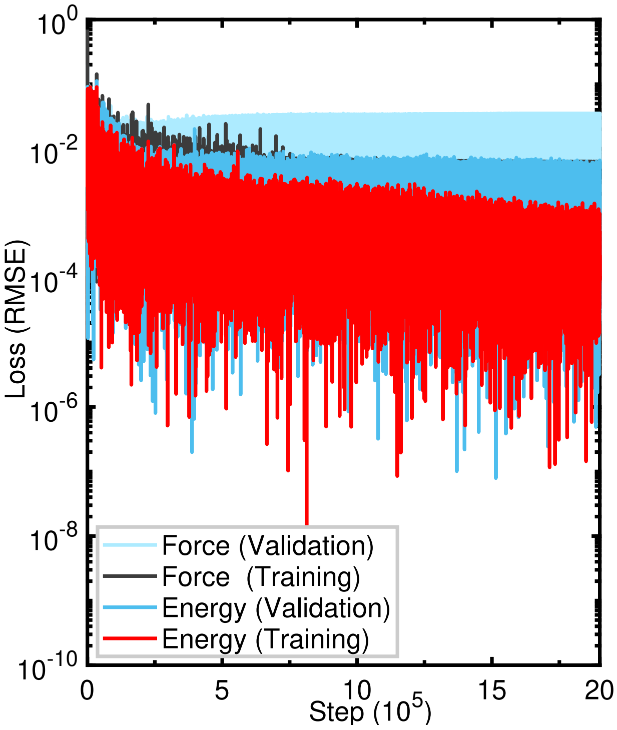

Figure S9: Evolution of RMSE scores during training for the Deep Potential Long-Range (DPLR) model, demonstrating high accuracy as demonstrated by consistent convergence of learning curves for energy and force components. Errors diminish to very low magnitudes, confirming the accuracy of our trained DPLR model.

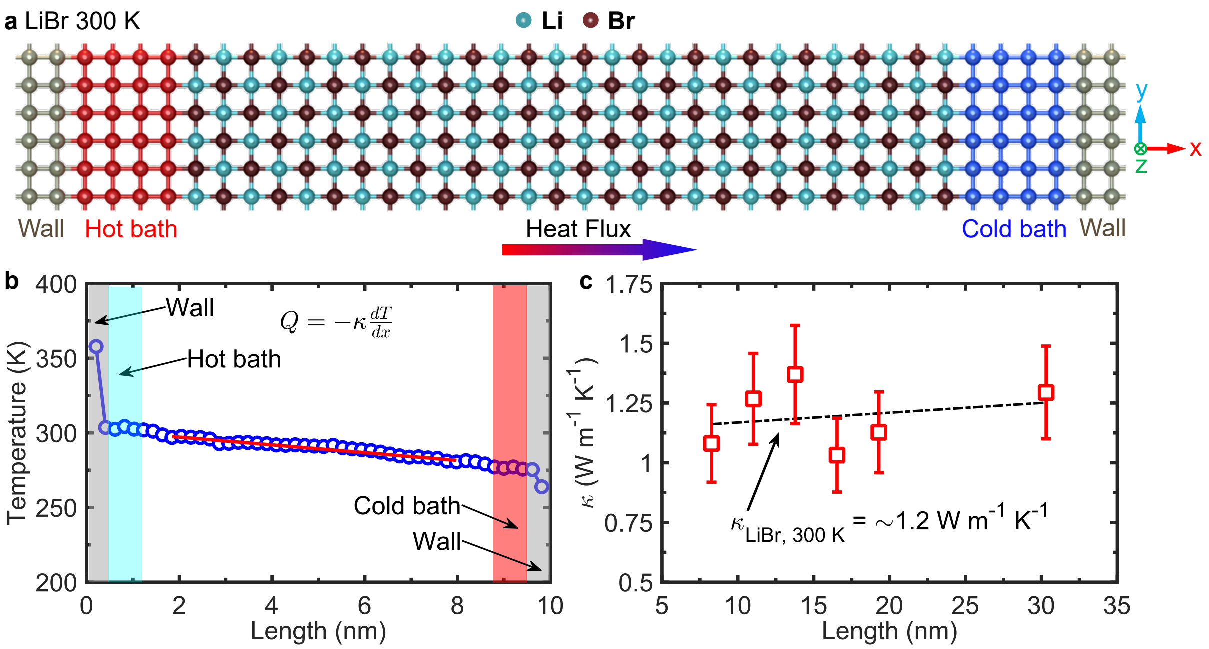

Figure S10: DPLR-based Non-equilibrium molecular dynamics (NEMD) simulations to find thermal conductivity of LiBr at 300 K. (a) Simulation setup for LiBr between hot (red) and cold (blue) baths creating heat flux along x-direction. (b) Linear temperature gradient across the simulation domain allowing thermal conductivity extraction via Fourier's law. (c) Variation of thermal conductivity as a function of domain size yielding convergence to κ = 1.2 W m^{-1} K^{-1} showing excellent agreement with our DeepMD-prediction without incorporating long range electrostatics (1.14 W m^{-1} K^{-1}).

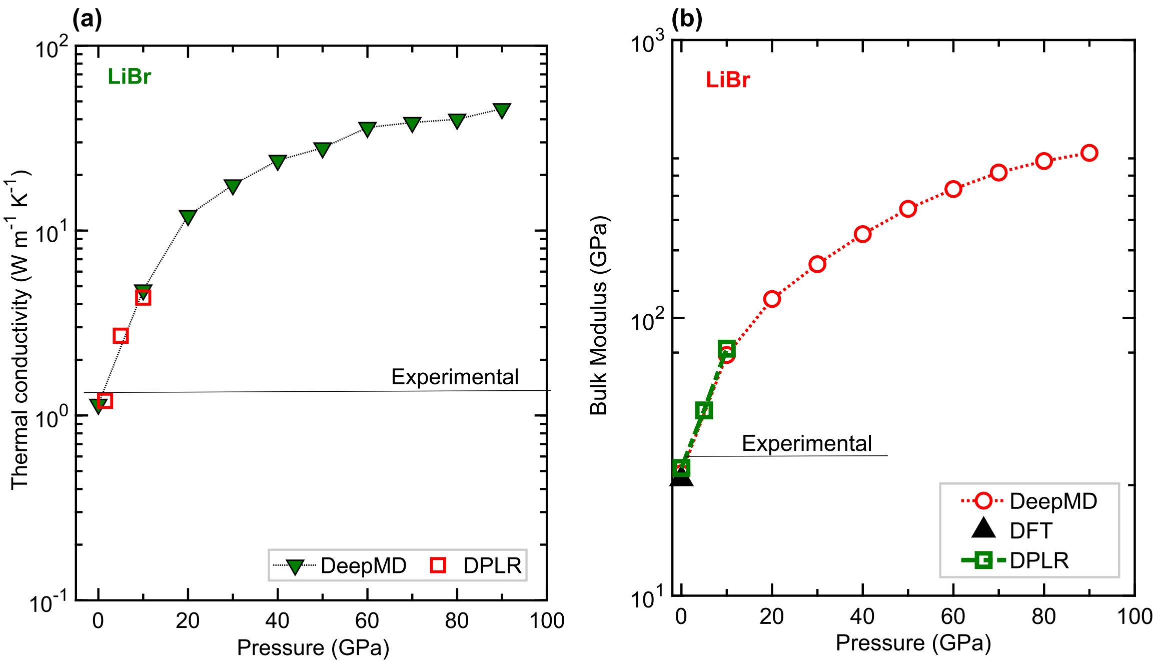

Figure S11: We developed a new potential using the Deep Potential Long-Range (DPLR) framework, which explicitly includes long-range electrostatic interactions for the low-pressure regime. At low pressures, where the system exhibits polar and ionic characteristics, we observe strong consistency in the predictions of (a) thermal conductivity and (b) bulk modulus between the DPLR and DeepMD models. Additionally, our computational results are supported by experimental data obtained under ambient conditions.[S7–S10]

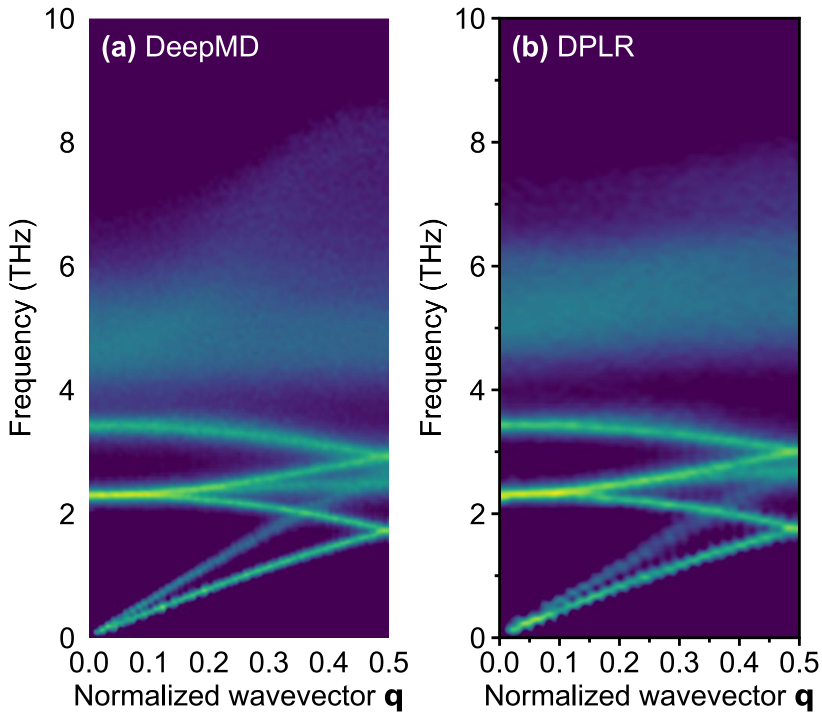

Figure S12: Calculated phonon spectral energy densities (SEDs) for LiBr at 300 K using DeepMD and DPLR models. We do not observe any significant differences in the SEDs with the inclusion of long-range electrostatic interactions.

bulk moduli calculations). The negative sign denotes the inverse relationship betweeen the volume and pressure of the system and the derivative of pressure with respect to volume of the system is denoted by $\frac{\partial P}{\partial V}$. This can be obtained by the fitting the pressure-volume(P-V) data and obtaining the corresponding slopes. To calculate the bulk moduli for LiBr and LiI systems as a function of pressure, we track the volumetric response of the system as the pressure of the system increases using DFT calculations as well as our MLP-MD simulations. The bulk modulus at a particular pressure is directly computed from the slope of the P-V curve multiplied by the equilibrium volume at that particular pressure.

Figure S14a and Fig. S14b shows the P-V relationships for LiBr and LiI as a function of pressure calculated using our MLP-MD. The bulk modulus shows a consistent increase with pressure for the corresponding lithium halides, as illustrated in the inset figures. All of our bulk modulus calculations show convergence within the bounds of statistical uncertainties, irrespective of the selected domain size as shown in Fig. S15.

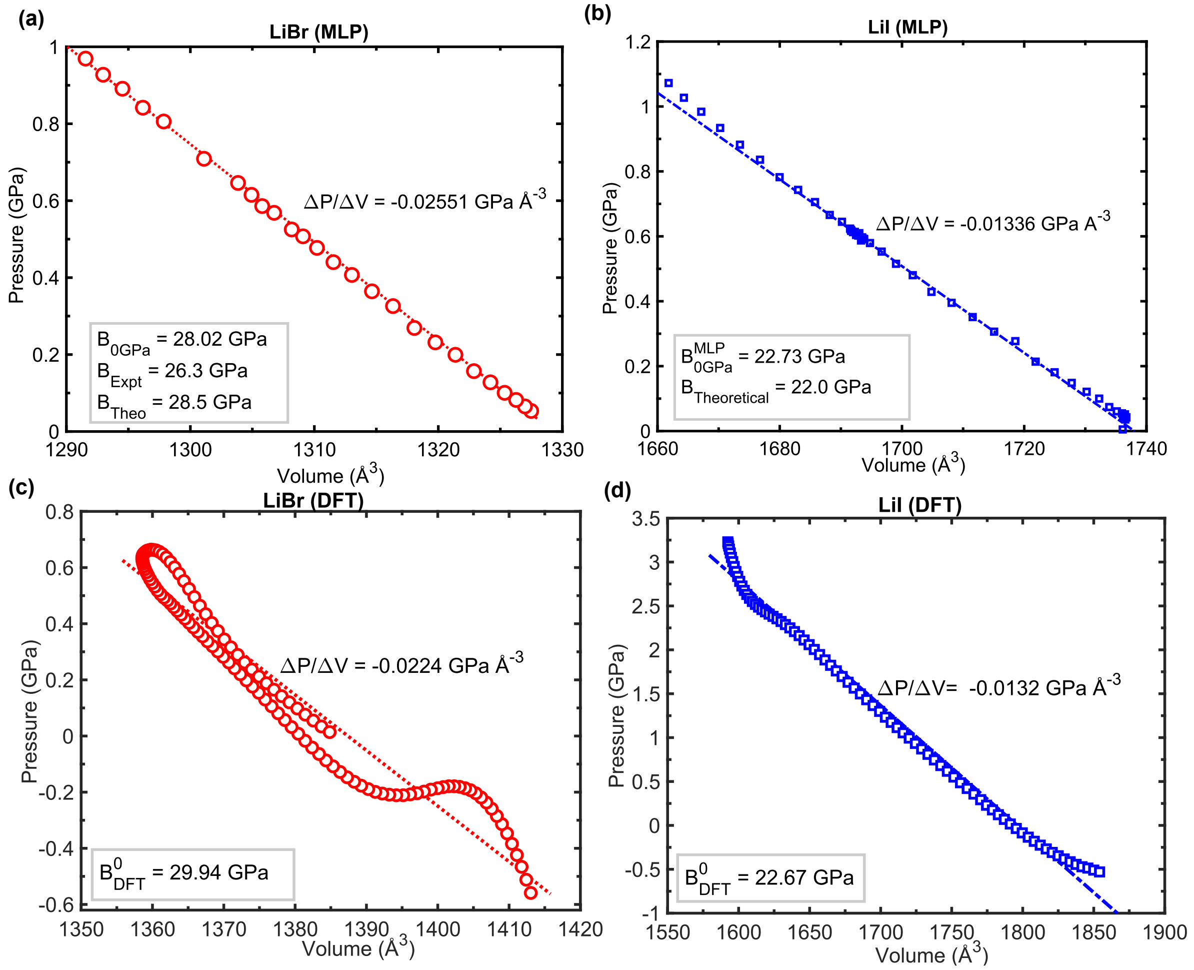

Figure S13: Validation plot comparing the bulk moduli of lithium halides using our MLP with that using DFT-based calculations. (a) LiBr (MLP) and (b) LiI (MLP) show the calculated bulk modulus using our MLP-MD calculations at 0 GPa. (c) LiBr (DFT) and (d) LiI (DFT) show the DFT-calculated bulk moduli results at 0 GPa. The excellent agreement between our MLP-based predictions and DFT calculations validates our MLP for both the lithium hallides.

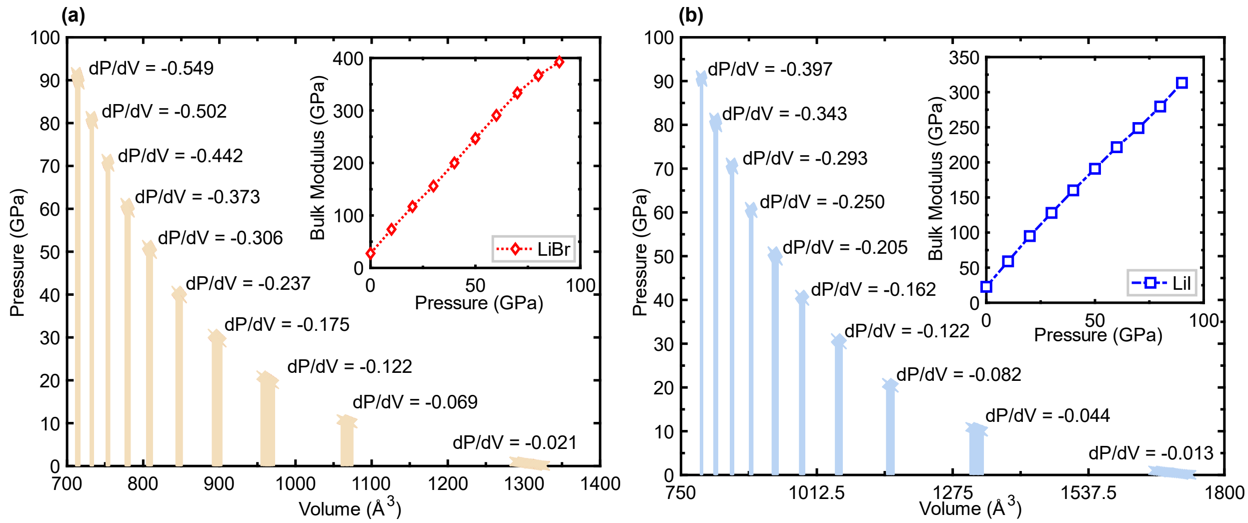

Figure S14: Pressure-Volume (P-V) relationships for (a) LiBr and (b) LiI using our MLP-MD simulations. (inset) The monotonic increment of the bulk modulus as a function of pressure for the respective lithium halides.

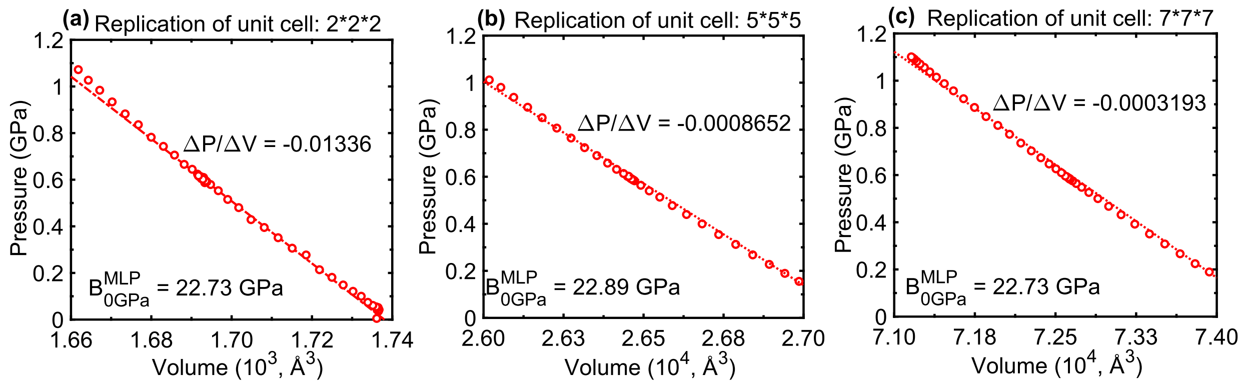

Figure S15: MLP-MD predicted bulk modulus for LiI as a function of computational domian size. We notice that all of our bulk modulus calculations demonstrate convergence within the limits of statistical uncertainties, regardless of the chosen domain size.

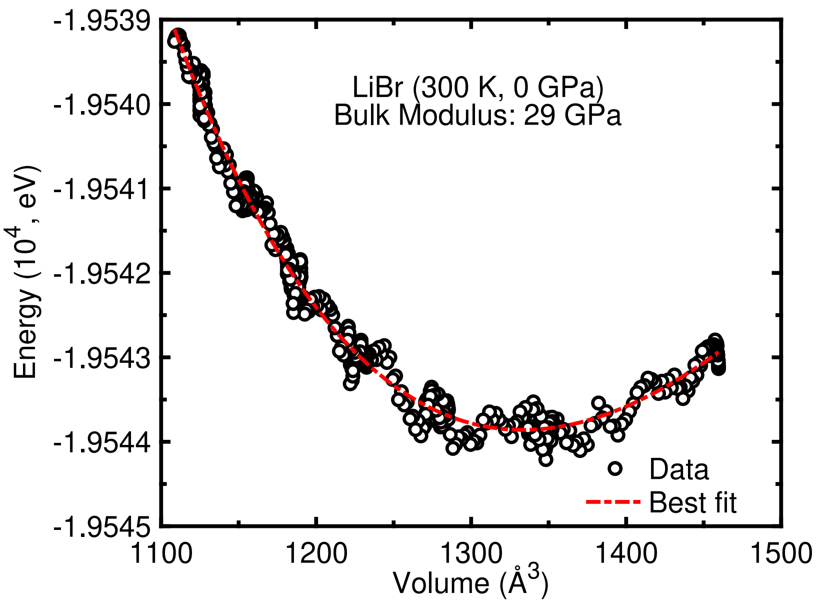

Figure S16: Calculated bulk modulus for LiBr at 0 GPa using the machine learned interatomic potential. The dashed lines represent fits to the Birch-Murnaghan equation of state (EOS).[S12–S14]

Our machine learning potential (MLP) demonstrates strong predictive capability for both the mechanical and thermal properties of lithium halides, yielding values in close quantitative agreement with prior experimental studies, as shown in Fig. S11. For LiBr, the MLP predicts a bulk modulus of 28.02 GPa, which is remarkably consistent with DFT (29.94 GPa), experimental results (28.5 GPa), and earlier theoretical reports (26.3 GPa) using Voigt-Reuss-Hill method. Likewise, for LiI, the predicted bulk modulus of 22.73 GPa aligns well with DFT (22.67 GPa) and theoretical estimates (22.0 GPa).[S8–S10]

Beyond elasticity, the MLP captures thermal transport trends with similar accuracy, predicting a thermal conductivity of 1.14 W m^{-1} K^{-1} for LiBr. This value is in reasonable agreement with the experimental measurement of 1.8 W m^{-1} K^{-1} and matches the theoretical value of 1.31 W m^{-1} K^{-1} within the reported 15–20% statistical uncertainties.[S6,S7] The close agreement across DFT, experimental, and theoretical benchmarks confirms the robustness of our MLP and supports its application for extended simulations and predictive studies of lithium halides.

Our workflow leverages the Green–Kubo (GK) approach for thermal conductivity, a well-established method widely used across material classes from simple to complex systems.[S15–S17]

Prior comparisons between the GK method and the HNEMD technique have demonstrated that GK yields accurate results within statistical error margins.[S18] Moreover, GK has been extensively combined with machine-learned interatomic potentials to estimate lattice thermal conductivity in diverse materials.[S19–S21] Several benchmark studies have confirmed that DeepMD-based predictions closely track both DFT–BTE calculations and experimental observations across a wide range of systems.[S22,S23] In our prior works, we also successfully applied DeepMD to model lattice thermal transport (with the GK formalism) in different metals, achieving strong agreement with DFT and experimental values.[S24,S25] Another notable work from *Li et al.* accurately reproduces the thermal conductivity in various silicon phases, where DeepMD results align well with both experimental and ab initio data.[S26]

We also calculated bulk modulus for LiBr at ambient using equation of state approach as shown in Fig. S16 to reinforce the robustness of our P-V approach which is a fundamentally standard technique.[S11,S27,S28] The bulk modulus is computed using the equation of state (EOS) approach,[S12–S14] implemented through a multi-step workflow in LAMMPS.[S29] This procedure involves sequential energy minimizations and systematic volume perturbations to evaluate the mechanical response of the system. Initially, the structure is relaxed using a two-stage energy minimization protocol that combines the conjugate gradient (CG)[S30] and fast inertial relaxation engine (FIRE)[S31] algorithms to ensure convergence on the potential energy surface. Following structural relaxation, a series of volume perturbations are applied under NPT ensemble to generate an energy–volume (E–V) dataset. The resulting E–V data are then fitted to the Murnaghan equation of state.[S12,S14] The agreement of bulk modulus using our standard P-V approach with experimental, DFT-based, theoretical as well as EOS approach further reinforces the robustness of our approach.

We also examined the temperature dependence of the bulk modulus in LiBr under both ambient and extreme pressures, comparing our findings with those of other alkali halides and diamond. Notably, at 90 GPa, the bulk modulus shows a near-constant behavior as temperature varies, demonstrating remarkable stability under high pressure. In contrast, at ambient pressure, we observe a subtle yet consistent linear decline, mirroring trends seen in other halides as evidenced in Fig. S17.

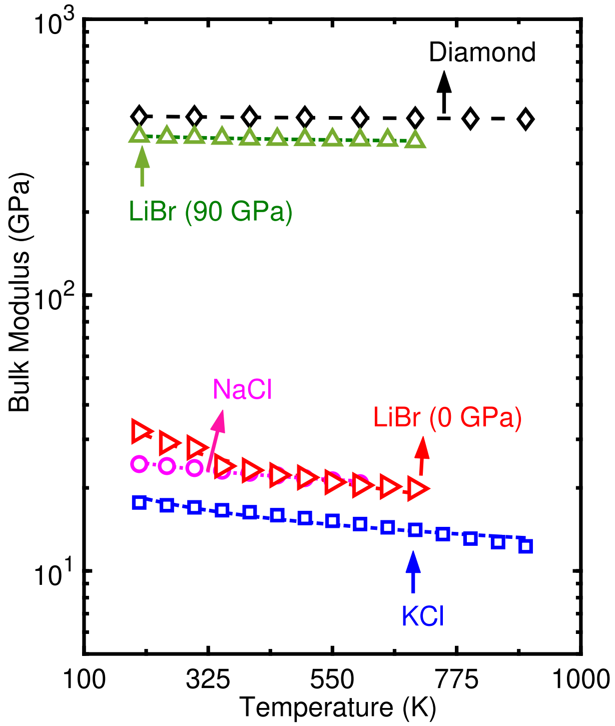

Figure S17: Temperature dependence of the bulk modulus in LiBr examined at both ambient and extreme pressures, and compared with other alkali halides and diamond.[S32] At 90 GPa, we observe that the bulk modulus remains nearly constant with temperature, while at ambient pressure, there is a very weak, monotonic linear decrease, which is similar to the trend seen in other halides.[S33]

S2. Vibrational spectrum and scattering phase space at elevated pressures

We calculated the phase spaces for phonon scattering and the MLP-based phonon dispersions of lithium halide structures under varying pressures by using the ALAMODE package.[S34] We observe shift in the vibrational spectrum for both the lithium halides as shown in Fig. S18. Noteworthy is the clear wide gap between the accoustic and optical modes in vibrational spectrum suggesting significantly reduced accoustic-optical interactions at elevated pressures.

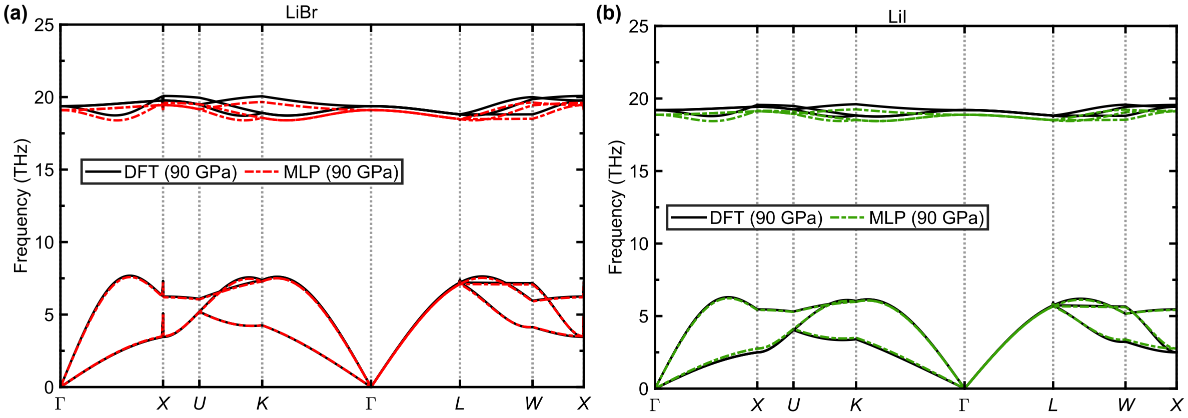

Figure S18: Comparison of MLP-based phonon dispersions with their DFT counterparts at 90 Gpa for LiBr and LiI. The vibrational spectrum are comparable for both the halides spanning as high as 20 THz. We observe shift in the vibrational spectrum at elevated pressures, indicating phonon hardening.

Similarly, we observe increase in the slopes of the phonon branches with rising pressure, as depicted in Fig. 2 of the main manuscript, resulting in remarkable enhancement in group velocities for both lithium halides. We observe phonon hardening as indicated by the shift of the vibrational spectrum to higher frequencies. Figure S19 shows the group velocities for the LiI at ambient, 50 and 90 GPa for the acoustic and optical modes separately (refer Fig. 4a in manuscript for similar trend of group velocities for LiBr). We notice a monotonic increase in the group velocities of the acoustic modes for both the lithium halides, however, the group velocities of the optical modes are observed to decrease consistently with increasing pressure for both the lithium halides.

Additionally, our MLP-MD based calculations for mean square displacement (MSD) as a func-

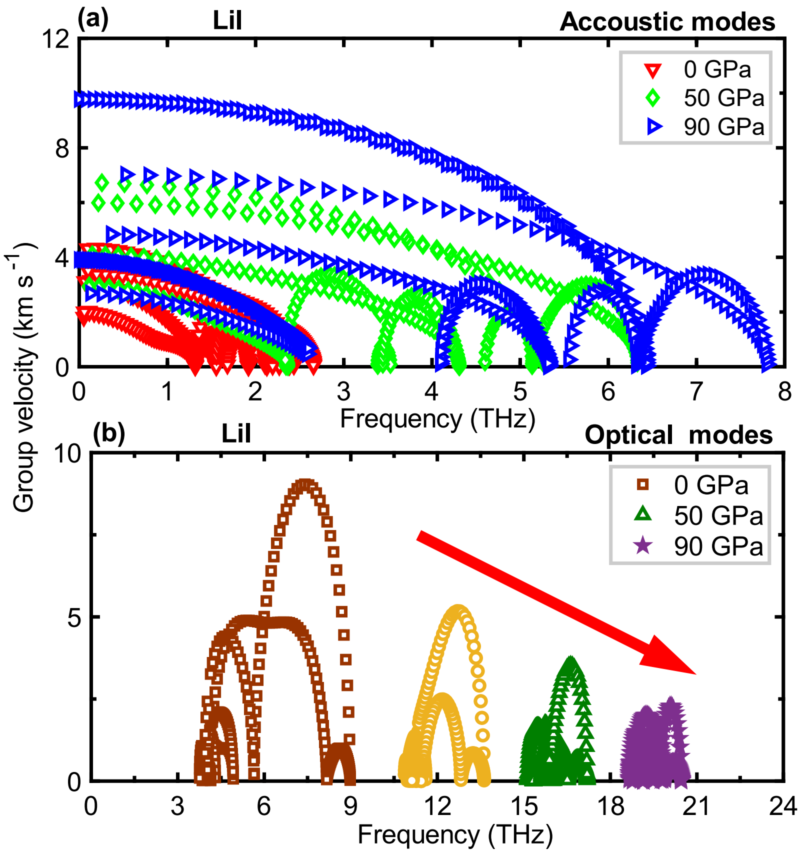

Figure S19: Group velocities calculated separately for the (a) acoustic and (b) optical modes at ambient, 50 and 90 GPa for LiI. The group velocities increase monotonically for acoustic modes as a function of pressure. In contrary, the group velocities for the optical modes are seen to decrease monotonically with pressure.

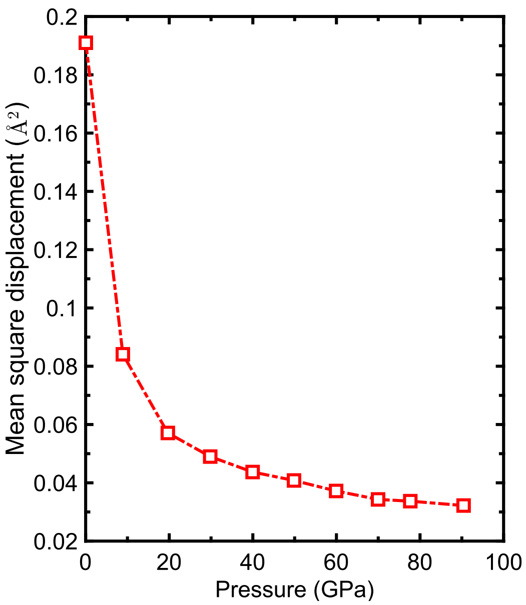

Figure S20: Mean square displacement (MSD) calculated using our MLP-MD simulations.

tion of pressure show that MSD decreases monotonically with pressure due to lattice-stiffening (see Fig. S20).

Similarly, we also investigate the influence of pressure upon the total scattering phase space at ambient and 90 GPa as shown in Fig. S21 for both the lithium halides. It is seen that the scattering phase space decreases significantly as the pressure is increased from ambient to 90 GPa. We observe an order of magnitude decrement in the scattering phase space for intermediate (from ∼2.5 to ∼7.5 THz) as well as higher frequencies ($>$ 15 THz) for both the lithium halides. The significant gap introduced between the heat carrying acoustic modes and high frequency optical modes in the phonon spectra leads to the reduced acoustic-optical interactions and decreases the overoll phonon-phonon scattering rate as evident from decreased scattering phase space as shown in Fig. S21, resulting in increased lattice thermal conductivity at elevated pressures.

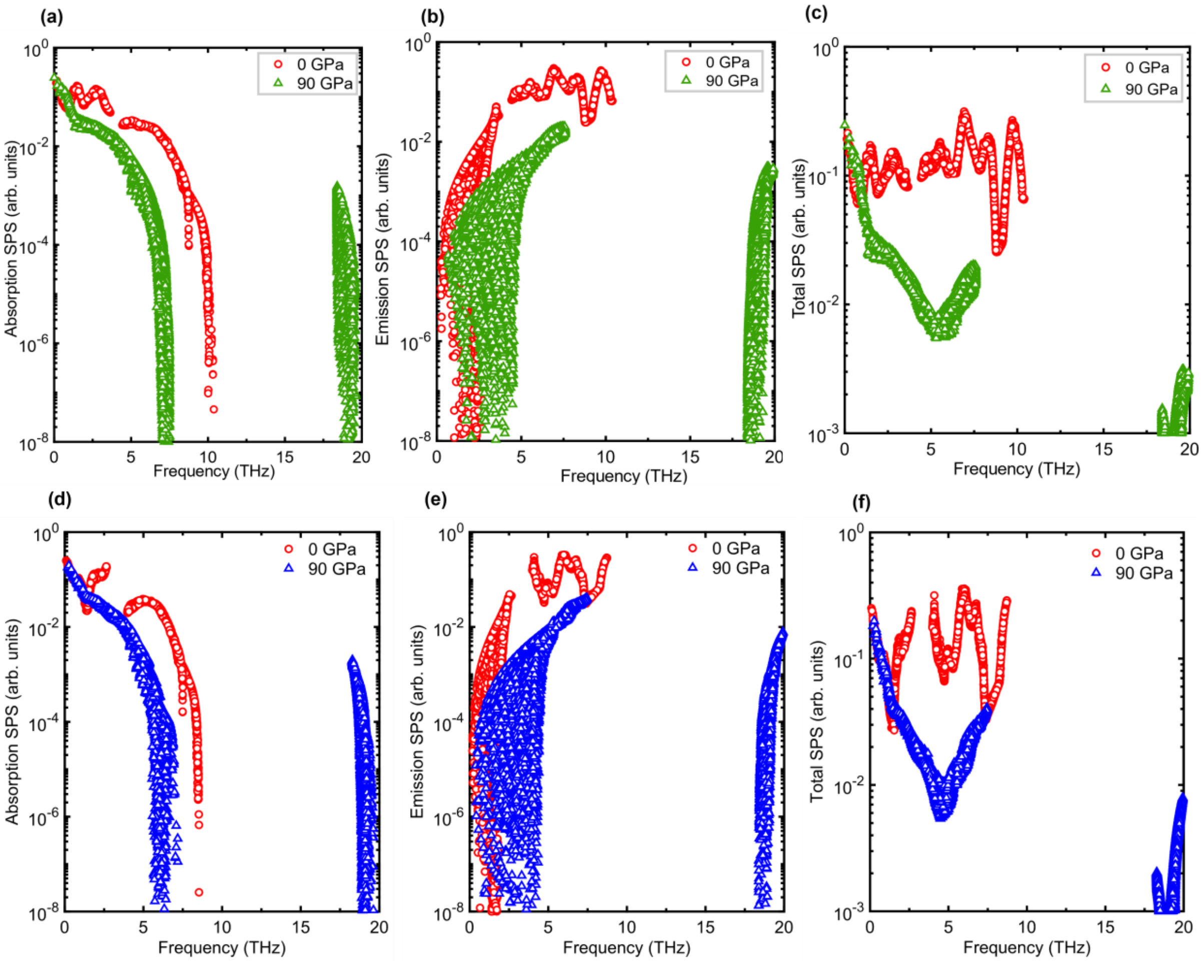

Figure S21: Comparison of the absorption, emission and total scattering phase spaces at ambient and pressurized conditions for (a-c) LiBr and (d-f) LiI. We can clearly observe an order of magnitude reduction in the total scattering phase space for intermediate (from ∼2.5 to ∼7.5 THz) as well as higher frequencies (> 15 THz) for both of the lithium hallides. This significant reduction in the total scattering phase space leads to an exceptional amplification of lattice thermal conductivity in both the lithium halides at pressurized conditions due to decreased phonon-phonon scattering rates at elevated pressures.

S3. First-principles calculations of electronic and phononic structures

The electronic and phononic properties in both the ambient and increased pressures are computed using density functional theory (DFT) as implemented in Quantum Espresso.[S5] The DFT calculations utilize scalar relativistic norm-conserving pseudopotentials within the generalized gradient approximation (GGA) framework, specifically employing the Perdew-Burke-Ernzerhof (PBE)[S35] functional for the exchange-correlation potential. A plane-wave cut-off energy of 150 Ry is set for these computations. To achieve convergence in structural and electronic properties at the ground state within the PBE framework, a $20 \times 20 \times 20$ k-point mesh is employed for Brillouin zone integration. At higher pressures, convergence is further tested using an enhanced k-point mesh of $50 \times 50 \times 50$ and plane wave cut-off energy of 200 Ry. The separation of longitudinal optical (LO) and transverse optical (TO) phonons in the phonon dispersion relation is obtained through the incorporation of Born effective charges and the dielectric tensor in phonon calculations.

We calculate the electron localization function (ELF) for gallium nitride (GaN), silicon carbide (SiC) and cubic boron nitride (c-BN). For GaN, we present the ELF at 35 GPa, which is below its phase transition pressure of 37 GPa[S41], showing that the bonding environment remains largely unchanged compared to its ground state. Similarly, we visualize the ELF for SiC at 75 GPa, a pressure lower than its phase transition threshold of 80 GPa.[S42] Under this pressure, slight modifications in the bonding environment are observed. Finally, we analyze the ELF for c-BN, where the impact of pressure is minimal, as c-BN remains stable up to approximately 1000 GPa.[S43]

We investigate the chemical bonding in LiBr and LiI using the Crystal Orbital Hamilton Population (COHP) method implemented in the LOBSTER code.[S44,S45] COHP allows the decomposition of the electronic band energy into individual atomic or orbital interactions, classifying them as bonding (positive), anti-bonding (negative), or non-bonding (zero).[S44,S45] Our calculations reveal that as pressure increases, the bonding interactions become stronger, indicating enhanced covalent character. This quantitatively supports our observations of electron density localization between

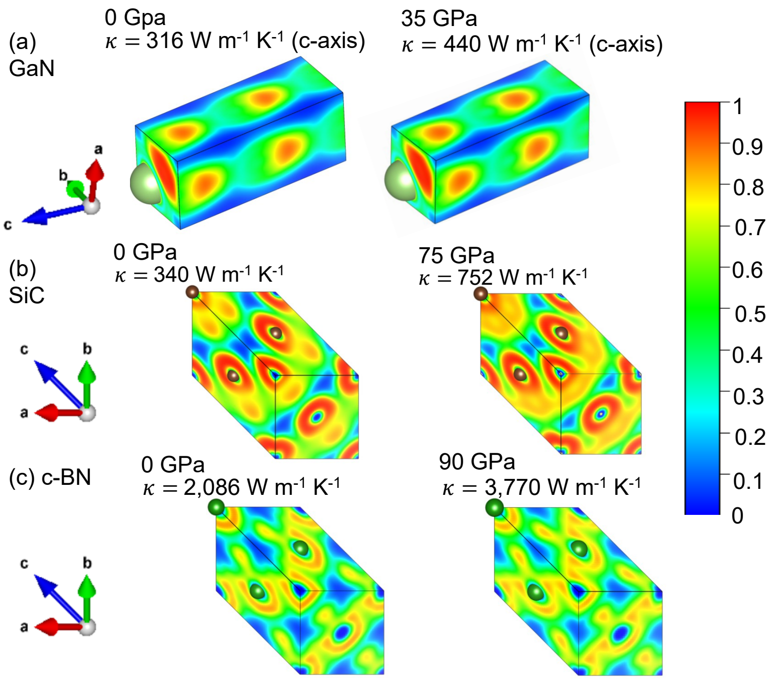

Figure S22: The Electron Localization Function (ELF) for (a) GaN, (b) SiC, and (c) c-BN is analyzed at both ambient and elevated pressures along with corresponding thermal conductivities. The thermal conductivity for GaN (along c-axis) increases from 316 W m^{-1} K^{-1} at ambient to 440 W m^{-1} K^{-1} at 35 GPa.[S36] Similarly, the thermal conductivity for SiC increases from 340 W m^{-1} K^{-1} at ambient to 752 W m^{-1} K^{-1} at 75 GPa pressure as estimated using Liebfried and Schlömann (LS) equation.[S37–S39] The thermal conductivity for c-BN also increases from 2,086 W m^{-1} K^{-1} at ambient to 3,770 W m^{-1} K^{-1} at 90 GPa.[S40] The bonding environment for GaN remains relatively stable, while slight changes are observed for SiC. However, no noticeable changes are evident for c-BN.

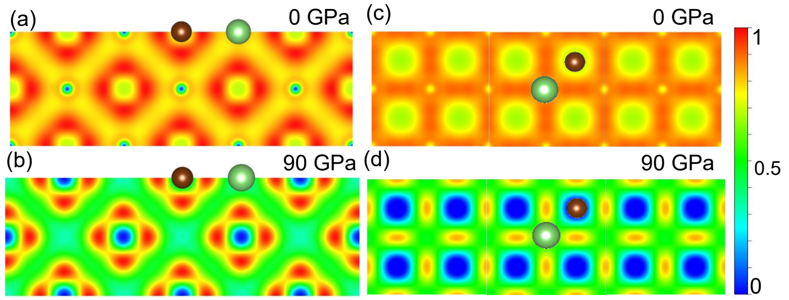

Figure S23: The charge density difference between a hypothetical crystal for lithium bromide that is put together from superposing the charge densities of free atoms and the charge density calculated for LiBr unit cells. The visualization planes are same as shown in the main manuscript for LiI (see Figs. 4g-4j). A directionality in the bonding is observed at higher pressures, indicating a more covalent nature of the bonds, but not as significant as LiI.

the lithium atoms and the halide anions, leading a greater covalent bonding character at higher pressures.

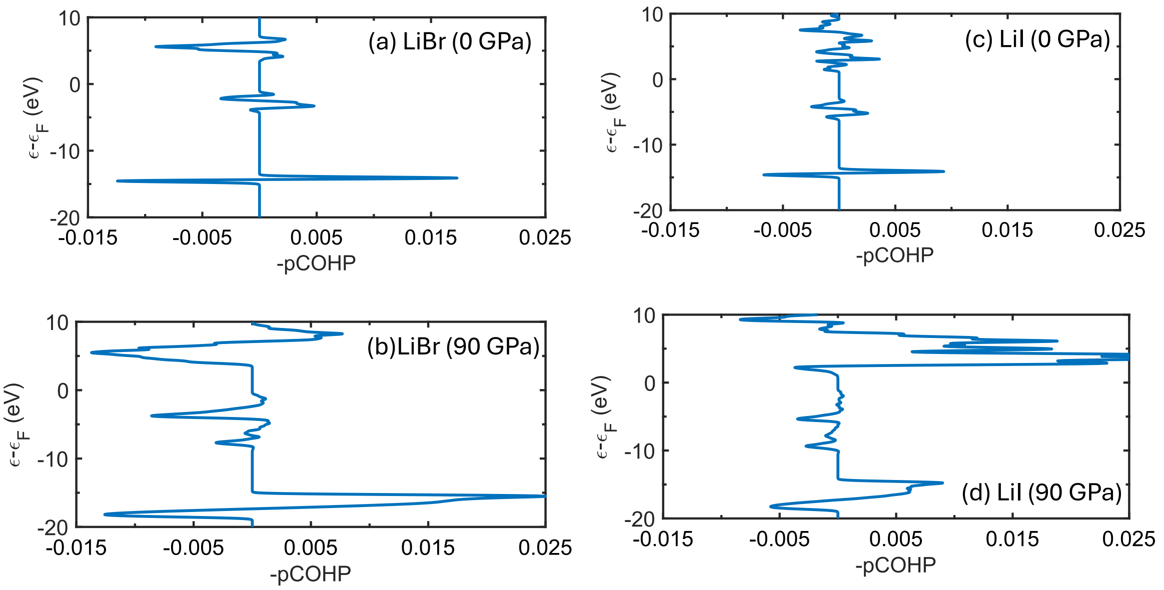

Figure S24: Crystal orbital Hamiltonian Population (COHP) for LiBr and LiI at 0 and 90 GPa. The COHP calculation shows that the bonding characteristic increases with increase in pressure.

S4. Spectral Energy Density (SED) Calculations and spectral heat current calculations

Our SED calculations for LiI as shown in Fig. S25 constitute SED profiles at ambient and pressurized conditions (see Fig 5a and 5c for LiBr). The decrease in broadening of the SED profiles are observed for both the halides at elevated pressures resulting in increased phonon lifetimes at higher pressures. We have carried out additional calculations of SEDs at higher temperatures at ambient and elevated pressures. As is clear from the Fig. S26, at higher temperatures the SEDs are broadened even more and the system becomes more anharmonic with temperature, thus leading to higher scattering of phonons and lowered thermal conductivity, which is more pronounced for the pressurized case as seen in Fig. S27. Similarly, a comparison of normalized spectral thermal conductivity for LiBr using the BTE framework can be observed in Fig. S28.

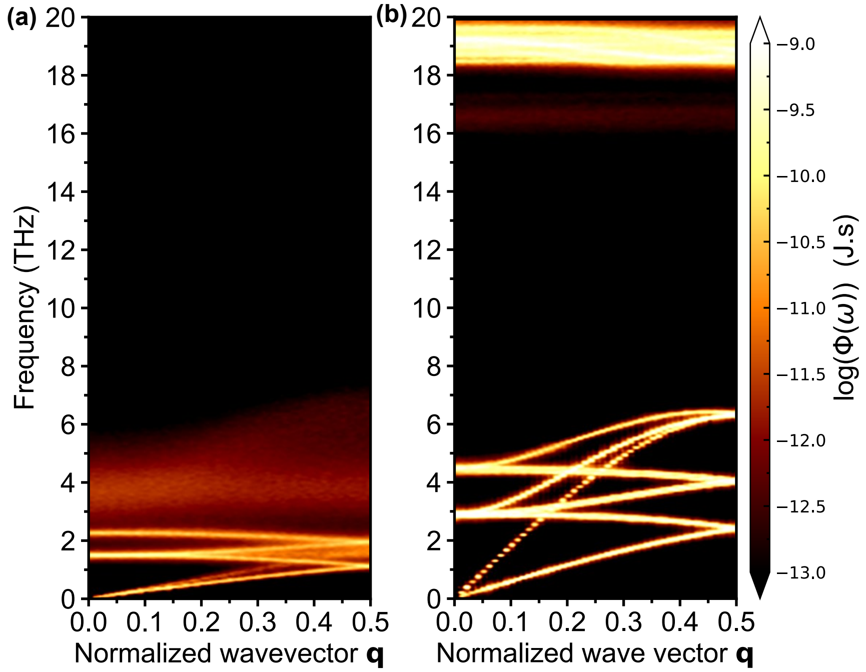

Figure S25: Spectral energy density calculations for LiI at (a) ambient and (b) at elevated pressure of 90 GPa. The anharmonic effects are clearly evident for higher frequency vibrational modes as indicated by relative broadening of the SED profiles and an increase in the contrast of the shading.

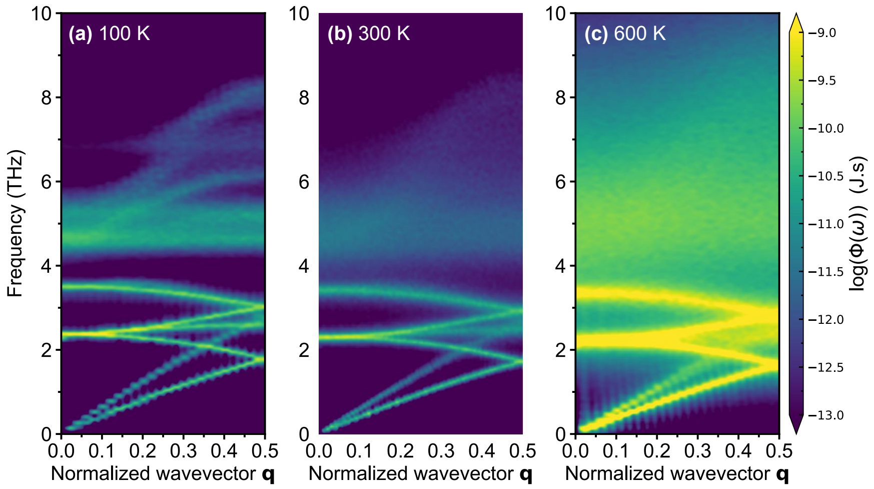

Figure S26: Calculated phonon spectral energy densities (SEDs) for LiBr at 0 GPa for (a) 100 K, (b) 300 K, and (c) 600 K. The progressive broadening of the SED peaks with increasing temperature indicates enhanced anharmonicity, stronger phonon-phonon scattering, and reduced phonon lifetimes resulting in lower thermal conductivity.

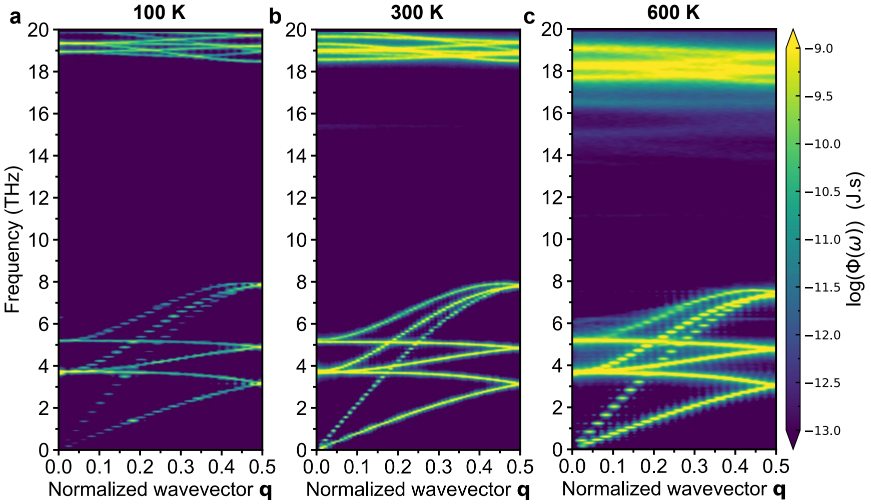

Figure S27: Calculated phonon spectral energy densities (SEDs) for LiBr at 90 GPa for (a) 100 K, (b) 300 K, and (c) 600 K. The progressive broadening of the SED peaks with increasing temperature indicates enhanced anharmonicity, stronger phonon-phonon scattering, and reduced phonon lifetimes resulting in lower thermal conductivity.

Our spectrally resolved normalized thermal conductivity calculations, illustrated in Fig. S28, reveal a fascinating insight: optical phonons play a minimal role in contributing to the total thermal conductivity, whether under ambient conditions or at elevated pressure. In striking contrast, it is the acoustic phonons that predominantly control heat transport in both scenarios, highlighting their predominance in dictating the thermal conductivity of the system. Our calculations for phononic lifetimes for LiBr using SED-based calculations reveal that increase in pressure enhances the phononic lifetimes as evident from Fig. S29.

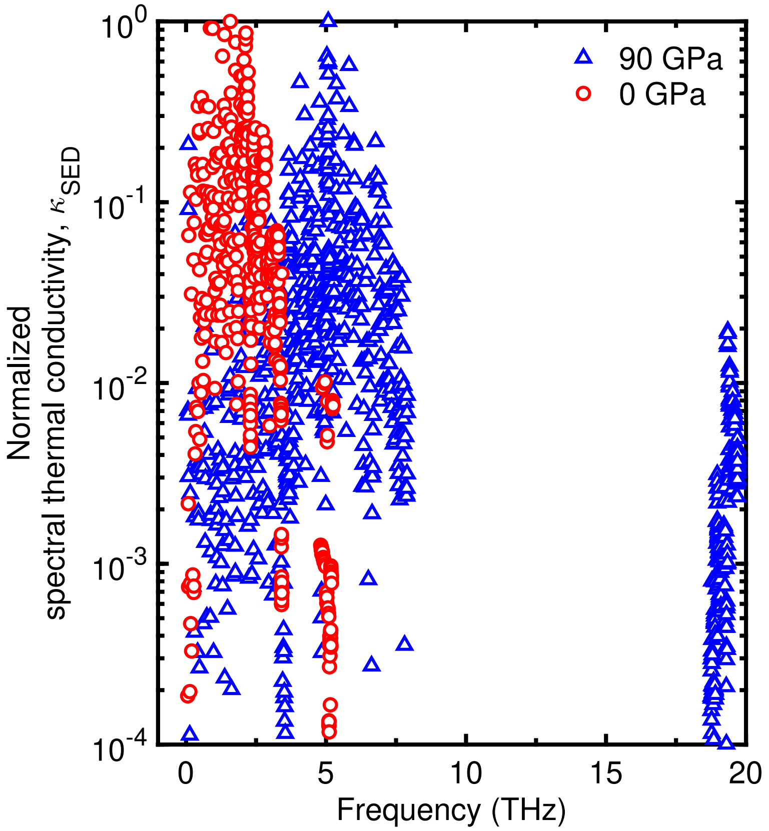

Figure S28: Comparison of normalized spectral thermal conductivity for LiBr calculated using the BTE framework with phonon lifetimes estimated from SED calculations at 0 and 90 GPa. The negligible contribution of optical phonons to the total thermal conductivity highlights that acoustic phonons predominantly govern heat transport at both pressures.

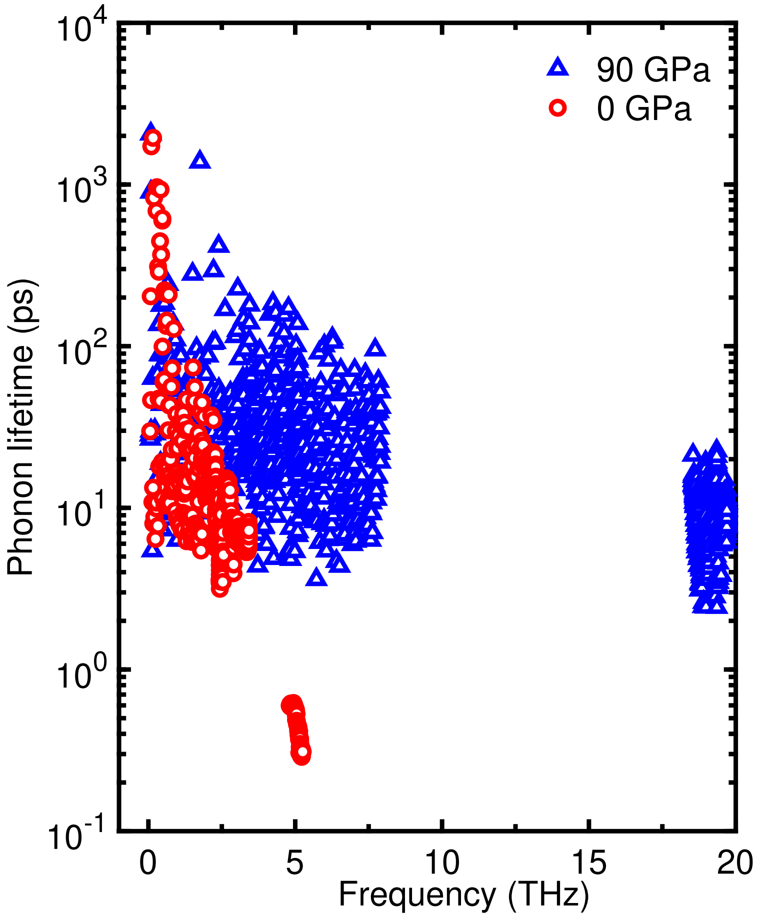

Figure S29: Comparison of phononic lifetimes for LiBr at ambient and elevated pressure from our SED-based calculations. We notice an increase in phononic lifetimes as a function of pressure across the frequency regime which is consistent with increase in phononic conductivity with pressure.

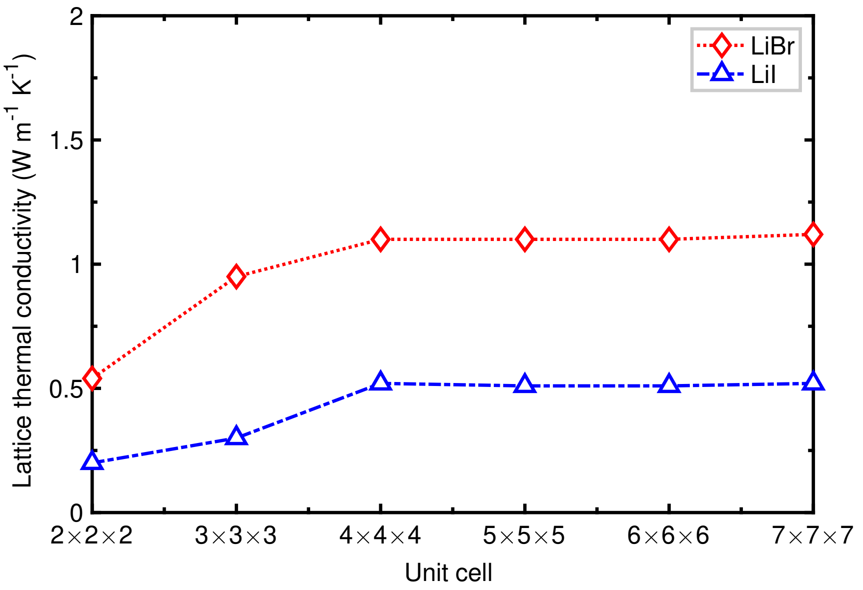

Figure S30: (a) Green-Kubo predicted lattice thermal conductivity of LiBr and LiI as a function of computational domain size. The convergence of thermal conductivity for computational domain sizes beyond 4×4×4 ensures that our choice of the domain size does not influence our GK predictions for both LiBr as well as LiI structures.

S5. Equilibrium MD (EMD) Simulations

For calculating the lattice thermal conductivity of our LiBr and LiI structures at various temperatures and pressures, we use the well-known Green-Kubo formalism. The total correlation time period ranging from 50 ps to 200 ps is used to achieve converged heat current autocorrelation function (HCACF) for all our temperature and pressure-based simulations. We use a sampling interval of 10 fs while collecting the data to calculate the HCACF across the entire range of temperatures as well as pressures. Finally, the heat current autocorrelation function is integrated to get the converged thermal conductivity for LiBr and LiI structures.

To ensure that our MLP-MD results are unaffected by the choice of our computational domian size for LiBr and LiI, we conduct a series of MD simulations by systematically varying the computational domain sizes and observe convergence within statistical uncertainties for the domain size of 4×4×4 that we use in our MLP-MD simulations as shown in Fig. S30. The convergence of

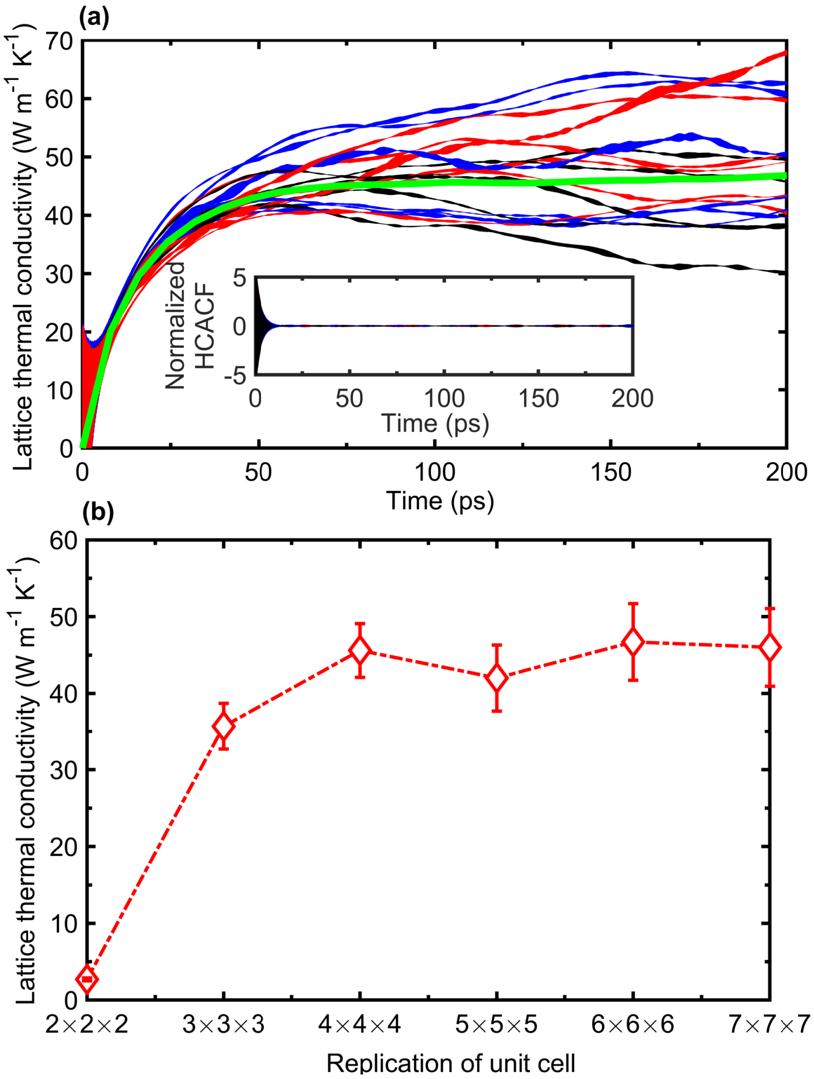

Figure S31: (a) Green-Kubo predicted thermal conductivity of LiBr at 90 GPa (300 K) in all directions as a function of the integration time. (inset) A fully decayed normalized heat current autocorrelation function (HCACF) as a function of the integration time. (b) Lattice thermal conductivity of LiBr at 90 GPa as a function of computational domain size.

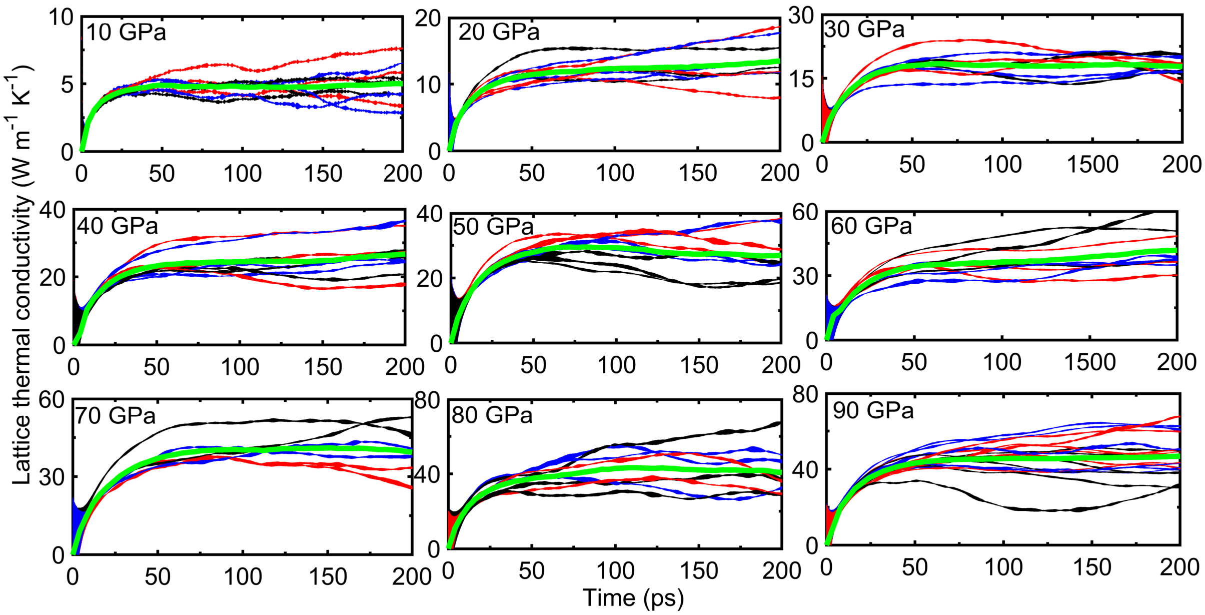

Figure S32: Green-Kubo predicted thermal conductivities for average 5 different runs as a function of the integration time at different pressure for LiBr. The thick green lines denote the average predicted thermal conductivities for each pressure. We average out the thermal conductivity by taking mean thermal conductivity from 50 ps to 200 ps.

thermal conductivity within uncertainties for computational domain sizes ensures that our choice of the domain size of 4×4×4 does not influence our GK predictions for both LiBr as well as LiI structures. Figure S31a shows the converged thermal conductivity for LiBr at 90 GPa and ambient temperature. We observe convergence within statistical uncertainties for the domain size of 4×4×4 that we use in our MLP-MD simulations at elevated pressures for LiBr as shown in Fig. S31b. To predict the green-kubo based lattice thermal conductivities at various pressures for LiBr and LiI, we perform 5 independent simulations at each pressure ranging from ambient to 90 Gpa as shown in Fig. S32 and Fig. S33, respectively. The thermal conductivity reported is estimated by taking the mean value of all the simulations. The uncertainties quantified by standard deviation range less than 15% in all our simulations.

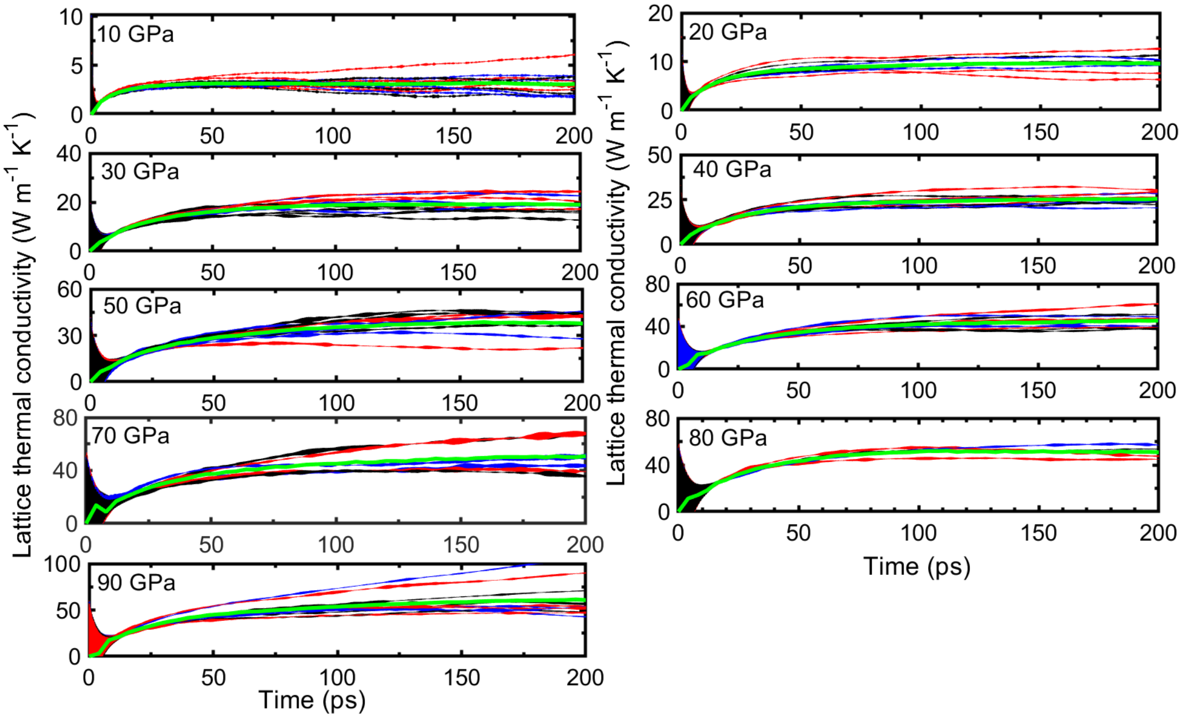

Figure S33: Green-Kubo predicted thermal conductivities for average 5 different runs as a function of the integration time at different pressures for LiI. The thick green lines denote the average predicted thermal conductivities for each pressure. We average out the thermal conductivity by taking mean thermal conductivity from 50 ps to 200 ps.

S6. Pressure dependence in metal halides using Liebfried and Schlömann model

The pressure dependence in non-metallic solids is well-explained by theory of Liebfried and Schlömann (LS),[S37] which predicts that thermal conductivity (κ) increases with pressure (dκ/dP >0) assuming dκ/dP $>$ 0 being a universal feature of materials. Although, the LS equation is grounded in a detailed theoretical framework of phonon transport,[S46] it has not been extensively tested under range of pressures that significantly impact a crystal's debye frequency, density, and elastic constants. Additionally, as the LS model assumes that acoustic phonons are the main heat carriers and that three-phonon interactions dominate their scattering, its suitability for crystals with multiple atoms per unit cell is uncertain.[S47,S48]

The pressure dependence of thermal conductivity for an isotropic solid under isochoric conditions with an monoatomic basis is well-explained by theory of Liebfried and Schlömann (LS) and can be estimated as,[S46,S49]

$$\kappa = A\frac{V^{1/3}{\omega_D}^3}{\gamma^2 T} \tag{S2}$$

where V is the volume, ω_D is Debye frequency, γ is the Grüneisen parameter and T is the temperature and A is a parameter independent of presure. Assuming that the Poisson ratio and the elastic anisotropy parameter remain roughly constant under pressure, then Debye frequency $\omega_D \propto V^{1/6}\sqrt{K_T}$, where K_T is the isothermal bulk modulus at pressure P. Under isothermal conitions, the LS equation simply deduces to $\kappa \propto AK_T^{3/2}$. The isothermal bulk modulus given by K_T = -dP/d ln$V$ is calculated as a function of pressure using our MLP-MD bulk moduli simulations for our LiBr and LiI computational domains. The best fit to our thermal conductivity and bulk moduli data gives A_{LiBr} = 0.006375 W m^{-1} K^{-1} GPa$^{-1.5}$ and A_{LiI} = 0.011 W m^{-1} K^{-1} GPa$^{-1.5}$. We observe a good agreement between our MLP-based predictions and LS estimates within statistical uncertainties as shown in Fig. S34.

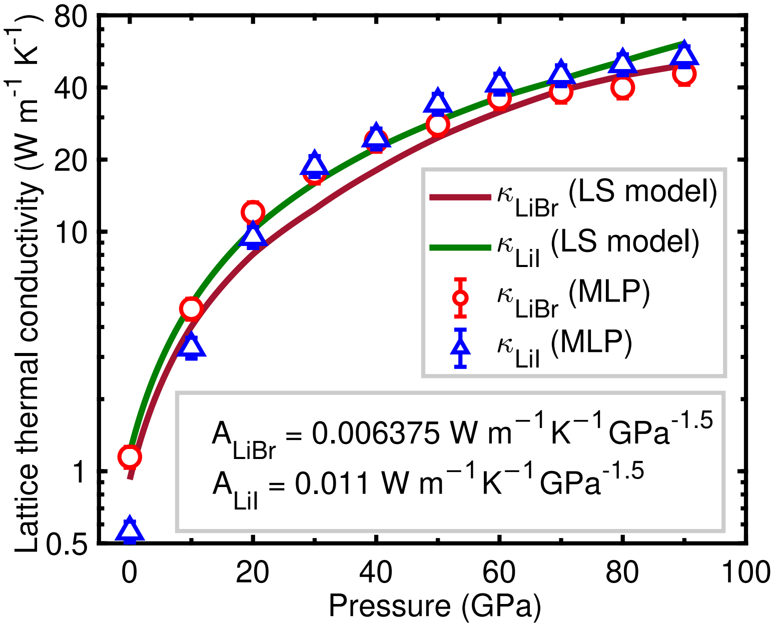

Figure S34: Comparison of our MLP-based pressure dependence of thermal conductivity for lithium halides (LiBr and LiI) with that estimated using Liebfried and Schlömann (LS) equation. There is a good agreement between our MLP-based predictions and LS estimates within statistical uncertainties.

References

(S1) Wang, H.; Zhang, L.; Han, J.; Weinan, E. DeePMD-kit: A deep learning package for many-body potential energy representation and molecular dynamics. *Comput. Phys. Commun.* **2018**, *228*, 178–184.

(S2) Marzari, N.; Mostofi, A. A.; Yates, J. R.; Souza, I.; Vanderbilt, D. Maximally localized Wannier functions: Theory and applications. *Rev. Mod. Phys.* **2012**, *84*, 1419–1475.

(S3) Zhang, L.; Chen, M.; Wu, X.; Wang, H.; E, W.; Car, R. Deep neural network for the dielectric response of insulators. *Phys. Rev. B* **2020**, *102*, 041121.

(S4) Mostofi, A. A.; Yates, J. R.; Lee, Y.-S.; Souza, I.; Vanderbilt, D.; Marzari, N. wannier90: A tool for obtaining maximally-localised Wannier functions. *Comput. Phys. Commun.* **2008**, *178*, 685–699.

(S5) Giannozzi, P.; Baroni, S.; Bonini, N.; Calandra, M.; Car, R.; Cavazzoni, C.; Ceresoli, D.;

Chiarotti, G. L.; Cococcioni, M.; Dabo, I.; others QUANTUM ESPRESSO: a modular and open-source software project for quantum simulations of materials. *J. Phys.: Condensed Matter.* **2009**, *21*, 395502.

(S6) Pettersson, S. Calculation of the thermal conductivity of alkali halide crystals. *J. Phys. C: Solid State Phys.* **1987**, *20*, 1047–1061.

(S7) Hakansson, B.; Ross, R. G. Thermal conductivity and heat capacity of solid LiBr and RbF under pressure. *J. Phys.: Condensed Matter* **1989**, *1*, 3977–3985.

(S8) Wang, J.; Deng, M.; Chen, Y.; Liu, X.; Ke, W.; Li, D.; Dai, W.; He, K. Structural, elastic, electronic and optical properties of lithium halides (LiF, LiCl, LiBr, and LiI): First-principle calculations. *Mater. Chem. Phys.* **2020**, *244*, 122733.

(S9) Hill, R. The elastic behaviour of a crystalline aggregate. *Proc. Phys. Soc. A.* **1952**, *65*, 349–354.

(S10) Marshall, B.; Cleavelin, C. Elastic constants of LiBr from 300° to 4.2° K. *J. Phys. Chem. Solids.* **1969**, *30*, 1905–1908.

(S11) Kittel, C.; McEuen, P. *Introduction to Solid State Physics*; John Wiley & Sons: New Jersey, **2010**.

(S12) Murnaghan, F. D. The compressibility of media under extreme pressures. *Proc. Natl. Acad. Sci.* **1944**, *30*, 244–247.

(S13) Birch, F. Finite elastic strain of cubic crystals. *Phys. Rev.* **1947**, *71*, 809–824.

(S14) Tyuterev, V.; Vast, N. Murnaghan's equation of state for the electronic ground state energy. *Comput. Mater. Sci.* **2006**, *38*, 350–353.

(S15) Hirosaki, N.; Ogata, S.; Kocer, C.; Kitagawa, H.; Nakamura, Y. Molecular dynamics calculation of the ideal thermal conductivity of single-crystal α- and β-Si_3N_4. *Phys. Rev. B* **2002**, *65*, 134110.

(S16) Volz, S. G.; Chen, G. Molecular-dynamics simulation of thermal conductivity of silicon crystals. *Phys. Rev. B* **2000**, *61*, 2651–2656.

(S17) Ohtori, N.; Ishii, Y.; Togawa, Y.; Oono, T.; Takase, K. Thermal conductivity of simple liquids: Temperature and packing-fraction dependence. *Phys. Rev. E* **2014**, *89*, 022129.

(S18) Dongre, B.; Wang, T.; Madsen, G. K. Comparison of the Green–Kubo and homogeneous non-equilibrium molecular dynamics methods for calculating thermal conductivity. *Model. Simul. Mater. Sci. Eng.* **2017**, *25*, 054001.

(S19) Deng, J.; Stixrude, L. Thermal conductivity of silicate liquid determined by machine learning potentials. *Geophys. Res. Lett.* **2021**, *48*, 1–10.

(S20) Korotaev, P.; Novoselov, I.; Yanilkin, A.; Shapeev, A. Accessing thermal conductivity of complex compounds by machine learning interatomic potentials. *Phys. Rev. B* **2019**, *100*, 144308.

(S21) Takeshita, Y.; Shimamura, K.; Fukushima, S.; Koura, A.; Shimojo, F. Thermal conductivity calculation based on Green–Kubo formula using ANN potential for β-Ag_2Se. *J. Phys. Chem. Solids.* **2022**, *163*, 110580.

(S22) Che, J.; Huang, W.; Ren, G.; Linghu, J.; Wang, X. Dual-channel phonon transport leads to low thermal conductivity in pyrochlore $La_2Hf_2O_7$. *Ceram. Int.* **2024**, *50*, 22865–22873.

(S23) Chen, R.; Tian, Y.; Cao, J.; Ren, W.; Hu, S.; Zeng, C. Unified deep learning network for enhanced accuracy in predicting thermal conductivity of bilayer graphene, hexagonal boron nitride, and their heterostructures. *J. Appl. Phys.* **2024**, *135*, 145106.

(S24) Bhatt, N.; Karna, P.; Thakur, S.; Giri, A. Transition from electron-dominated to phonon-driven thermal transport in tungsten under extreme pressures. *Phys. Rev. Mater.* **2023**, *7*, 115001.

(S25) Bhatt, N.; Karna, P.; Thakur, S.; Giri, A. Pressure-driven enhancement of phonon contribution to the thermal conductivity of Iridium. *Int. J. Heat Mass Transf.* **2024**, *229*, 125673.

(S26) Li, R.; Lee, E.; Luo, T. A unified deep neural network potential capable of predicting thermal conductivity of silicon in different phases. *Mater. Today Phys.* **2020**, *12*, 100181.

(S27) Odegard, G. M.; Patil, S. U.; Deshpande, P. P.; Kanhaiya, K.; Winetrout, J. J.; Heinz, H.; Shah, S. P.; Maiaru, M. Molecular dynamics modeling of epoxy resins using the reactive interface force field. *Macromolecules* **2021**, *54*, 9815–9824.

(S28) Kashmari, K.; Patil, S. U.; Kemppainen, J.; Shankara, G.; Odegard, G. M. Optimal molecular dynamics system size for increased precision and efficiency for epoxy materials. *J. Phys. Chem. B.* **2024**, *128*, 4255–4265.

(S29) Plimpton, S. Fast parallel algorithms for short-range molecular dynamics. *J. Comput. Phys.* **1995**, *117*, 1–19.

(S30) Hager, W. W.; Zhang, H. A survey of nonlinear conjugate gradient methods. *Pac. J. Optim.* **2006**, *2*, 35–58.

(S31) Guénolé, J.; Nöhring, W. G.; Vaid, A.; Houllé, F.; Xie, Z.; Prakash, A.; Bitzek, E. Assessment and optimization of the fast inertial relaxation engine (fire) for energy minimization in atomistic simulations and its implementation in lammps. *Comput. Mater. Sci.* **2020**, *175*, 109584.

(S32) Jacobson, P.; Stoupin, S. Thermal expansion coefficient of diamond in a wide temperature range. *Diam. Relat. Mater.* **2019**, *97*, 107469.

(S33) Madan, M. Temperature dependence of the bulk modulus of alkali halides. *J. Appl. Phys.* **1971**, *42*, 3888–3893.

(S34) Tadano, T.; Gohda, Y.; Tsuneyuki, S. Anharmonic force constants extracted from first-principles molecular dynamics: applications to heat transfer simulations. *J. Phys.: Condens. Matter* **2014**, *26*, 225402.

(S35) Perdew, J. P.; Burke, K.; Ernzerhof, M. Generalized gradient approximation made simple. *Phys. Rev. Lett.* **1996**, *77*, 3865–3868.

(S36) Yuan, K.; Zhang, X.; Tang, D.; Hu, M. Anomalous pressure effect on the thermal conductivity of ZnO, GaN, and AlN from first-principles calculations. *Phys. Rev. B* **2018**, *98*, 144303.

(S37) Leibfried, G.; Schlömann, E. *Wärmeleitung in elektrisch isolierenden Kristallen, von Günther Leibfried und Ernst Schlömann*; Vandenhoeck und Ruprecht: Göttingen, **1954**.

(S38) Adachi, S. *Properties of semiconductor alloys: group-IV, III-V and II-VI semiconductors*; John Wiley & Sons: New Jersey, **2009**.

(S39) Daoud, S.; Bouarissa, N.; Rekab-Djabri, H.; Saini, P. K. Structural and thermo-physical properties of 3C-SiC: high-temperature and high-pressure effects. *Silicon* **2022**, *14*, 6299–6309.

(S40) Ravichandran, N. K.; Broido, D. Non-monotonic pressure dependence of the thermal conductivity of boron arsenide. *Nat. Commun.* **2019**, *10*, 827–835.

(S41) Xia, H.; Xia, Q.; Ruoff, A. L. High-pressure structure of gallium nitride: Wurtzite-to-rocksalt phase transition. *Phys. Rev. B* **1993**, *47*, 12926–12928.

(S42) Kim, D.; Smith, R. F.; Ocampo, I. K.; Coppari, F.; Marshall, M. C.; Ginnane, M.; Wicks, J. K.; Tracy, S. J.; Millot, M.; Lazicki, A.; others. Structure and density of silicon carbide to 1.5 TPa and implications for extrasolar planets. *Nat. Commun.* **2022**, *13*, 2260–2269.

(S43) Xiao, J.; Du, J.; Wen, B.; Melnik, R.; Kawazoe, Y.; Zhang, X. Phase stability limit of c-BN under hydrostatic and non-hydrostatic pressure conditions. *J. Chem. Phys.* **2014**, *140*, 164704.

(S44) Deringer, V. L.; Tchougréeff, A. L.; Dronskowski, R. Crystal orbital Hamilton population (COHP) analysis as projected from plane-wave basis sets. *J. Phys. Chem. A.* **2011**, *115*, 5461–5466.

(S45) Dronskowski, R.; Bloechl, P. E. Crystal orbital hamilton populations (COHP): energy-resolved visualization of chemical bonding in solids based on density-functional calculations. *J. Phys. Chem. A* **1993**, *97*, 8617–8624.

(S46) Roufosse, M.; Klemens, P. Thermal conductivity of complex dielectric crystals. *Phys. Rev. B* **1973**, *7*, 5379–5386.

(S47) Steigmeier, E.; Kudman, I. Acoustical-optical phonon scattering in Ge, Si, and III-V compounds. *Phys. Rev.* **1966**, *141*, 767–774.

(S48) Ward, A.; Broido, D.; Stewart, D. A.; Deinzer, G. Ab initio theory of the lattice thermal conductivity in diamond. *Phys. Rev. B* **2009**, *80*, 125203.

(S49) Ross, R. G.; Andersson, P.; Sundqvist, B.; Backstrom, G. Thermal conductivity of solids and liquids under pressure. *Rep. Prog. Phys.* **1984**, *47*, 1347–1402.
