# Supplementary material for: Achieving Exceptionally Enhanced Thermal Conductivity and Bulk Modulus in Polar Insulators Via Modification of Chemical Bonding
Source: J Phys Chem Lett. 2025 Aug 21;16(34):8850–60. doi: 10.1021/acs.jpclett.5c01476 (PMC12422541; doi:10.1021/acs.jpclett.5c01476)
Supplement: Supplementary file 2 [file jz5c01476_si_002.pdf]

jz-2025-01476q.R1

Name: Peer Review Information for "Achieving exceptionally enhanced thermal conductivity and bulk modulus in polar insulators via modification of chemical bonding"

First Round of Reviewer Comments

Reviewer: 1

Comments to the Author

Bhatt et al. computationally investigate thermal transport in LiBr and LiL under varying hydrostatic pressure and observe large pressure-induced variations in thermal conductivity. However, there are several major issues that significantly undermine their conclusions.

1. Long-range Coulomb potentials. The long-range Coulomb field is missing in the MD simulations. Although the MLP is derived from ab initio MD, the finite size of the supercell in DFT calculations means that the MLP is essentially only capturing the short-range force constants. In polar materials, the harmonic phonon frequency requires the nonanalytic long-range force constants to properly describe the LO-TO splitting. As a result, all analysis based on MD, merely represents a neutral covalent material without any charges or dipoles, and exclude physics related to charges, dipoles, and LO-TO splitting. While the authors specifically computed the phonon dispersion including the LO-TO splitting, all other analysis is done based on simulations that does not include this effect. This inconsistency prevents accurate modeling of thermal conductivity.

2. Scattering rates calculations. To properly address this issue, the authors need to conduct the scattering rate calculation from third-order and fourth-order phonon scattering processes. The SED analysis from MD without long-range force constants cannot describe the phonon energy and phonon scattering properly. In fact, the ALAMODE package that the author used is already integrated with those functions (at least the third-order), and I am suppressed that the authors still prefer MD simulations. These formal scattering rates calculations allows for a proper analysis of the effect of phonon bunching and acoustic-optical gap on phonon scattering rates.

3. Electron Localization Function. The electron localization function calculation, in my opinion, does not give any insights on the bonding nature (e.g. ionic bonding, bonding or anti-bonding character). Instead, the authors can compute the charge density, a rather well-defined quantity, under the excitation of certain phonon modes and assess the pressure dependence of the long-range dipole field, or conduct the orbital overlap analysis.

4. Overlap Argument in Figure 2. The energy overlap argument in fig. 2 is very weak as I cannot understand why this energy change has to lead a very large modulus change.

5. DeepMD Thermal Conductivity Benchmarking. For the DeepMD thermal conductivity workflow, can the authors provide some solid benchmark calculations. For example, is thermal conductivity of GaAs or GaN, which are very accurately computed using Boltzmann Transport Equation formalism, reproducible?

6. Applying pressures in the GPa range requires bulky mechanical components, which introduce significant dead thermal mass and compromise the efficacy of thermal switching. If the computational support were stronger, I do not think the authors would need to include this discussion to motivate their writing (this is a minor issue).

7. Typo: BaAs should be BAs.

Overall, the authors' methodology is questionable for properly studying a polar material, which leads to potentially inaccurate conclusions. As such, the conclusions are unconvincing.

Reviewer: 2

Comments to the Author

This manuscript presents a theoretical study on the impacts of pressure on the thermal conductivity of lithium halides. A two orders of magnitude change was reported, and the underlying mechanism was discussed. Overall, this paper is well-structured, innovative, and aligns with the basic interests of the research community. However, there are some aspects that need clarification, some of which may affect the overall quality. Therefore, I recommend publishing this paper after major revisions. The following are some issues that need attention:

1.The change in thermal conductivity is attributed to bonding characteristic. In addition to this factor, geometry also affects the lifetime of phonon, then changes thermal conductivity, refer to National Science Review, 8(9), nwaa220, (https://doi.org/10.1093/nsr/nwaa220). The authors should check this point.

2. Although the results are promising, a discussion on the challenges in experimental observation can also be included.

3. Figure 4g presents the impact of pressure on temperature dependence. Detailed physical analysis should be provided, for example, the temperature dependent SED.

4. Active control of thermal conductivity is a hot topic in recent years, I noticed that there is room for further citation of articles closely related to the research topic, for example, Nanoscale, 13, 1425 (2021); Physics Reports 1058, 1–32 (2024). This will help enrich the content of the paper, enhance its academic value, and make it easier for readers to understand the perspective.

Reviewer: 3

Comments to the Author

In the manuscript, the authors have investigated the thermal conductivity and bulk modulus of LiI and LiBr by using molecular dynamics (MD) simulations together with machine learning potentials (MLPs). This paper is well written, but there are some issues needed to be addressed.

1. The accuracy of the present MLPs needs some validation. The authors can present a comparison of the thermal conductivity and bulk modulus extracted from the present MLPs and density functional theory calculations or experiments.

2. The method employed here to calculate the bulk modulus was based on the slope of two points in the P-V curve. This method is too rough. I recommend the authors to try some other methods such as the strain-fluctuation methods.

3. The thermal conductivity and bulk modulus similarly increase with growing pressure. Do there exist some relevance between these two parameters at different pressures?

4. How about the bulk modulus at different temperature?

5. It is surprising that the bulk moduli of LiI and LiBr significantly increase with growing pressure. The authors should give some explanation to this phenomenon.

Author's Response to Peer Review Comments:

The authors greatly appreciate the comments and thorough reviews of the manuscript from the reviewers. The added insights from the reviewers have provided invaluable feedback that have ultimately bolstered the discussion in the manuscript and increased its' scope. In accordance with the comments from the reviewers, we have made revisions and performed additional calculations as detailed below.

The reviewer's comments are listed verbatim, and our responses immediately follow in **bold.**

Reviewer: 1

Comments to the Authors

Bhatt et al. computationally investigate thermal transport in LiBr and LiL under varying hydrostatic pressure and observe large pressure-induced variations in thermal conductivity. However, there are several major issues that significantly undermine their conclusions.

The authors are grateful to the reviewer for a thorough and detailed review of the manuscript and providing us an opportunity to refine our manuscript.

1. Long-range Coulomb potentials. The long-range Coulomb field is missing in the MD simulations. Although the MLP is derived from ab initio MD, the finite size of the supercell in DFT calculations means that the MLP is essentially only capturing the short-range force constants. In polar materials, the harmonic phonon frequency requires the nonanalytic long-range force constants to properly describe the LO-TO splitting. As a result, all analysis based on MD, merely represents a neutral covalent material without any charges or dipoles, and exclude physics related to charges, dipoles, and LO-TO splitting. While the authors specifically computed the phonon dispersion including the LO-TO splitting, all other analysis is done based on simulations that does not include this effect. This inconsistency prevents accurate modeling of thermal conductivity.

We appreciate the reviewer's thoughtful feedback on our work. We fully acknowledge the importance of long-range interactions for accurately capturing LO-TO splitting in polar materials such as lithium halides, which we have accounted for in our phonon dispersion calculations, as the reviewer correctly noted. As the reviewer also pointed out, AIMD is inherently limited to short-range interactions, and thus long-range Coulomb interactions are typically treated by using the K-space command in LAMMPS to implement a long-range solver. While this remains an approximation, in response to the reviewer's suggestion, we have developed a machine learning potential that explicitly includes long-range interactions using Wannier centers, following the approach by Zhang et al. (*J. Chem. Phys.* 156, 124107, 2022) in their Deep Potential model incorporating long-range electrostatics ("A deep potential model with long-range electrostatic interactions").

As demonstrated by our results (shown below for the reviewer's consideration), explicitly incorporating these long-range interactions through the Deep Potential Long Range (DPLR) model does not alter our findings or conclusions at low pressures. Due to time constraints, the DPLR model was applied only to the low-pressure regime. However, at higher pressures, the material transitions from a polar ionic solid to one with more covalent bonding. Given that explicit inclusion of long-range effects did not impact thermal conductivity or bulk modulus outcomes at low pressures, we do not anticipate significant changes in our results at higher pressures either.

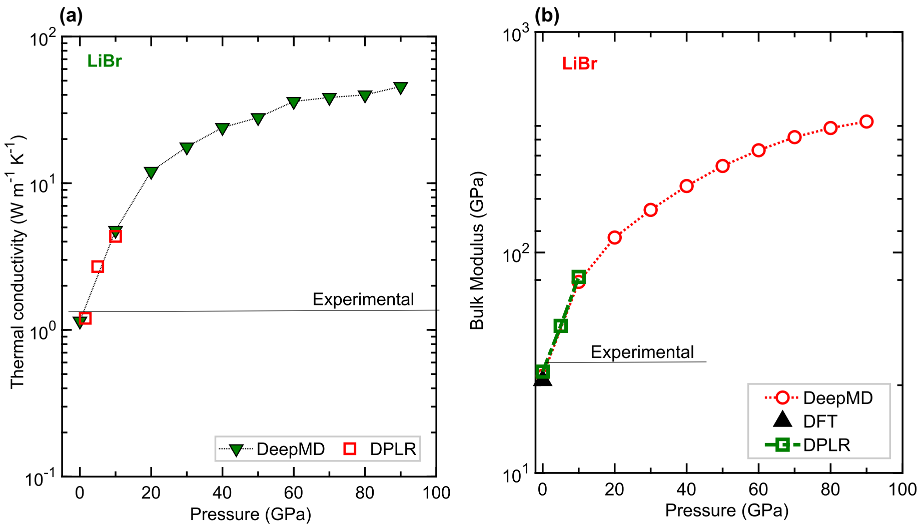

Figure S11: We developed a new potential using the Deep Potential Long-Range (DPLR) framework, which explicitly includes long-range electrostatic interactions for the low-pressure regime. At low pressures, where the system exhibits polar and ionic characteristics, we observe strong consistency in the predictions of (a) thermal conductivity and (b) bulk modulus between the DPLR and DeepMD models. Additionally, our computational results are supported by experimental data obtained under ambient conditions. [S7-S10]

In page S7 of the revised Supporting Information, we add,

"We developed a new machine learning potential (MLP) for LiBr that incorporates long-range electrostatic interactions using the Deep Potential Long-Range (DPLR) model. This model combines the short-range accuracy of DeepMD with explicit long-range interactions calculated via a Gaussian charge model known as the Deep Wannier (DW) framework. The system was constructed using a 2×2×2 supercell of LiBr (64 atoms) at 300 K and 0 GPa. The DPLR framework utilizes two neural networks: the DeepMD network for short-range forces and a pretrained DW network to predict long-range electrostatics using Ewald summation. Wannier centers, representing charge centroids, are predicted from atomic environments using the DW model, trained to reproduce centers derived from DFT-calculated maximally localized Wannier functions (MLWFs). The DPLR model was then jointly optimized to fit DFT energies, forces, and stresses. The total potential energy surface (PES) thus captures both short- and long-range interactions.[S1] The long-range energy is modeled as the interaction of spherical Gaussian charges, where the charges are split between fixed ion cores and the dynamically computed valence electron centers (i.e., Wannier centroids). [S2-S3] **The Wannier centers were calculated using *wannier90*[S4] interfaced with Quantum ESPRESSO,[S5] employing SCF and NSCF DFT calculations with PBE functionals. These MLWFs transform delocalized Kohn-Sham orbitals into localized charge densities. A total of 192 Wannier centers were obtained for LiBr, forming the dataset for training. A final DPLR model was trained over 5 million steps with an exponentially decaying learning rate (10^{-3} to 10^{-8}), and a dynamic loss function initializing the pre-factors of energies and forces as $P_e^{start} = 0.01$, $P_f^{start} = 1000$, $P_e^{limit} = 1$, and $P_f^{limit} = 1$, respectively Ewald summation (β=0.1) was used to**

compute electrostatic contributions, and automatic batching handled the training dataset efficiently. The resulting model achieves high fidelity in reproducing both short- and long-range interactions critical for accurately describing ionic dynamics in LiBr. Training and validation results confirm excellent agreement between predicted and reference DFT forces and energies as evidenced from Fig. S8."

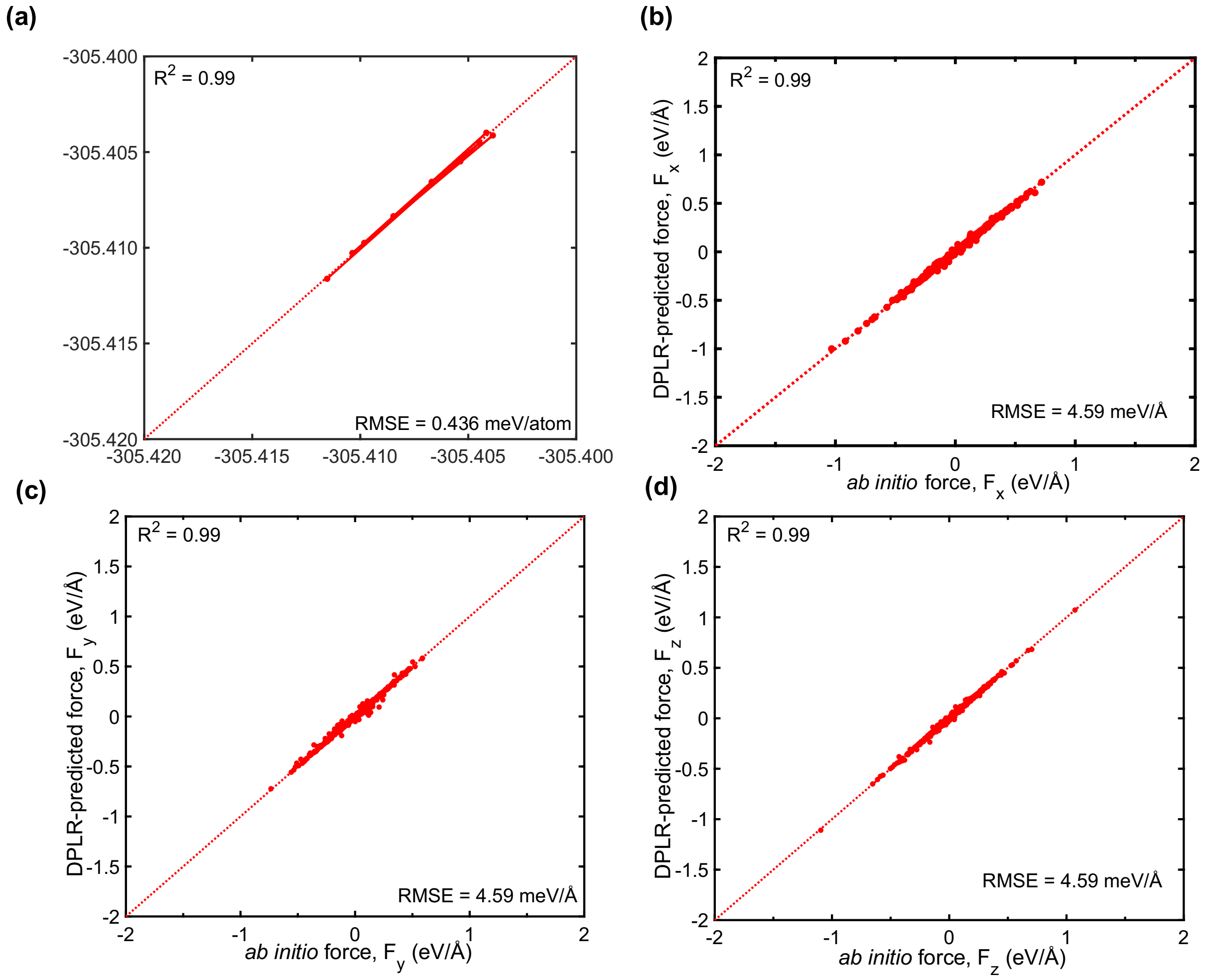

Figure S8: Validation plots comparing (a) energies and (b-d) forces given by our DPLR model with ab initio MD counterparts for LiBr at 0 Gpa. The perfect *x=y* agreement of DPLR-predicted vs. ab initio energies and forces reflected in R^2 value close to unity and low RMSE ascertains the accuracy of our DPLR model at ambient conditions.

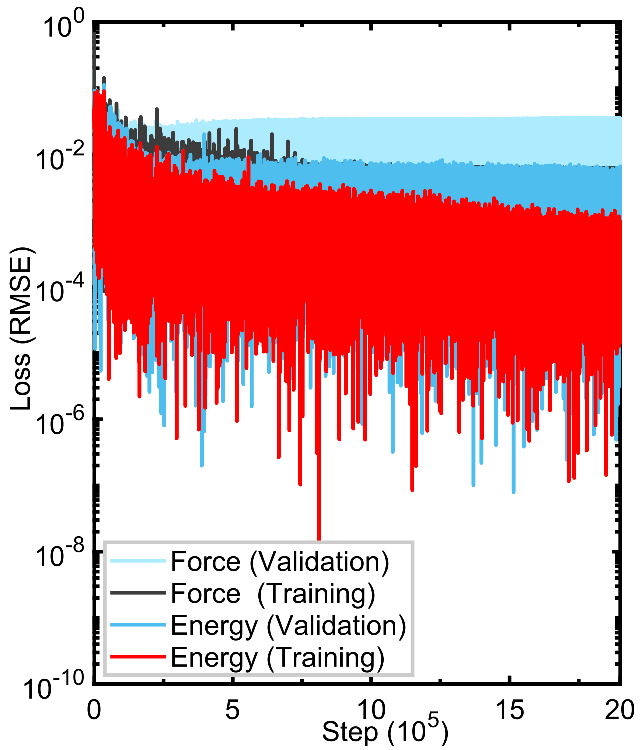

Figure S9: Evolution of RMSE scores during training for the Deep Potential Long-Range (DPLR) model, demonstrating high accuracy as demonstrated by consistent convergence of learning curves for energy and force components. Errors diminish to very low magnitudes, confirming the accuracy of our trained DPLR model.

In the main manuscript page 20, we add,

"In MD simulations, long-range interactions are typically handled using the K-space command in LAMMPS, which invokes a long-range electrostatics solver. In addition to this, we have developed a machine learning potential based on the Deep Potential Long-Range (DPLR) model[56] for the low-pressure regime where the materials are predominantly polar. As shown in Figure S11, the strong agreement between the results from the DPLR and DeePMD potentials supports the validity of our approach using the DeePMD framework."

Supporting Information references:

[S7] Hakansson, B.; Ross, R. G. Thermal conductivity and heat capacity of solid LiBr and RbF under pressure. *Journal of Physics: Condensed Matter* 1989, *1*, 3977.

[S8] Wang, J.; Deng, M.; Chen, Y.; Liu, X.; Ke, W.; Li, D.; Dai, W.; He, K. Structural, elastic, electronic and optical properties of lithium halides (LiF, LiCl, LiBr, and LiI): First-principle calculations. *Materials Chemistry and Physics* 2020, 244, 122733.

[S9] Hill, R. The elastic behaviour of a crystalline aggregate. *Proceedings of the Physical Society*. Section A 1952, 65.

[S10] Marshall, B.; Cleavelin, C. Elastic constants of LiBr from 300° to 4.2° K. *Journal of Physics and Chemistry of Solids* 1969, 30, 1905–1908.

[S11] Kittel, C.; McEuen, P. *Introduction to solid state physics*; John Wiley & Sons, 2018.

[S6] Pettersson, S. Calculation of the thermal conductivity of alkali halide crystals. *Journal of Physics C: Solid State Physics* 1987, 20, 1047.

[S7] Hakansson, B.; Ross, R. G. Thermal conductivity and heat capacity of solid LiBr and RbF under pressure. *Journal of Physics: Condensed Matter* 1989, 1, 3977.

[S15] Hirosaki, N.; Ogata, S.; Kocer, C.; Kitagawa, H.; Nakamura, Y. Molecular dynamics calculation of the ideal thermal conductivity of single-crystal α-and β-Si_3N^4. *Physical Review B* 2002, 65, 134110.

[S16] Volz, S. G.; Chen, G. Molecular-dynamics simulation of thermal conductivity of silicon crystals. *Physical Review B* 2000, 61, 2651.

[S17] Ohtori, N.; Ishii, Y.; Togawa, Y.; Oono, T.; Takase, K. Thermal conductivity of simple liquids: Temperature and packing-fraction dependence. *Physical Review E* 2014, 89, 022129.292 S35

[S18] Dongre, B.; Wang, T.; Madsen, G. K. Comparison of the Green–Kubo and homogeneous293 non-equilibrium molecular dynamics methods for calculating thermal conductivity. *Modelling and Simulation in Materials Science and Engineering* 2017, 25, 054001.

[S19] Deng, J.; Stixrude, L. Thermal conductivity of silicate liquid determined by machine learning potentials. *Geophysical Research Letters* 2021, 48, e2021GL093806.

[S20] Korotaev, P.; Novoselov, I.; Yanilkin, A.; Shapeev, A. Accessing thermal conductivity of complex compounds by machine learning interatomic potentials. *Physical Review B* 2019,299 100, 144308.

[S21] Takeshita, Y.; Shimamura, K.; Fukushima, S.; Koura, A.; Shimojo, F. Thermal conductivity calculation based on Green–Kubo formula using ANN potential for β-Ag2Se. *Journal of Physics and Chemistry of Solids* 2022, 163, 110580.

[S22] Che, J.; Huang, W.; Ren, G.; Linghu, J.; Wang, X. Dual-channel phonon transport leadsto low thermal conductivity in pyrochlore $La_2Hf_2O_7$. *Ceramics International* 2024, 50, 22865–22873.

[S23] Chen, R.; Tian, Y.; Cao, J.; Ren, W.; Hu, S.; Zeng, C. Unified deep learning network for enhanced accuracy in predicting thermal conductivity of bilayer graphene, hexagonal boron nitride, and their heterostructures. *Journal of Applied Physics* 2024, 135.

[S24] Bhatt, N.; Karna, P.; Thakur, S.; Giri, A. Transition from electron-dominated to phonon-driven thermal transport in tungsten under extreme pressures. *Physical Review Materials* 2023, 7, 115001.

[S25] Bhatt, N.; Karna, P.; Thakur, S.; Giri, A. Pressure-driven enhancement of phonon contribution to the thermal conductivity of Iridium. *International Journal of Heat and Mass Transfer* 2024, 229, 125673.

[S26] Li, R.; Lee, E.; Luo, T. A unified deep neural network potential capable of predicting thermal conductivity of silicon in different phases. *Materials Today Physics* 2020, 12, 100181.

Furthermore, we have performed additional spectral energy density (SED) calculations using the DPLR model and compared them with the SEDs from the DeepMD model, as shown in Fig. S12 of the Supporting Information (and shown below for the reviewer's consideration). We see negligible differences in the SEDs with the inclusion of long-range electrostatic interactions further reinforcing the reliability of our MLPs.

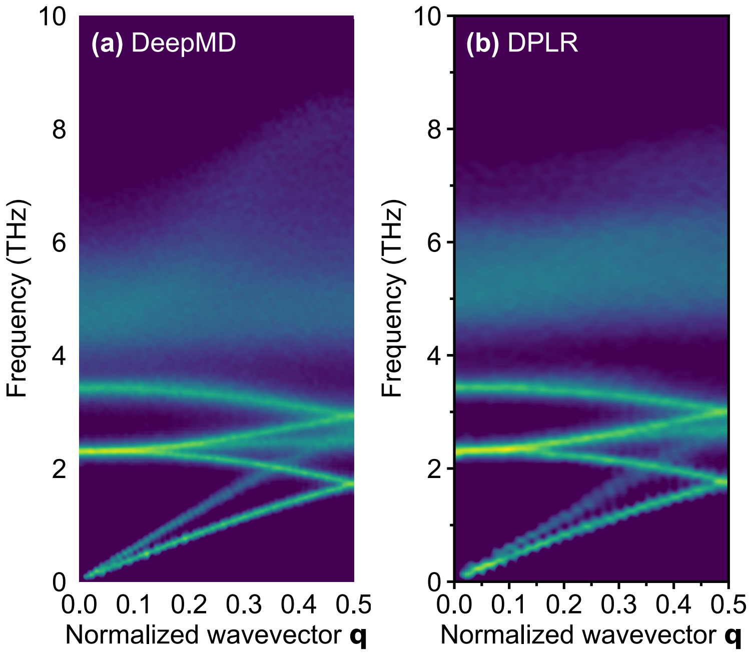

Figure S12: Calculated phonon spectral energy densities (SEDs) for LiBr at 300 K using DeepMD and DPLR models. We do not observe any significant differences in the SEDs with the inclusion of long-range electrostatic interactions.

2. Scattering rates calculations. To properly address this issue, the authors need to conduct the scattering rate calculation from third-order and fourth-order phonon scattering processes. The SED analysis from MD without long-range force constants cannot describe the phonon energy and phonon scattering properly. In fact, the ALAMODE package that the author used is already integrated with those functions (at least the third-order), and I am suppressed that the authors still prefer MD simulations. These formal scattering rates calculations allows for a proper analysis of the effect of phonon bunching and acoustic-optical gap on phonon scattering rates.

We concur with the reviewer that calculating third- and fourth-order phonon scattering rates could offer additional insight into the phonon scattering mechanisms in these materials. However, due to the time constraints involved in training students in these advanced

methodologies, particularly given the added complexity of incorporating long-range interactions, such calculations fall beyond the current scope of our work. Nonetheless, we hope that our study will stimulate future investigations that leverage first-principles-based third- and fourth-order scattering analyses to further understand the roles of phonon bunching and the acoustic-optical gap in determining phonon lifetimes.

It is important to note that our spectral energy density (SED) approach inherently captures the full anharmonic behavior of the system and is not restricted to specific scattering orders. Furthermore, we have extended our SED calculations using the newly developed DPLR model with explicit long-range interactions, and we observe that the scattering rates remain consistent with our earlier findings. Lastly, we emphasize that the primary objective of this study is to reveal the significant pressure-induced changes in thermal and mechanical properties. We believe that the overall conclusions of our work remain robust irrespective of the specific method used to evaluate scattering rates.

In pages 13-14 of the revised manuscript, we add,

"While the scattering rates from our SEDs provide some insights into the lifetimes of the different phonon modes, a detailed calculations of first-principles-based three- and four-phonon scattering may help shed more light on the effects of acoustic phonon bunching and the changes in the acoustic-optic gap on the phonon scattering rates, which is beyond the scope of the current work but deserves further consideration."

3. Electron Localization Function. The electron localization function calculation, in my opinion, does not give any insights on the bonding nature (e.g. ionic bonding, bonding or anti-bonding character). Instead, the authors can compute the charge density, a rather well-defined quantity, under the excitation of certain phonon modes and assess the pressure dependence of the long-range dipole field, or conduct the orbital overlap analysis.

We appreciate the reviewer's observation and acknowledge the need for greater clarity in our description of the charge density analysis. In addition to the electron localization function (ELF), we have performed charge density difference calculations for LiBr and LiI at 0 and 90 GPa, as presented in Fig. 2g–j. Such charge density difference methods have been employed in prior studies to explore bonding characteristics [1–3]. These calculations illustrate how electron density redistributes as atoms form a crystal lattice. At ambient pressure, the charge density is largely concentrated around the halogen atoms, indicating strong ionic character. With increasing pressure, the electron density becomes more delocalized and shared between atoms, suggesting a transition toward more covalent-like bonding. This pressure-induced redistribution is not readily apparent from ELF plots alone, as the reviewer noted. Therefore, we believe these visualizations are essential for capturing the evolving bonding nature under pressure. We have also clarified in the revised manuscript that this approach has been widely used to differentiate between covalent and ionic bonding.

As suggested by the reviewer, we also carry out the orbital overlap analysis to support these results. As shown by the results of the Crystal orbital Hamiltonian Population (COHP) calculations, the bonding nature clearly increases for the pressurized cases with covalent

bonds. These calculations provide further support for the results shown in our manuscript, therefore we thank the reviewer for the suggestion.

In page S24 of the Supporting Information, we state,

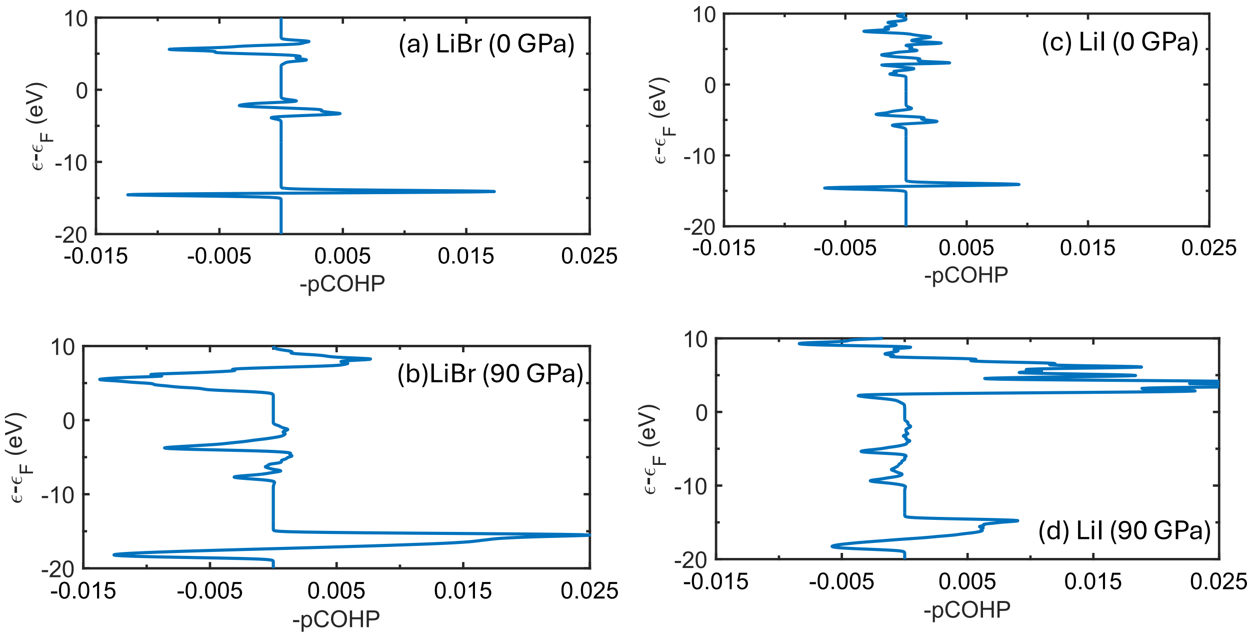

Figure S24: Crystal orbital Hamiltonian Population (COHP) for LiBr and LiI at 0 and 90 GPa. The COHP calculation shows that the bonding characteristic increases with increase in pressure.

"We investigate the chemical bonding in LiBr and LiI using the Crystal Orbital Hamilton Population (COHP) method implemented in the LOBSTER code.[S44,S45] COHP allows the decomposition of the electronic band energy into individual atomic or orbital interactions, classifying them as bonding (positive), anti-bonding (negative), or non-bonding (zero).[S44,S45] Our calculations reveal that as pressure increases, the bonding interactions become stronger, indicating enhanced covalent character. This quantitatively supports our observations of electron density localization between the lithium atoms and the halide anions, leading a greater covalent bonding character at higher pressures."

Supporting Information References:

[S44] Deringer, V. L., Tchougréeff, A. L. & Dronskowski, R. Crystal Orbital Hamilton Population (COHP) Analysis As Projected from Plane-Wave Basis Sets. J. Phys. Chem. A 115, 5461-5466 (2011).

[S45] Dronskowski, R. & Bloechl, P. E. Crystal orbital Hamilton populations (COHP): energy-resolved visualization of chemical bonding in solids based on density-functional calculations. J. Phys. Chem. 97, 8617-8624 (1993).

Review References:

[1] Sahara. R, Shishido. T, Nomura A, Kundou. K, Okada. S, Kumar. V, Nakajima. K, Kawazoe. Y, Physical Review B 73, 184102 (2006).

[2] L. Su, L. Wan, T. Gao, B. Ao, 10(7), 2020.

[3] A. Ranganathan, G. U. Kulkarni, C. N. R. Rao, Journal of Molecular Structure, 656 (1-3), 2003.

4. Overlap Argument in Figure 2. The energy overlap argument in fig. 2 is very weak as I cannot understand why this energy change has to lead a very large modulus change

Our charge density and orbital overlap calculations indicate that the covalent character of the bonding increases with pressure in both LiI and LiBr, driven by enhanced orbital overlap, which is now supported by the additional COHP calculations that the reviewer recommended.

Covalent character can be enhanced either through greater spatial overlap or by a reduction in the energy difference between the parent atomic orbitals [4–6]. As such, a positive correlation exists between increased orbital overlap and higher bulk modulus. Similar arguments have been made in prior studies [7–10]. For example, Sahara *et al.* (*Physical Review B* 73, 184102, 2006) showed that doping $ScRh_3B_x$ with boron enhances orbital overlap and therefore the covalent bonding nature around the Rh atom, which in turn leads to an increase in the bulk modulus of the material. Similarly, in our case, the increased energy overlap of atomic orbitals under pressure strengthens covalent bonding, playing a key role in the enhancement of the bulk modulus in these halides. We add the following discussion in the revised manuscript in page 9 as follows:

"Covalent bonding strength can be increased either by enhancing spatial overlap or by narrowing the energy difference between the parent atomic orbitals.[35,36] Consequently, greater orbital overlap is positively linked to a higher bulk modulus. Similar increase in bulk modulus due to increase in covalent bonding characteristic has been previously shown in various other studies.[37–39]"

Review References:

[4] E. Lu, S. Sajjad, V.E>J. Berryman, A. J. Wooles, N. Kaltsoyannis and S. T. Liddle, *Nature Communications* 10, 634 (2019).

[5] M. L. Neidig, D. L. Clark, R. L. Martin, *Coordination Chemistry Reviews* 257 (2), 2013.

[6] Nikolas Kaltsoyannis, Inorganic Chemistry, 52(7), 2012.

[7] Sahara. R, Shishido. T, Nomura A, Kundou. K, Okada. S, Kumar. V, Nakajima. K, Kawazoe. Y, *Physical Review B* 73, 184102 (2006).

[8] Yang. Z, Dai. C, Sun. J, Lu. S, Li. W, Li. X, Li. X, Vitos. L, *Computaional Materials Science* 194 (2021) 110469.

[9] Y. Pan, Y. H. Lin, M. Wen, Q.N. Meng, Correlation between hardness and pressure of CrB_4, *RSC Advances*.

[10] A M Ito, A Takayama, Y Oda and H Nakamura, *Journal of Physics: Conference Series* 518 (2014) 012011

Manuscript References:

[35] Lu, E.; Sajjad, S.; Berryman, V. E. J.; Wooles, A. J.; Kaltsoyannis, N.; Liddle, S. T. Emergence of the structure-directing role of f-orbital overlap-driven covalency. *Nature Communications* 2019, 10, 634.

[36] Kaltsoyannis, N. Does Covalency Increase or Decrease across the Actinide Series? Implications for Minor Actinide Partitioning. *Inorganic Chemistry* 2013, 52, 3407–3413.

[37] Sahara, R.; Shishido, T.; Nomura, A.; Kudou, K.; Okada, S.; Kumar, V.; Nakajima, K.; Kawazoe, Y. Mechanism of the increase in bulk modulus of perovskite ScRh3Bx by vacancies. *Phys. Rev. B* 2006, 73, 184102.

[38] Yang, Z.; Dai, C.; Sun, J.; Lu, S.; Li, W.; Li, X.; Li, X.; Vitos, L. Theoretically exploring covalent bonding effect on deformability of B2/beta Ti(AlxNb1-x) phase. *Computational Materials Science* 2021, 194, 110469.

[39] Pan, Y.; Lin, Y. H.; Wen, M.; Meng, Q. N. Correlation between hardness and pressure of CrB4. RSC Adv. 2014, 4, 63891–63896.

5. DeepMD Thermal Conductivity Benchmarking. For the DeepMD thermal conductivity workflow, can the authors provide some solid benchmark calculations. For example, is thermal conductivity of GaAs or GaN, which are very accurately computed using Boltzmann Transport Equation formalism, reproducible?

Developing a machine-learned potential for GaAs or GaN, as suggested by the reviewer, is well beyond the scope of the current study. However, the DeePMD approach has been extensively validated through numerous benchmark studies, demonstrating reliable thermal conductivity predictions in line with both DFT–BTE calculations and experimental data for different material systems [11–12]. Of particular note are accurate predictions for various silicon phases, which closely align with experimental and ab initio results using the DeePMD framework [13]. Furthermore, as shown in the accompanying figure, our DeePMD model reproduces both the experimentally measured thermal conductivity and bulk modulus for LiBr, further reinforcing the reliability of our approach.

In page 20 of the revised manuscript, we add,

"As shown in Figure S11, the strong agreement between the results from DPLR and DeePMD potentials (along with the agreement with experimentally measured bulk modulus and

thermal conductivity at ambient for LiBr) supports the validity of our approach using the DeePMD framework."

In our revised supplemental material in page S16, we state:

"Our machine learning potential (MLP) demonstrates strong predictive capability for both the mechanical and thermal properties of lithium halides, yielding values in close quantitative agreement with prior experimental studies, as shown in Fig. S11. For LiBr, the MLP predicts a bulk modulus of 28.02 GPa, which is remarkably consistent with DFT (29.94 GPa), experimental results (28.5 GPa), and earlier theoretical reports (26.3 GPa) using Voigt-Reuss-Hill method. Likewise, for LiI, the predicted bulk modulus of 22.73 GPa aligns well with DFT (22.67 GPa) and theoretical estimates (22.0 GPa).[S8–S10]

Beyond elasticity, the MLP captures thermal transport trends with similar accuracy, predicting a thermal conductivity of 1.14 W m^{-1} K^{-1} for LiBr. This value is in reasonable agreement with the experimental measurement of 1.8 W m^{-1} K^{-1} and matches the theoretical value of 1.31 W m^{-1} K^{-1} within the reported 15–20% statistical uncertainties.[S6,S7] The close agreement across DFT, experimental, and theoretical benchmarks confirms the robustness of our MLP and supports its application for extended simulations and predictive studies of lithium halides."

Review References:

[11] Junwei Che, Wenjie Huang, Guoliang Ren, Jiajun Linghu, and Xuezhi Wang. 2024. Dual-channel phonon transport leads to low thermal conductivity in pyrochlore La2Hf2O7. *Ceramics International* 50, 13 (2024), 22865–22873.

[12] Rongkun Chen, Yu Tian, Jiayi Cao, Weina Ren, Shiqian Hu, and Chunhua Zeng. 2024. Unified deep learning network for enhanced accuracy in predicting thermal conductivity of bilayer graphene, hexagonal boron nitride, and their heterostructures. *Journal of Applied Physics* 135, 14 (2024).

[13] Ruiyang Li, Eungkyu Lee, and Tengfei Luo. 2020. A unified deep neural network potential capable of predicting thermal conductivity of silicon in different phases. *Materials Today Physics* 12 (2020), 100181.

Supporting Information references:

[S6] Pettersson, S. Calculation of the thermal conductivity of alkali halide crystals. Journal of 285 Physics C: Solid State Physics 1987, 20, 1047.

[S7] Hakansson, B.; Ross, R. G. Thermal conductivity and heat capacity of solid LiBr and RbF under pressure. *Journal of Physics: Condensed Matter* 1989, *1*, 3977.

[S8] Wang, J.; Deng, M.; Chen, Y.; Liu, X.; Ke, W.; Li, D.; Dai, W.; He, K. Structural, elastic, electronic and optical properties of lithium halides (LiF, LiCl, LiBr, and LiI): First-principle calculations. *Materials Chemistry and Physics* 2020, 244, 122733.

[S9] Hill, R. The elastic behaviour of a crystalline aggregate. *Proceedings of the Physical Society*. Section A 1952, 65.

[S10] Marshall, B.; Cleavelin, C. Elastic constants of LiBr from 300° to 4.2° K. *Journal of Physics and Chemistry of Solids* 1969, 30, 1905–1908.

6. Applying pressures in the GPa range requires bulky mechanical components, which introduce significant dead thermal mass and compromise the efficacy of thermal switching. If the computational support were stronger, I do not think the authors would need to include this discussion to motivate their writing (this is a minor issue).

We agree with the reviewer that applying GPa-range pressures is impractical for real-world thermal switching applications due to the bulky mechanical components and associated thermal mass. Our intent in including that discussion was to contextualize our results within the broader literature, where the largest reported changes in thermal conductivity have been observed. In our work, we demonstrate that significant changes in thermal and mechanical properties are achievable under hydrostatic pressure. While such pressures are not directly applied for switching, our findings provide insight into the underlying mechanisms, particularly bonding environment changes, that could be leveraged in future studies to develop dynamic thermal materials using more practical stimuli (e.g., light, electric fields, or strain). We have revised the text to better reflect this motivation and to clarify that our focus is on uncovering fundamental behavior that could inspire application-relevant switching mechanisms.

In page 3 of the revised manuscript, we add,

"We note that while such pressures are not directly applicable for thermal switching applications, our findings provide insight into the underlying mechanisms, particularly bonding environment changes, that could be leveraged in future studies to develop dynamic thermal materials using more practical stimuli (e.g., light, electric fields, or strain).[1,25,26]"

Manuscript References:

[1] Wehmeyer, G.; Yabuki, T.; Monachon, C.;Wu, J.; Dames, C. Thermal diodes, regulators, and switches: Physical mechanisms and potential applications. *Applied Physics Reviews* 2017, 4, 041304.

[25] Liu, C.; Wu, C.; Zhao, Y.; Chen, Z.; Ren, T.-L.; Chen, Y.; Zhang, G. Actively and reversibly controlling thermal conductivity in solid materials. *Physics Reports* 2024, 1058, 1–32, Actively and reversibly controlling thermal conductivity in solid materials.

[26] Cheng, Y.; Wu, X.; Zhang, Z.; Sun, Y.; Zhao, Y.; Zhang, Y.; Zhang, G. Thermo-mechanical correlation in two-dimensional materials. ***Nanoscale*** **2021, 13, 1425–1442.**

7. Typo: BaAs should be BAs.

We thank the reviewer again for such a thorough review of our manuscript. We have corrected the typographical error and revised our manuscript for other such typos.

Reviewer: 2

Comments to the Authors

This manuscript presents a theoretical study on the impacts of pressure on the thermal conductivity of lithium halides. A two orders of magnitude change was reported, and the underlying mechanism was discussed. Overall, this paper is well-structured, innovative, and aligns with the basic interests of the research community. However, there are some aspects that need clarification, some of which may affect the overall quality. Therefore, I recommend publishing this paper after major revisions. The following are some issues that need attention:

We sincerely appreciate the reviewer's detailed evaluation and insightful suggestions, which have significantly improved the clarity and breadth of our manuscript as we detail below.

1. The change in thermal conductivity is attributed to bonding characteristic. In addition to this factor, geometry also affects the lifetime of phonon, then changes thermal conductivity, refer to National Science Review, 8(9), nwaa220, (https://doi.org/10.1093/nsr/nwaa220). The authors should check this point.

We thank the reviewer for the insightful comment. We have now incorporated a discussion of geometric effects on phonon transport in addition to bonding characteristics.

In page 9 of the revised manuscript, we state,

"It should be noted that along with changes in the bonding character, a recent work has shown that geometry effects such as bond angle and length changes can also significantly change thermal transport properties.[40] Future studies could explore the effects of uniaxial or shear strain, which—unlike the hydrostatic compression examined in our work—would alter bond lengths differently and offer insight into how such distortions influence the thermal properties of lithium halides."

Manuscript References:

[40] Ding, B.; Li, X.; Zhou, W.; Zhang, G.; Gao, H. Anomalous strain effect on the thermal conductivity of low-buckled two-dimensional silicene. *National Science Review* 2020, 8, nwaa220.

2. Although the results are promising, a discussion on the challenges in experimental observation can also be included.

We thank the reviewer for this thoughtful suggestion. We agree that the experimental measurement of thermal conductivity under high-pressure conditions presents considerable

challenges, and we have now included a brief discussion of these issues in the revised manuscript in page 17, where we state.

"The findings presented in this study highlight the need for experimental validation of thermal and mechanical property changes under high pressure, for instance, using diamond anvil cell techniques. However, measurements in diamond anvil cells are limited by several factors: the extremely small sample volumes (~10–100 µm scale) make it difficult to obtain accurate thermal data due to dominant boundary scattering effects;[5] thermal gradients can be poorly defined due to pressure-induced heterogeneities and non-hydrostatic stresses;[49] and the constrained geometry introduces additional uncertainties from heat loss through the gasket and diamond anvils. While techniques such as Time-Domain Thermoreflectance (TDTR) and Frequency-Domain Thermoreflectance (FDTR) show promise, they rely on smooth, uniform sample surfaces and stable transducer layers, conditions that lead to diminished signal quality in pump-probe experiments under high-pressure environments.[50] These challenges highlight the value of computational approaches, such as ours, which allow thermal transport to be investigated under controlled, idealized conditions that are difficult to replicate experimentally."

Manuscript References:

[5] Cahill, D. G.; Ford, W. K.; Goodson, K. E.; Mahan, G. D.; Majumdar, A.; Maris, H. J.; Merlin, R.; Phillpot, S. R. Nanoscale thermal transport. *Journal of Applied Physics* 2003, *93*, 793–818.

[49] Dewaele, A.; Datchi, F.; Loubeyre, P.; Mezouar, M. High pressure–high temperature equations of state of neon and diamond. *Phys. Rev. B* 2008, *77*, 094106.

[50] Hohensee, G. T.; Wilson, R. B.; Cahill, D. G. Thermal conductance of metal–diamond interfaces at high pressure. *Nature Communications* 2015, 6, 6578

3. Figure 4g presents the impact of pressure on temperature dependence. Detailed physical analysis should be provided, for example, the temperature dependent SED.

We thank the reviewer for the suggestion, and accordingly, we have carried out additional calculations of SEDs at higher temperatures, as shown in Figs. S26-S27 of the Supporting Information (and shown below for the reviewer's consideration). As is evident from the figure, at higher temperatures, the SEDs are broadened even more, and the system becomes more anharmonic with temperature, thus leading to higher scattering of phonons and lowered thermal conductivity. This broadening is comparatively more pronounced for the pressurized case, where the well-defined phonon branches at the low temperature (100 K) are considerably broadened at the higher temperatures as shown below for the reviewers consideration.

We have added the following discussion in page 13 of our revised manuscript:

"We have performed additional SED calculations at elevated temperatures under both ambient and high-pressure conditions. As shown in Fig. S26, increasing the temperature results in further broadening of the SEDs, indicating enhanced anharmonicity in the system. This increased anharmonicity leads to stronger phonon scattering and, consequently, reduced thermal conductivity. The effect is even more significant under pressure, as highlighted in Fig. S27, showing that the well-defined phonon branches at the low temperature (100 K) is considerably broadened at higher temperatures."

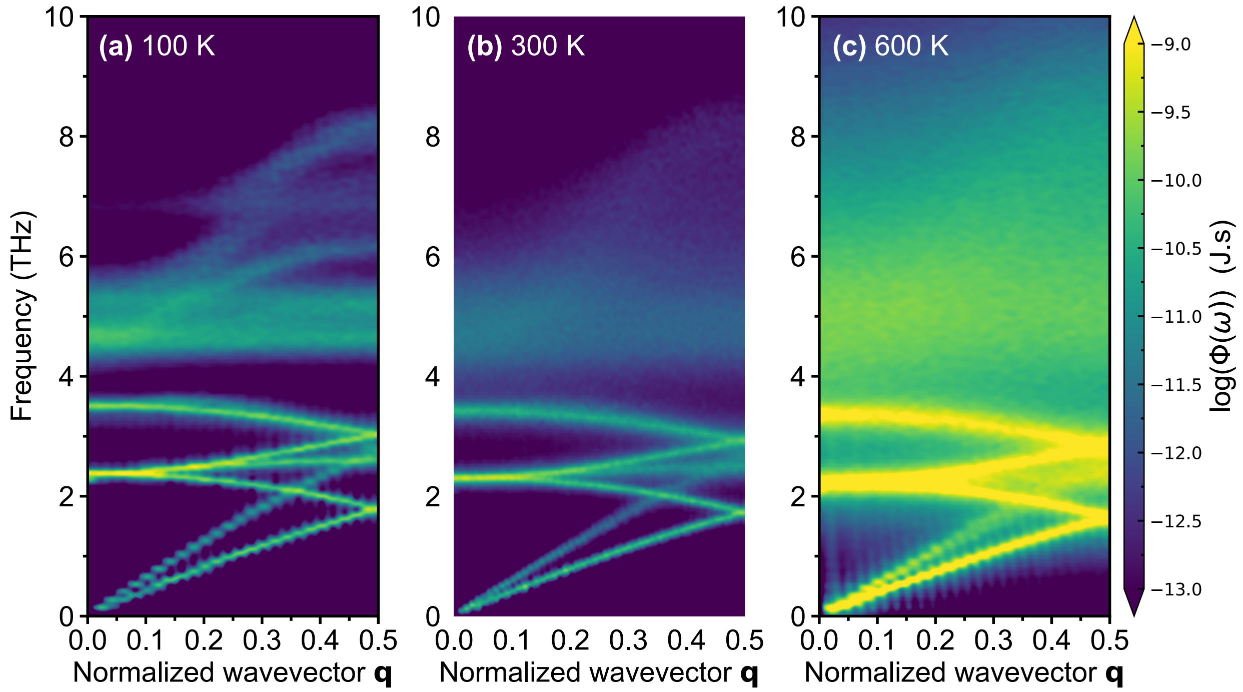

Figure S26: Calculated phonon spectral energy densities (SEDs) for LiBr at 0 GPa for (a) 100 K, (b) 300 K, and (c) 600 K. The progressive broadening of the SED peaks with increasing temperature indicates enhanced anharmonicity, stronger phonon-phonon scattering, and reduced phonon lifetimes resulting in lower thermal conductivity.

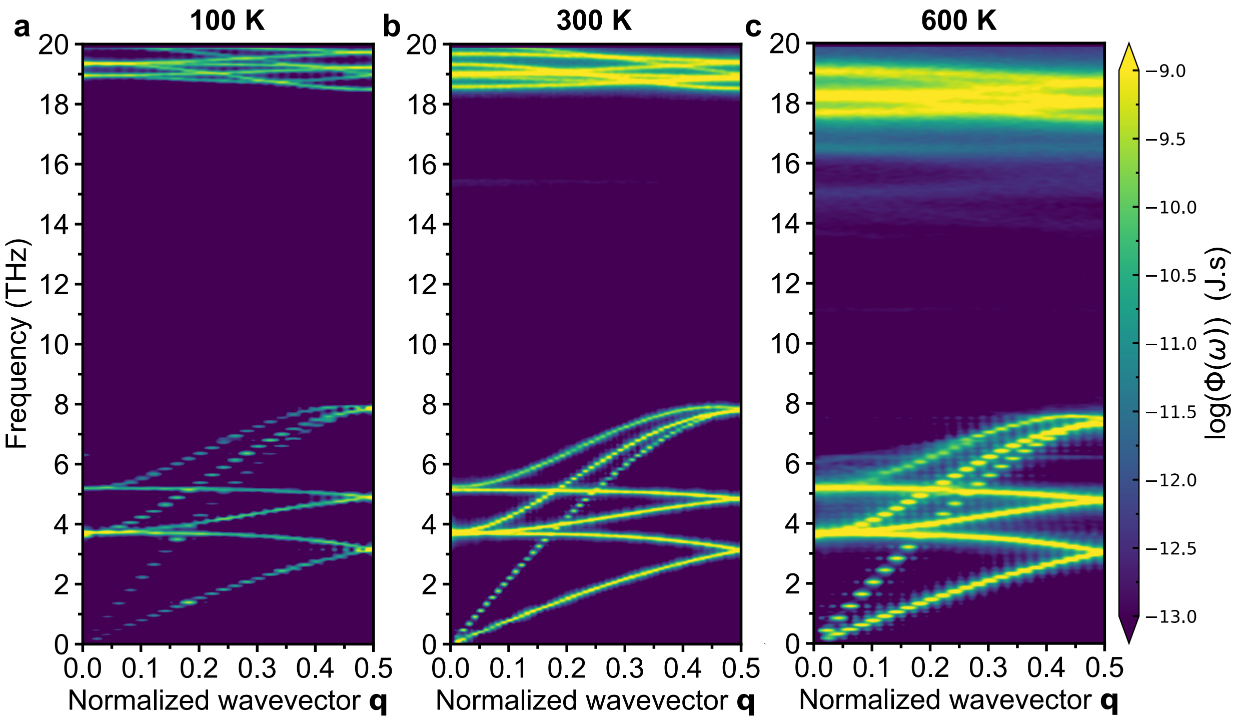

Figure S27: Calculated phonon spectral energy densities (SEDs) for LiBr at 90 GPa for (a) 100 K, (b) 300 K, and (c) 600 K. The progressive broadening of the SED peaks with increasing temperature indicates enhanced anharmonicity, stronger phonon-phonon scattering, and reduced phonon lifetimes resulting in lower thermal conductivity.

4. Active control of thermal conductivity is a hot topic in recent years, I noticed that there is room for further citation of articles closely related to the research topic, for example, Nanoscale, 13, 1425 (2021); Physics Reports 1058, 1–32 (2024). This will help enrich the content of the paper, enhance its academic value, and make it easier for readers to understand the perspective.

We thank the reviewer for this valuable suggestion to strengthen our literature review on active thermal conductivity control. In light of reviewer's suggestion, we have incorporated the suggested references (*Nanoscale*, 13, 1425 (2021) and *Physics Reports* 1058, 1–32 (2024)) in our revised manuscript. These articles will allow more comprehensive perspective on recent advances in active thermal transport control and to better contextualize our work within the broader research landscape.

Reviewer: 3

Comments to the Authors

In the manuscript, the authors have investigated the thermal conductivity and bulk modulus of LiI and LiBr by using molecular dynamics (MD) simulations together with machine learning potentials (MLPs). This paper is well written, but there are some issues needed to be addressed.

The authors express their sincere gratitude to the reviewer for conducting an in-depth review of the manuscript and for recognizing the significance of the results presented in the manuscript. We have revised our manuscript in accordance with the reviewers' suggestions as detailed below.

1. The accuracy of the present MLPs needs some validation. The authors can present a comparison of the thermal conductivity and bulk modulus extracted from the present MLPs and density functional theory calculations or experiments.

We thank the reviewer for the thoughtful comment regarding the validation of our MLPs quantitatively. We appreciate the reviewer's suggestion to include a concise comparison with prior results for both bulk moduli and thermal conductivity. We have compared our thermal conductivity as well as bulk moduli results with prior experimental values as shown in Fig. S10 in supporting information and attached below for the reviewer's consideration. Additionally, we have referenced a number of solid benchmarking works that have employed our MLP approach to assess thermal and structural properties in multitude of material systems.

In our revised supplemental material in page S17, we state:

"Our machine learning potential (MLP) demonstrates strong predictive capability for both the mechanical and thermal properties of lithium halides, yielding values in close quantitative agreement with prior experimental studies, as shown in Fig. S10. For LiBr, the MLP predicts a bulk modulus of 28.02 GPa, which is remarkably consistent with DFT (29.94 GPa), experimental results (28.5 GPa), and earlier theoretical reports (26.3 GPa) using Voigt-Reuss-Hill method. Likewise, for LiI, the predicted bulk modulus of 22.73 GPa aligns well with DFT (22.67 GPa) and theoretical estimates (22.0 GPa).[S8–S10]

Beyond elasticity, the MLP captures thermal transport trends with similar accuracy, predicting a thermal conductivity of 1.14 W m^{-1} K^{-1} for LiBr. This value is in reasonable agreement with the experimental measurement of 1.8 W m^{-1} K^{-1} and matches the theoretical value of 1.31 W m^{-1} K^{-1} within the reported 15–20% statistical uncertainties.[S6,S7] The close agreement across DFT, experimental, and theoretical benchmarks confirms the robustness

of our MLP and supports its application for extended simulations and predictive studies of lithium halides."

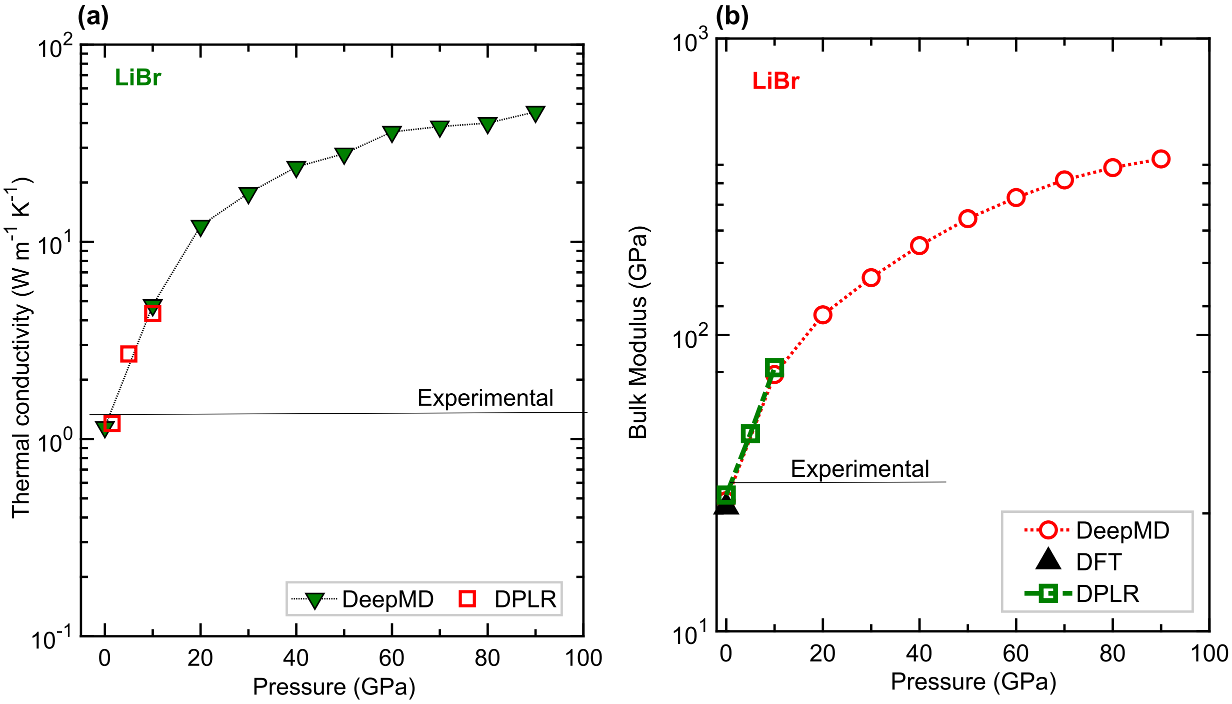

Figure S11: We developed a new potential using the Deep Potential Long-Range (DPLR) framework, which explicitly includes long-range electrostatic interactions for the low-pressure regime. At low pressures, where the system exhibits polar and ionic characteristics, we observe strong consistency in the predictions of (a) thermal conductivity and (b) bulk modulus between the DPLR and DeepMD models. Additionally, our computational results are supported by experimental data obtained under ambient conditions.[S7-S10]

Supporting Information references:

[S7] Hakansson, B.; Ross, R. G. Thermal conductivity and heat capacity of solid LiBr and RbF under pressure. *Journal of Physics: Condensed Matter* 1989, *1*, 3977.

[S8] Wang, J.; Deng, M.; Chen, Y.; Liu, X.; Ke, W.; Li, D.; Dai, W.; He, K. Structural, elastic, electronic and optical properties of lithium halides (LiF, LiCl, LiBr, and LiI): First-principle calculations. *Materials Chemistry and Physics* 2020, 244, 122733.

[S9] Hill, R. The elastic behaviour of a crystalline aggregate. *Proceedings of the Physical Society*. Section A 1952, 65.

[S10] Marshall, B.; Cleavelin, C. Elastic constants of LiBr from 300° to 4.2° K. *Journal of Physics and Chemistry of Solids* 1969, 30, 1905–1908.

2. The method employed here to calculate the bulk modulus was based on the slope of two points in the P-V curve. This method is too rough. I recommend the authors to try some other methods such as the strain-fluctuation methods.

We acknowledge the reviewer's suggestion regarding alternative methods to validate our bulk modulus calculations. We have performed additional calculations of bulk modulus through a different approach based on the Equation of State (EOS)[1-3], which agrees very well with our technique. However, we note that our method of determining the bulk modulus from the P-V curve is also a standard technique.[4-6] The bulk modulus is predicted from a linear fit to multiple P-V data points near equilibrium typically 50-100 data points as shown below. Our P-V data was generated by equilibrating the system at ambient temperature and under a barostat followed by sampling pressures at varying volumes. This approach ensures that the bulk modulus reflects the material's response over a range of pressures. While simpler than strain-fluctuation methods, this method is computationally efficient and sufficiently accurate for our purposes, as demonstrated by its agreement with experimental as well as theoretical values in lithium halides. [7-9]

Moreover, we were cautious and conducted size-effect tests by replicating the system into larger supercells and confirmed that our bulk modulus calculations remained consistent by ruling any finite-size artifacts as shown in underlying Figure S15 shown below for the reviewer's consideration.

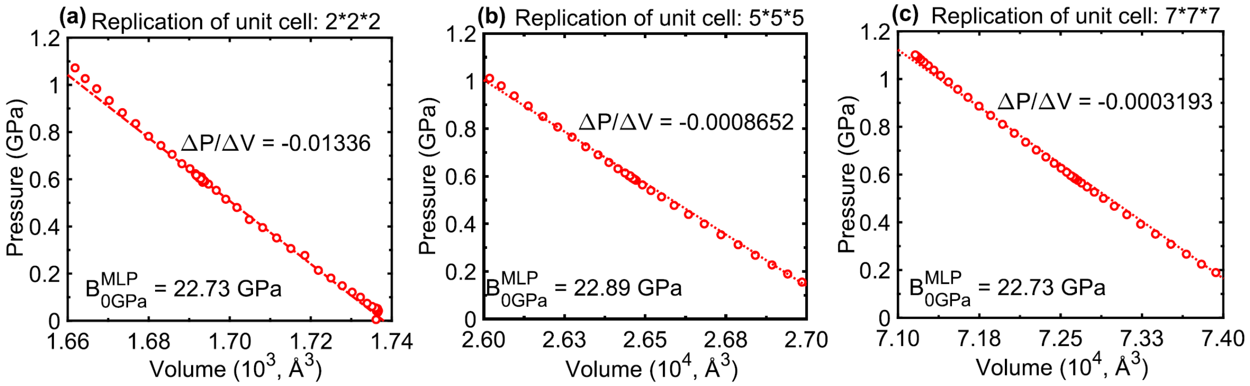

Figure S15: MLP-MD predicted bulk modulus for LiI as a function of computational domain size. We notice that all of our bulk modulus calculations demonstrate convergence within the limits of statistical uncertainties, regardless of the chosen domain size.

In light of the reviewer's suggestion, we have added following discussion in our revised supporting information in page S17 to further reinforce the robustness of our approach as:

"We also calculated bulk modulus for LiBr at ambient using equation of state approach as shown in Fig. S16 to reinforce the robustness of our P-V approach which is a fundamentally standard technique.[S11,S27,S28] The bulk modulus is computed using the equation of state (EOS) approach,[S12–S14] implemented through a multi-step workflow in LAMMPS.[S29] This procedure involves sequential energy minimizations and systematic volume perturbations to evaluate the mechanical response of the system. Initially, the structure is relaxed using a two-

stage energy minimization protocol that combines the conjugate gradient (CG)[S30] and fast inertial relaxation engine (FIRE)[S31] algorithms to ensure convergence on the potential energy surface. Following structural relaxation, a series of volume perturbations are applied under NPT ensemble to generate an energy–volume (E–V) dataset. The resulting E–V data are then fitted to the Murnaghan equation of state. [S12,S14] The agreement of bulk modulus using our standard P-V approach with experimental, DFT-based, theoretical as well as EOS approach further reinforces the robustness of our approach."

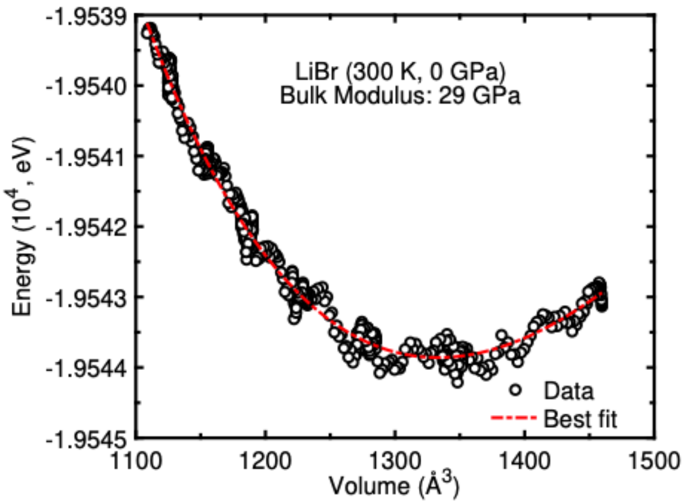

Figure S16: Calculated bulk modulus for LiBr at 0 GPa using the machine learned interatomic potential. The dashed lines represent fits to the Birch-Murnaghan equation of state (EOS).[S12-S14]

Review References:

[1] Murnaghan, F. D. The compressibility of media under extreme pressures. *Proceedings of the National Academy of Sciences* 1944, 30, 244–247.

[2] Birch, F. Finite elastic strain of cubic crystals. *Physical review* 1947, 71, 809.

[3] Tyuterev, V.; Vast, N. Murnaghan's equation of state for the electronic ground state energy. *Computational materials science* 2006, 38, 350–353.

[4] Kittel, C.; McEuen, P. *Introduction to solid state physics*; John Wiley & Sons, 2018.

[5] Odegard, G. M.; Patil, S. U.; Deshpande, P. P.; Kanhaiya, K.; Winetrout, J. J.; Heinz, H.; Shah, S. P.; Maiaru, M. Molecular dynamics modeling of epoxy resins using the reactive interface force field. *Macromolecules* 2021, 54, 9815–9824.

[6] Kashmari, K.; Patil, S. U.; Kemppainen, J.; Shankara, G.; Odegard, G. M. Optimal molecular dynamics system size for increased precision and efficiency for epoxy materials. *The Journal of Physical Chemistry B* 2024, 128, 4255–4265.

[7] Wang, J.; Deng, M.; Chen, Y.; Liu, X.; Ke, W.; Li, D.; Dai, W.; He, K. Structural, Elastic, Electronic and Optical Properties of Lithium Halides (LiF, LiCl, LiBr, and LiI): First-Principle Calculations. *Mater. Chem. Phys. 2020*, 244, 122733.

[8] Hill, R. The Elastic Behaviour of a Crystalline Aggregate. Proc. Phys. Soc. Sect. A 1952, 65 (5), 349.

[9] Marshall, B. J.; Cleavelin, C. R. Elastic Constants of LiBr from 300° to 4.2°K. J. Phys. Chem. Solids 1969, 30 (8), 1905–1908.

Supporting Information References:

[S12] Murnaghan, F. D. The compressibility of media under extreme pressures. *Proceedings of the National Academy of Sciences* 1944, 30, 244–247.

[S13] Birch, F. Finite elastic strain of cubic crystals. *Physical review* 1947, 71, 809.

[S14] Tyuterev, V.; Vast, N. Murnaghan's equation of state for the electronic ground state energy. *Computational materials science* 2006, 38, 350–353.

3. The thermal conductivity and bulk modulus similarly increase with growing pressure. Do there exist some relevance between these two parameters at different pressures?

The reviewer is absolutely correct that there exists a strong relationship between bulk modulus and thermal conductivity. This can be quantitatively evaluated using the well-established Liebfried and Schlömann (LS) model. We have disscussed this in detail in section S6 in our supporting information. Under isochoric and isotropic conditions, and assuming minimal changes in Poisson ratio and elastic anisotropy with pressure, the LS model simplifies to an analytical expression of the form, $\kappa \propto \mathrm{A} \cdot K_T^{3/2}$. This indicates a direct and theoretically grounded dependence of thermal conductivity on the bulk modulus.

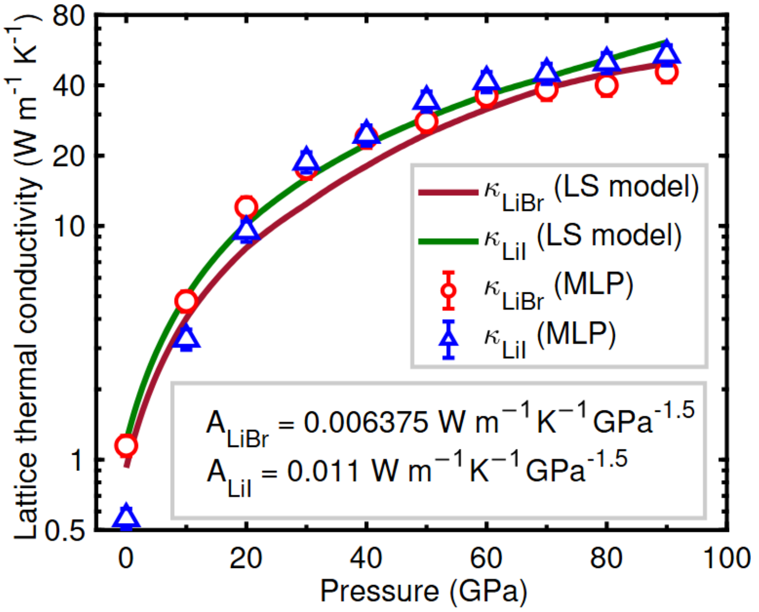

Figure S34: Comparison of our MLP-based pressure dependence of thermal conductivity for lithium halides (LiBr and LiI) with that estimated using Liebfried and Schlömann (LS) equation. There is a good agreement between our MLP-based predictions and LS estimates within statistical uncertainties.

In page 15 of the main manuscript, we add,

"The significant role of acoustic modes is further evidenced by the linear correlation observed between pressure and bulk modulus, consistent with the analytical strain-dependent thermal conductivity model proposed by Leibfried and Schlömann,[47] which predicts $\kappa \propto K^{3/2}$, where K is the bulk modulus (see Fig. S34). This framework assumes that heat transport is primarily governed by acoustic phonons. Therefore, the close agreement between theoretical predictions and molecular dynamics simulations supports the conclusion that acoustic phonons are the primary heat carriers in lithium halides."

4. How about the bulk modulus at different temperature?

We thank the reviewer for this insightful comment regarding the temperature dependence of the bulk modulus in LiBr. We investigated the temperature dependence of the bulk modulus in lithium bromide (LiBr) across both ambient and extreme pressures, drawing comparisons with other alkali halides and diamond. Our results reveal that at 90 GPa, the bulk modulus remains nearly constant with changing temperature. Conversely, at ambient pressure, we observe a linear decrease, a trend consistent with other halides (namely KCl and NaCl) as shown below for the reviewer's consideration (Fig. S16).

The bulk modulus of a material typically decreases with increasing temperature. As temperature increases, atoms in a solid vibrate more vigorously and tend to move farther apart on average. This leads to thermal expansion, which means the material becomes less dense and more compliant (easier to compress). This effectively softens the material and lowers the bulk modulus leading to a linear decrease in bulk modulus.[10] However, for a covalent solid such as diamond, the bulk modulus remains constant since the thermal expansion is limited due to the strong nature of the covalent bonds (as shown in the figure). This is consistent with our lithium halides at high pressure, where the material becomes covalently bonded and thus the bulk modulus does not change with temperature.

In response to the reviewer's question, we have added the following discussion to our revised manuscript in page 17, where we state,

"Finally, we also computed the bulk modulus of the lithium halides across a range of temperatures to further support the observation of a transition from ionic to covalent bonding. At ambient pressure, the bulk modulus shows a linear decrease with increasing temperature, as seen in Fig. S17, consistent with typical ionic solids. This trend arises because rising temperatures cause atoms to vibrate more intensely and move further apart on average, leading to thermal expansion and increased compressibility. As a result, the material softens and the bulk modulus decreases. In contrast, for strongly covalent materials such as diamond, the bulk modulus remains largely unchanged with temperature due to

minimal thermal expansion, a result of the robustness of covalent bonds. This behavior is also observed in our high-pressure lithium halide calculations, where the temperature dependence of the bulk modulus diminishes, indicating a shift toward covalent bonding at elevated pressures."

In Supporting Information in page S18, we state:

"We also examined the temperature dependence of the bulk modulus in LiBr under both ambient and extreme pressures, comparing our findings with those of other alkali halides and diamond. Notably, at 90 GPa, the bulk modulus shows a near-constant behavior as temperature varies, demonstrating remarkable stability under high pressure. In contrast, at ambient pressure, we observe a subtle yet consistent linear decline, mirroring trends seen in other halides as evidenced in Fig. S17."

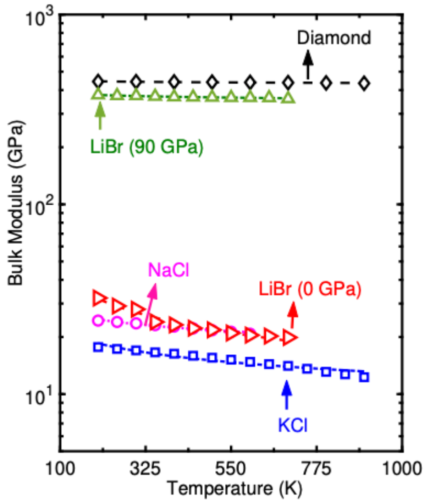

Figure S17: Temperature dependence of the bulk modulus in LiBr examined at both ambient and extreme pressures, and compared with other alkali halides and diamond.[S32] At 90 GPa, we observe that the bulk modulus remains nearly constant with temperature, while at ambient pressure, there is a very weak, monotonic linear decrease, which is similar to the trend seen in other halides.[S33]

Supporting Information references:

[S32] Jacobson, P.; Stoupin, S. Thermal expansion coefficient of diamond in a wide temperature range. *Diamond and Related Materials* 2019, 97, 107469.

[S33] Madan, M. Temperature dependence of the bulk modulus of alkali halides. *Journal of Applied Physics* 1971, 42, 3888–3893.

Review References:

[10] Madan, M. Temperature dependence of the bulk modulus of alkali halides. *Journal of Applied Physics* 1971, 42, 3888–3893.

5. It is surprising that the bulk moduli of LiI and LiBr significantly increase with growing pressure. The authors should give some explanation to this phenomenon.

We believe that the massive changes in the bulk modulus derives from the fact that the materials transition from an ionic (weakly bonded) to a covalent strongly bonded solid through the application of pressure. To provide further support for this, we have carried out Crystal Orbital Hamilton Population (COHP) which can give insights into the changes in the bonding nature in a quantitative sense. As detailed below for the reviewer, this method allows us to quantitatively show that the bonding is drastically increased in these solids, thus leading to the massive enhancement in the bulk modulus.

In the Supporting Infomation, we have added the following discussion in page S24 as follows:

"We investigate the influence of pressure on the chemical binding nature in LiBr and LiI using the Crystal Orbital Hamilton Population (COHP) method as implemented in the LOBSTER code.[S44,S45] COHP allows the decomposition of the electronic band energy into individual atomic or orbital interactions, classifying them as bonding (positive), anti-bonding (negative), or non-bonding (zero).[S44,S45] Our calculations reveal that as pressure increases, the bonding interactions become stronger, indicating enhanced covalent character, which further supports our hypothesis."

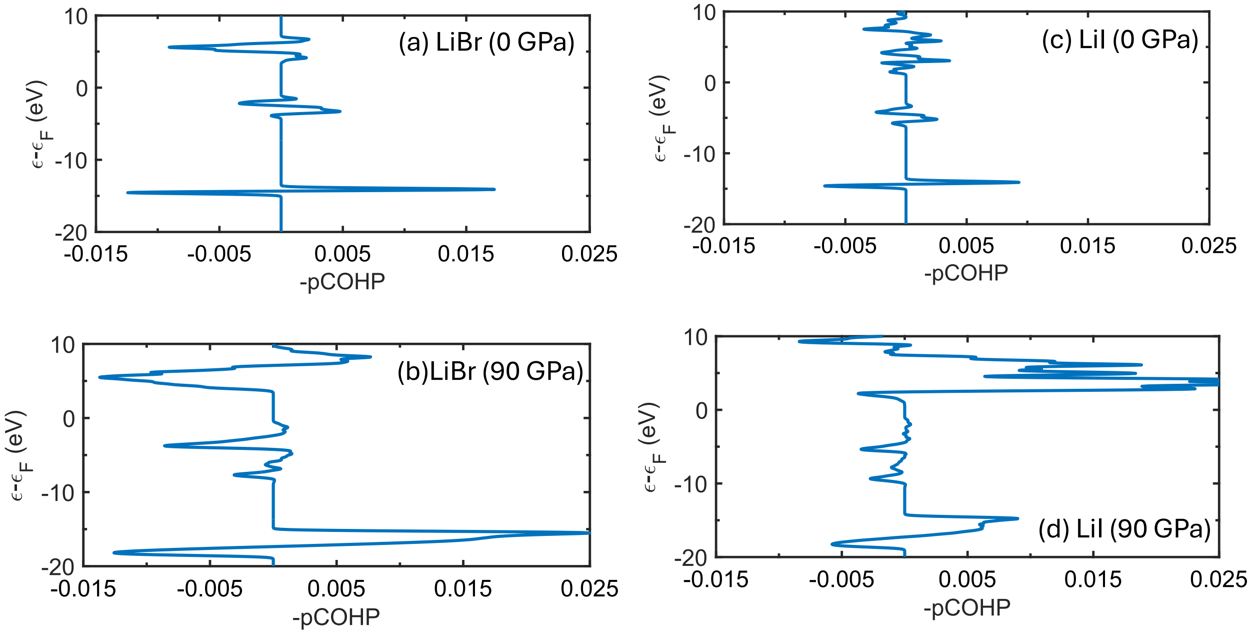

Figure S24: Crystal orbital Hamiltonian Population (COHP) for LiBr and LiI at 0 and 90 GPa, which quantifies bonding (positive), anti-bonding (negative), or non-bonding (zero) characteristics for the electronic energies. The COHP calculation shows that the bonding characteristic drastically increases at high pressures.

Supporting Information References:

[S44] Deringer, V. L., Tchougréeff, A. L. & Dronskowski, R. Crystal Orbital Hamilton Population (COHP) Analysis As Projected from Plane-Wave Basis Sets. *J. Phys. Chem. A* 115, 5461-5466 (2011).

[S45] Dronskowski, R. & Bloechl, P. E. Crystal orbital Hamilton populations (COHP): energy-resolved visualization of chemical bonding in solids based on density-functional calculations. *J. Phys. Chem*. 97, 8617-8624 (1993).

We also run additional calculations of bulk modulus at different temperatures to show that the material is transitioning from an ionic to a covalent solid, which can be inferred from the temperature dependence as discussed in the revised manuscript in page 17:

"Finally, we also computed the bulk modulus of the lithium halides across a range of temperatures to further support the observation of a transition from ionic to covalent bonding. At ambient pressure, the bulk modulus shows a linear decrease with increasing temperature, as seen in Fig. S17, with typical ionic solids. This trend arises because rising temperatures cause atoms to vibrate more intensely and move further apart on average, leading to thermal expansion and increased compressibility. As a result, the material softens and the bulk modulus decreases. In contrast, for strongly covalent materials such as diamond, the bulk modulus remains largely unchanged with temperature due to minimal thermal expansion, a result of the robustness of covalent bonds. This behavior is also observed in our high-pressure lithium halide calculations, where the temperature dependence of the bulk modulus diminishes, indicating a shift toward covalent bonding at elevated pressures."

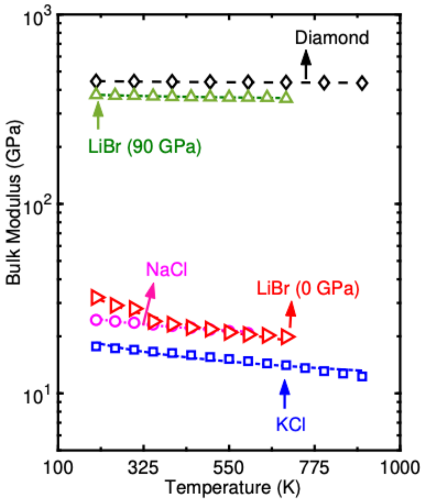

Figure S17: Temperature dependence of the bulk modulus in LiBr examined at both ambient and extreme pressures, and compared with other alkali halides and diamond.[S32] At 90 GPa, we observe that the bulk modulus remains nearly constant with temperature, while at ambient pressure, there is a very weak, monotonic linear decrease, which is similar to the trend seen in other halides.[S33]

jz-2025-01476q.R2

Name: Peer Review Information for "Achieving exceptionally enhanced thermal conductivity and bulk modulus in polar insulators via modification of chemical bonding"

Second Round of Reviewer Comments

Reviewer: 2

Comments to the Author

The authors addressed referee's comment and concern, provided a detailed response letter. The quality of the manuscript is improved. It is acceptable.

Reviewer: 1

Comments to the Author

I applaud the authors' efforts in implementing the DPLR (J. Chem. Phys. 156, 124107) modeling. However, I do not think they have conducted enough research to draw a reliable conclusion.

First of all, the new calculation (fig. s11) never reaches the high pressure regime. I do not understand the reason that prevents this calculation. Secondly, in the original paper that introduced DPLR, the authors emphasize the size effect in the modeling. In the current draft, the author does not provide any detailed report on the quality of the phonon property calculation except for the force constants residuals, which can always be minimized below a decent value. I do not know if the phonon dispersion can be correctly recreated by DPLR, let alone the follow-up calculation on thermal conductivity.

Since the authors want to present a ratio of thermal conductivity rather than just an order of magnitude estimation, the accuracy of the calculation can only be guaranteed by state-of-the-art methodologies (which were listed in my previous report), and Deep Potential is not necessarily the best approach for this simple solid-state binary material. Unfortunately, the current results cannot convince me and I am sorry that I cannot respond more positively this time.

Author's Response to Peer Review Comments:

Reviewer: 2

Recommendation: This paper represents a significant new contribution and should be published as is.

Comments:
The authors addressed referee's comment and concern, provided a detailed response letter. The quality of the manuscript is improved. It is acceptable.

We sincerely thank the reviewer for their thorough evaluation and for recognizing the significance of our results. Based on the insightful feedback provided in the previous review, we have broadened the scope of our study. We believe this expanded perspective will attract interest from researchers across multiple disciplines, enabling them to leverage the insights presented here to advance various fields that depend on tuning material properties within a single material system.

Reviewer: 1

Recommendation: Reconsider as an article in The Journal of Physical Chemistry A/B/C.

Comments:
I applaud the authors' efforts in implementing the DPLR (J. Chem. Phys. 156, 124107) modeling. However, I do not think they have conducted enough research to draw a reliable conclusion.

We respectfully disagree with the reviewer's assessment that our study lacks sufficient depth to support a reliable conclusion. This work represents the combined efforts of three graduate students with complementary expertise in thermal transport, mechanical properties, density functional theory (DFT), molecular dynamics (MD), and atomistic simulations.

Our methodology leverages the DPLR approach (J. Chem. Phys. 156, 124107) in conjunction with state-of-the-art first-principles and atomistic modeling tools that have been extensively validated across multiple materials classes and application domains. To ensure robustness, we have cross-verified our findings using multiple independent computational frameworks, examining consistency in both thermal and mechanical property predictions.

The key conclusion—that lithium halides can exhibit exceptionally large and tunable changes in both thermal conductivity and bulk modulus through modifications of their bonding configuration—is not only supported by rigorous simulation protocols but also represents a novel insight not previously reported in the literature. Our parameter space exploration included variations in lattice strain, bonding geometry, and chemical environment, enabling us to establish clear structure–property relationships.

We believe the combination of methodological rigor, multi-approach validation, and unprecedented findings provides a strong and reliable basis for the conclusions we present.

We appreciate the reviewer's comments and the opportunity to clarify our methodology and scope.

1. Pressure range limitation

The reason we did not extend the DPLR calculations to the highest pressure regimes is twofold:

(a) Computational cost and review timeline: The DPLR method, while highly accurate for treating long-range electrostatics, is substantially more computationally intensive than DeePMD alone, especially for the large supercells and long trajectories required for converged thermal transport calculations. Within the review timeframe, performing DPLR simulations across the full pressure range was not feasible without compromising convergence and statistical accuracy.

(b) Relevance to bonding regime: Our primary interest in applying DPLR is for pressure ranges where the material is polar and long-range Coulomb interactions play a significant role in governing phonon dynamics—this is the low- to intermediate-pressure regime where electrons are localized around halide anions. At higher pressures, as the system transitions to a more covalent bonding state, the contribution of long-range interactions diminishes considerably. In these regimes, DeePMD is sufficient to capture the essential physics of both bulk modulus and thermal conductivity.

2. Temperature effects and comparison to experiment

Unlike static 0 K DFT phonon calculations, our molecular dynamics framework inherently incorporates finite-temperature effects, allowing us to account for phonon-phonon scattering processes that are essential for realistic thermal conductivity predictions. Importantly, our calculated thermal conductivities and bulk moduli match experimental measurements within reported uncertainties. This agreement is not incidental—it stems from the fact that our DPLR and DeePMD models were trained directly on high-quality ab initio data, ensuring transferability across the relevant thermodynamic range.

3. Broader focus beyond thermal conductivity

We emphasize that the novelty of our work lies not only in thermal transport predictions but also in demonstrating large, pressure-induced changes in mechanical properties arising from bonding rearrangements in lithium halides. These findings are unprecedented and have been validated through multiple, independent computational approaches.

In summary, while we acknowledge that DPLR calculations at the extreme high-pressure limit were not performed, the methodology used in the pressure regimes where long-range effects matter is robust, validated, and experimentally consistent. The combined evidence from phonon dispersion matching, thermal transport agreement with experiment, and cross-method verification gives us high confidence in the reliability of our conclusions.
